# ﻿A generic classification of Xenidae (Strepsiptera) based on the morphology of the female cephalothorax and male cephalotheca with a preliminary checklist of species

**DOI:** 10.3897/zookeys.1093.72339

**Published:** 2022-04-07

**Authors:** Daniel Benda, Hans Pohl, Yuta Nakase, Rolf Beutel, Jakub Straka

**Affiliations:** 1 Department of Zoology, Faculty of Science, Charles University, Prague, Czech Republic Charles University Prague Czech Republic; 2 Department of Entomology, National Museum, Prague, Czech Republic National Museum Prague Czech Republic; 3 Institut für Zoologie und Evolutionsforschung, Friedrich-Schiller-Universität, Jena, Germany Friedrich-Schiller-Universität Jena Germany; 4 Department of Biology, Faculty of Science, Shinshu University, Matsumoto, Japan Shinshu University Matsumoto Japan

**Keywords:** Cephalotheca, cephalothorax, generic revision, morphology, Strepsiptera, taxonomy, wasp parasite, wasps, Xenidae

## Abstract

The generic taxonomy and host specialization of Xenidae have been understood differently by previous authors. Although the recent generic classification has implied a specialization on the level of host families or subfamilies, the hypothesis that each xenid genus is specialized to a single host genus was also previously postulated. A critical evaluation of the classification of the genera of Xenidae is provided here based on morphology in accordance with results of recent molecular phylogenetic studies. External features of the female cephalothoraces and male cephalothecae were documented in detail with different techniques. Diagnoses and descriptions are presented for all 13 delimited genera. The earliest diverging genera are usually well characterized by unique features, whereas deeply nested genera are usually characterized by combinations of characters. Three new genera are described: *Sphecixenos***gen. nov.**, *Tuberoxenos***gen. nov.**, and *Deltoxenos***gen. nov.** Five previously described genera are removed from synonymy: *Tachytixenos* Pierce, 1911, **stat. res.**; *Brasixenos* Kogan & Oliveira, 1966, **stat. res.**; *Leionotoxenos* Pierce, 1909, **stat. res.**; *Eupathocera* Pierce, 1908, **stat. res.**; and *Macroxenos* Schultze, 1925, **stat. res.** One former subgenus is elevated to generic rank: *Nipponoxenos* Kifune & Maeta, 1975, **stat. res.***Monobiaphila* Pierce, 1909, **syn. nov.** and *Montezumiaphila* Brèthes, 1923, **syn. nov.** are recognized as junior synonyms of *Leionotoxenos* Pierce, 1909, **stat. res.***Ophthalmochlus* Pierce, 1908, **syn. nov.**, *Homilops* Pierce, 1908, **syn. nov.**, *Sceliphronechthrus* Pierce, 1909, **syn. nov.**, and Ophthalmochlus (Isodontiphila) Pierce, 1919, **syn. nov.** are recognized as junior synonyms of *Eupathocera* Pierce, 1908, **stat. res.** A preliminary checklist of 119 described species of Xenidae with information on their hosts and distribution is provided. The following 14 species are recognized as valid and restituted from synonymy: *Tachytixenosindicus* Pierce, 1911, **stat. res.**; *Brasixenosacinctus* Kogan & Oliveira, 1966, **stat. res.**; *Brasixenosaraujoi* (Oliveira & Kogan, 1962), **stat. res.**; *Brasixenosbahiensis* Kogan & Oliveira, 1966, **stat. res.**; *Brasixenosbrasiliensis* Kogan & Oliveira, 1966, **stat. res.**; *Brasixenosfluminensis* Kogan & Oliveria, 1966, **stat. res.**; *Brasixenosmyrapetrus* Trois, 1988, **stat. res.**; *Brasixenoszikani* Kogan & Oliveira, 1966, **stat. res.**; *Leionotoxenoshookeri* Pierce, 1909, **stat. res.**; *Leionotoxenosjonesi* Pierce, 1909, **stat. res.**; *Leionotoxenoslouisianae* Pierce, 1909, **stat. res.**; *Eupathoceraluctuosae* Pierce, 1911, **stat. res.**; *Eupathoceralugubris* Pierce, 1909, **stat. res.**; *Macroxenospiercei* Schultze, 1925, **stat. res.** New generic combinations are proposed for 51 species: *Leionotoxenosarvensidis* (Pierce, 1911), **comb. nov.**; *Leionotoxenosbishoppi* (Pierce, 1909), **comb. nov.**; *Leionotoxenosforaminati* (Pierce, 1911), **comb. nov.**; *Leionotoxenosfundati* (Pierce, 1911), **comb. nov.**; *Leionotoxenoshuastecae* (Székessy, 1965), **comb. nov.**; *Leionotoxenositatiaiae* (Trois, 1984), **comb. nov.**; *Leionotoxenosneomexicanus* (Pierce, 1919), **comb. nov.**; *Leionotoxenosprolificum* (Teson & Remes Lenicov, 1979), **comb. nov.**; *Leionotoxenosrobertsoni* (Pierce, 1911), **comb. nov.**; *Leionotoxenostigridis* (Pierce, 1911), **comb. nov.**; *Leionotoxenosvigili* (Brèthes, 1923), **comb. nov.**; *Eupathoceraargentina* (Brèthes, 1923), **comb. nov.**; *Eupathoceraauripedis* (Pierce, 1911), **comb. nov.**; *Eupathocerabucki* (Trois, 1984), **comb. nov.**; *Eupathoceraduryi* (Pierce, 1909), **comb. nov.**; *Eupathoceraerynnidis* (Pierce, 1911), **comb. nov.**; *Eupathocerafasciati* (Pierce, 1909), **comb. nov.**; *Eupathocerafuliginosi* (Brèthes, 1923), **comb. nov.**; *Eupathocerainclusa* (Oliveira & Kogan, 1963), **comb. nov.**; *Eupathocerainsularis* (Kifune, 1983), **comb. nov.**; *Eupathoceramendozae* (Brèthes, 1923), **comb. nov.**; *Eupathocerapiercei* (Brèthes, 1923), **comb. nov.**; *Eupathocerastriati* (Brèthes, 1923), **comb. nov.**; *Eupathocerataschenbergi* (Brèthes, 1923), **comb. nov.**; *Eupathocerawestwoodii* (Templeton, 1841), **comb. nov.**; *Macroxenospapuanus* (Székessy, 1956), **comb. nov.**; *Sphecixenosabbotti* (Pierce, 1909), **comb. nov.**; *Sphecixenosastrolabensis* (Székessy, 1956), **comb. nov.**; *Sphecixenosdorae* (Luna de Carvalho, 1956), **comb. nov.**; *Sphecixenoserimae* (Székessy, 1956), **comb. nov.**; *Sphecixenosesakii* (Hirashima & Kifune, 1962), **comb. nov.**; *Sphecixenosgigas* (Pasteels, 1950), **comb. nov.**; *Sphecixenoskurosawai* (Kifune, 1984), **comb. nov.**; *Sphecixenoslaetum* (Ogloblin, 1926), **comb. nov.**; *Sphecixenosorientalis* (Kifune, 1985), **comb. nov.**; *Sphecixenosreticulatus* (Luna de Carvalho, 1972), **comb. nov.**; *Sphecixenossimplex* (Székessy, 1956), **comb. nov.**; *Sphecixenosvanderiisti* (Pasteels, 1952), **comb. nov.**; *Tuberoxenosaltozambeziensis* (Luna de Carvalho, 1959), **comb. nov.**; *Tuberoxenossinuatus* (Pasteels, 1956), **comb. nov.**; *Tuberoxenossphecidarum* (Siebold, 1839), **comb. nov.**; *Tuberoxenosteres* (Pasteels, 1950), **comb. nov.**; *Tuberoxenostibetanus* (Yang, 1981), **comb. nov.**; *Deltoxenosbequaerti* (Luna de Carvalho, 1956), **comb. nov.**; *Deltoxenosbidentatus* (Pasteels, 1950), **comb. nov.**; *Deltoxenoshirokoae* (Kifune & Yamane, 1992), **comb. nov.**; *Deltoxenosiwatai* (Esaki, 1931), **comb. nov.**; *Deltoxenoslusitanicus* (Luna de Carvalho, 1960), **comb. nov.**; *Deltoxenosminor* (Kifune & Maeta, 1978), **comb. nov.**; *Deltoxenosrueppelli* (Kinzelbach, 1971a), **comb. nov.**; *Xenosropalidiae* (Kinzelbach, 1975), **comb. nov.***Xenosminor* Kinzelbach, 1971a, **syn. nov.** is recognized as a junior synonym of *X.vesparum* Rossi, 1793. *Ophthalmochlusduryi* Pierce, 1908, **nomen nudum** and *Eupathoceralugubris* Pierce, 1908, **nomen nudum** are recognized as nomina nuda and therefore unavailable in zoological nomenclature. The species diversity of Xenidae probably remains poorly known: the expected number of species is at least twice as high as the number presently described.

## ﻿Introduction

Strepsiptera are a highly derived group of insect endoparasites and one of the smallest orders of holometabolous insects, comprising approximately 600 described species (Pohl and Beutel 2008; Cook 2019). Phylogenetic analyses of molecular data suggest an origin of Strepsiptera in the early Carboniferous (Toussaint et al. 2017; McKenna et al. 2019), even though the oldest fossils are known from Cretaceous Burmese amber (Pohl et al. 2020). The phylogenetic position of Strepsiptera was one of the most intractable enigmas in insect systematics (‘the Strepsiptera problem’, Kristensen 1981). Finally, a sister-group relationship with Coleoptera was convincingly confirmed by transcriptomic and genomic analyses (Boussau et al. 2014; Misof et al. 2014), and has been also supported by morphological data (Beutel et al. 2019).

Strepsipterans are obligate entomophagous parasites of species of seven insect orders (Zygentoma, Blattodea, Mantodea, Orthoptera, Hemiptera, Hymenoptera, and Diptera). Their morphology is strongly modified in all life stages and both sexes, which is clearly correlated with their highly specialized life cycle and endoparasitic habits. Strepsiptera undergo a dramatic hypermetamorphosis of body structures during development. Adult males and females are characterized by extreme sexual dimorphism (Pohl and Beutel 2008; Kathirithamby 2009). Conspicuous features of males are mesothoracic halteres, fan-shaped hind wings, specialized compound eyes (Buschbeck et al. 2003) with cornea lenses separated by chitinous bridges densely covered with microtrichia, and antler-shaped flabellate antennae (Ulrich 1930; Pohl and Beutel 2005). Adult males always leave the host and have an excellent flying capacity. In their very short life span of only few hours they must find a female and mate (Pix et al. 1993; Beani et al. 2005; Straka et al. 2011). Adult females are wingless, neotenic, and either free living (Mengenillidae and probably Bahiaxenidae) or permanently endoparasitic (remaining Strepsiptera: Stylopidia) (Pohl et al. 2018). They release a potent sex pheromone (Lagoutte et al. 2013; Zhai et al. 2016) to attract males for mating. Females produce numerous first-instar larvae viviparously. The miniaturized primary larvae, with an average length of ca. 230 µm (Pohl 2002), have three pairs of walking legs, an abdominal jumping device (with the exception of Stylopidae), and are very agile. They are well equipped with light sense organs and penetrate the body wall of the host using their mandibles (Pohl 2002; Pohl and Beutel 2008).

Xenidae and its sister taxon Stylopidae are groups with the highest degree of specialization in Strepsiptera. They belong to Stylopidia, a clade containing more than 97% of species of the order (Pohl and Beutel 2008). In contrast to Mengenillidae, which are restricted to Zygentoma as hosts, species of Stylopidia parasitize only pterygote insects. The dramatic change in life history linked with endoparasitic females caused far-reaching transformations of morphological characters (Pohl and Beutel 2008). Adult females of Stylopidia form a functional unit with the exuvia in contrast to the free-living wingless females of the family Mengenillidae (and probably Bahiaxenidae) (Kinzelbach 1971b, Pohl and Beutel 2005). The permanently endoparasitic females of Stylopidia are legless and extremely simplified morphologically. The anterior body regions form a compact sclerotized cephalothorax as a secondary tagma extruded from the host abdomen. The sack-shaped unsclerotized and unpigmented posterior body remains inside the host (Kinzelbach 1971b; Kathirithamby 1989).

The female cephalothorax in Xenidae and Stylopidae and all other groups of Stylopidia is in fact a product of fusion comprising the head, the thorax, and the anterior part of abdominal segment I (Löwe et al. 2016; Richter et al. 2017). This fusion of primary tagmata and segments increases the mechanical stability of the body part extruded from the host (Pohl and Beutel 2008). Likewise the flattening of the cephalothorax is interpreted as an adaptation to mechanical strain caused by the cuticle of the host’s abdominal segments (Kinzelbach 1971b). The distinct constriction in the middle region of abdominal segment I in Xenidae and Stylopidae marks the penetration point of the host’s body wall where the parasite is in direct contact with host intersegmantal membrane. It probably prevents the extruded anterior body part from slipping back into the body lumen of the host (Lauterbach 1954; Löwe et al. 2016; Richter et al. 2017).

The female cephalothoracic capsule includes the exuviae of the secondary and tertiary larval stages, forming a functional unit (puparium) with the female integument below these layers (Richter et al. 2017). The cephalothoracic part of the exuvia of secondary larvae is several times thicker than that of the tertiary stage. It is sclerotized and forms the main protective layer of the exposed part of the body (Richter et al. 2017). Many structures of cephalic and thoracic origin are distinctly or completely reduced, including the compound eyes, antennae, mouthparts, and legs, obviously correlated with endoparasitism (Kinzelbach 1971b; Pohl and Beutel 2005; Löwe et al. 2016; Richter et al. 2017). The spiracles on abdominal segment I are the only functional pair preserved in the females of Stylopidia. The absence of spiracles on segments II–VIII is very likely correlated with permanent endoparasitism (Pohl and Beutel 2005). Linked with the reduction of the primary female genital apparatus (e.g., ovaries and oviducts), novel structures involved in reproduction have evolved, such as a birth opening on the ventral side of the cephalothorax between the cephalic and prosternal regions. The birth opening is connected with birth organs by the brood canal. There, the copulation takes place and numerous first instar larvae are released (Kinzelbach 1971b, Kathirithamby 1989; Peinert et al. 2016).

The male puparium is similar to that of the female in some aspects, also involving the exuvia of the secondary larva, and also possessing a strongly sclerotized exposed anterior part and a large, distinctly less pigmented posterior region (Kinzelbach 1971b, Pohl and Beutel 2008). Ecdysial sutures are absent in the male puparium of Strepsiptera including the exuvia of the secondary larva. The anterior part of the puparium, the cephalotheca, is opened when the adult male leaves the host abdomen after finishing the development (Pohl and Beutel 2005). It is homologous to the head capsule of the secondary larva in the female cephalothorax. The cephalotheca is separated from the posterior part of the puparium by a circular furrow, a zone of weakness of the cuticle of the puparium. Kathirithamby (1990) described this structure as a preformed ecdysial line of weakness. To emerge, males of some genera (*Xenos*, *Stylops*) use their mandibles to open the cephalotheca, first piercing through it, and then cutting along a furrow in a scissor-like fashion (Grabert 1953; Kinzelbach 1971b, Hrabar et al. 2014). Once the cephalotheca is cut free, the male pushes it open with his head (Hrabar et al. 2014).

Xenidae originated relatively late, approximately 50–60 million years ago (McMahon et al. 2011). They are parasites of wasps from four families, viz. Hymenoptera: Aculeata: Crabronidae, Bembicidae, Sphecidae, and Vespidae (Benda et al. 2021). Xenidae are mainly distinguished from the closely related Stylopidae by the exclusive use of wasps as hosts (in contrast to bee hosts in Stylopidae) and unique characters of first instar larvae. The latter are adaptations to the smooth body surface of the hosts and enhance the attachment capacity. This includes enlarged and rounded adhesive tarsal pads and filamentous cuticular outgrowths of the labium which strongly increase the wettability (Pohl and Beutel 2004, 2008).

This group appeared in the literature as a subfamily “Xenides” inside the family Stylopidae in Saunders (1872) who made the first attempt to divide strepsipterans into taxonomic groupings and separating “Xenides” from “Pseudoxenides” (Cook 2019). Pierce (1908) was the first to use the name Xenidae as a family designation within the Strepsiptera. The taxonomic rank was changed by Kinzelbach (1971b) who treated Xeninae, Paraxeninae and Stylopinae as subfamilies of Stylopidae in a broader sense (Cook 2019). Pohl (2002) re-established Xenidae based on a cladistic analysis of morphological characters of the first instar larvae. He placed Xenidae as sister group of Stylopidae + Myrmecolacidae, rendering the Stylopidae in their former concept paraphyletic. Pohl and Beutel (2005), analyzing morphological characters of males, females and first instars, established Xenidae and Stylopidae as sister taxa, which was later supported by the molecular phylogeny of McMahon et al. (2011).

The first generic classification of Xenidae was provided by Pierce (1908, 1909, 1911) who described several genera based on a concept that each genus of Xenidae is specialized on one host genus of wasps. This concept was later rejected by Bohart (1941). A more recent classification of Xenidae has proposed four genera, each specialized on one or several families or subfamilies of hosts (Kinzelbach 1971b, Cook 2019). *Paragioxenos* Ogloblin is an enigmatic genus specialized on pollen wasps (Masarinae) with an endemic distribution in Australia. *Paraxenos* Saunders is distributed worldwide and specialized on wasps of the families Crabronidae, Sphecidae and Bembicidae. *Pseudoxenos* Saunders is also cosmopolitan and specialized on solitary potter wasps (Eumeninae). *Xenos* Rossi, which occurs on all continents except for Australia and Antarctica, parasitises social wasps of the subfamilies Polistinae and Vespinae. In clear contrast to this taxonomic concept, Benda et al. (2019) found little or no evidence for cophylogenetic links between strepsipteran parasites and hymenopteran host lineages, and refuted the monophyly of three of the traditional genera. These results were confirmed by a recent analysis with a denser taxon sampling, and it was suggested to re-evaluate the status of each genus in a more detailed taxonomic revision of the family, also based on morphology (Benda et al. 2021). Consequently, the main aim of the present study is a critical evaluation of the relationships and classification of the genera of Xenidae. Using various microscopic methods, we explore the morphology of the female cephalothorax and male cephalotheca. We compare our findings with results of previous molecular phylogenetic studies. Additionally, we provide a preliminary checklist of all described species of Xenidae. We also summarize host and distributional data for each described species. We understand this study as a first step towards a modern taxonomy of Xenidae. This should be crucial for a better understanding and easier investigation of these remarkable parasites in the future.

## ﻿Materials and methods

### ﻿Material

A total of 234 females and male puparia of Xenidae were obtained from hosts of the families Vespidae, Crabronidae, Bembicidae, and Sphecidae. Voucher names, hosts, and collection localities are listed in Suppl. material 1: Table S1. Material from the following public and private collections were examined:

**AMNH**American Museum of Natural History, New York, USA;

**CNC**Canadian National Collection of Insects, Arachnids, and Nematodes, Ottawa, Ontario, Canada;

**CUNHM**Chulalongkorn University Natural History Museum, Bangkok, Thailand;

**DBPC** Daniel Benda personal collection, Prague, Czech Republic;

**JSPC** Jakub Straka personal collection, Prague, Czech Republic;

**KUNHM** Natural History Museum, Division of Entomology, University of Kansas, Lawrence, Kansas, USA;

**NMPC**National Museum, Prague, Czech Republic;

**OLML**Oberösterreichisches Landesmuseum, Linz, Austria;

**YNPC** Yuta Nakase personal collection, Matsumoto, Japan.

### ﻿Fixation and preparation

All host individuals were first relaxed in water vapor and then immediately dissected. The endoparasitic females and males were removed from the host body. Females and male puparia used for morphological study were cleared using a mixture of lysis buffer ATL and proteinase K (Qiagen) heated to 56 °C. The lysis procedure took several hours or overnight. Cleared specimens were cleaned in distilled water several times and then stored in vials with 96% ethanol. Complete female cephalothoraces and male puparia were air-dried using a micro-pad inserted into the cephalothorax to prevent the cuticle from collapsing during the process. The female body was usually extracted from the cephalothorax before drying. After this step and the removal of the micro-pad, the dried specimens were glued onto card mounting points, which were pinned.

### ﻿Measurements

The width and length of the female cephalothorax, the female head capsule and the male cephalotheca were measured using a Leica S9D Stereomicroscope with a calibrated ocular micrometer. The cephalothorax length was measured from the apex of the clypeal lobe to the constriction of abdominal segment I; the cephalothorax width is the maximum distance between its lateral margins.

### ﻿Photomicrography

The general habitus of stylopized host specimens and the host abdomen with protruding strepsipterans were documented. Multifocus images were taken using Canon EOS 550D or 70D cameras equipped with EF 50 mm and MP-E 65 mm macro lenses. Lateral lights and a diffuser were used.

For the documentation of the original coloration of the female larval cephalothorax and the male cephalotheca, air-dried specimens glued to the card mounting points were used. They were photographed with a Canon EOS 7D digital SLR equipped with a Canon MP-E 65 mm macro lens (Canon, Krefeld, Germany) fitted with a StackShot macro rail (Cognisys, Traverse City, MI, USA). Each specimen was illuminated with two flashlights (Yongnuo Photographic Equipment, Shenzhen, China) fitted to a transparent cylinder for even and soft light. For the documentation of tiny structures on the head capsule, we used a Canon EOS 70D camera attached to an Olympus BX40 Microscope. The microscope was equipped with lateral lights and a diffuser. Zerene Stacker (Zerene Systems LLC, Richland, USA) was used to process stacks of images with different focus.

### ﻿Scanning electron microscopy (SEM)

Dried female cephalothoraces glued to card points were mounted on a rotatable specimen holder (Pohl 2010). Each specimen was sputter coated with gold with an Emitech K 500 (Sample preparation division, Quorum Technologies Ltd., Ashford, England). The SEM micrographs were taken with an ESEM XL30 (Philips, Amsterdam, Netherlands) equipped with Scandium FIVE (Olympus, Münster, Germany).

### ﻿Image processing

All images were processed and arranged into plates with Adobe Photoshop CS5 (Adobe System Incorporated, San Jose, USA) software. CorelDraw X8 (CorelDraw Corporation, Ottawa, ON, Canada) was used for the lettering of the plates.

### ﻿Terminology and description style

The terminology used for the female cephalothorax and male cephalotheca is based on Richter et al. (2017), Löwe et al. (2016), and Kinzelbach (1971b). Appropriate terminology was developed for morphological characters without specific names. In the diagnoses emphasis was placed on a distinction between apomorphic and plesiomorphic features within Xenidae in regard to the sister family Stylopidae. Cephalothoraces and cephalothecae were displayed in morphological orientation in figures although their functional orientation in the host body is inverted. Genera are listed in the order based on the phylogeny in Benda et al. (2021), species alphabetically.

## ﻿Results

### ﻿General description of the female cephalothorax of Xenidae

**Cephalothorax size.** Generally quite variable within species and depending on the host identity. Species with the smallest cephalothorax belong to the genera *Brasixenos* (smallest specimen: 0.76 mm long, 0.72 mm broad) and *Macroxenos* (0.84 mm long, 0.64 mm broad). The species with the maximum length are *Deltoxenos* sp. (2.83 mm long, 2.43 mm broad) and *Xenosmoutoni* Buysson (2.7 mm long, 2.43 mm broad), while the broadest cephalothorax was recorded for *Paraxenoshungaricus* (Székessy) (1.87 mm long, 2.57 mm broad).

**Cephalothorax shape.** Compact and ovoid, tapering anteriorly, usually longer than broad, but distinctly broader than long in several species (e.g., *Paraxenoshungaricus*); in cross-section it appears more or less flattened, elliptic, bent dorsad along its lateral margins (Richter et al. 2017).

**Cephalothorax coloration.** Variable, often pale, sometimes dark, or with multiple brown shades forming distinct patterns.

**Figure 1.**
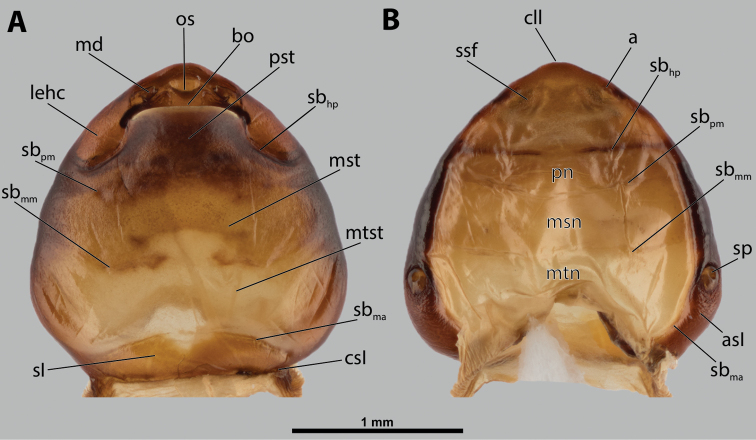
Deltoxenoscf.bequaerti, female, cephalothorax, photomicrographs **A** ventral side **B** dorsal side. Abbreviations: a – vestigial antenna, asI – abdominal segment I, bo – birth opening, cll – clypeal lobe, csI – constriction of abdominal segment I, lehc – lateral extension of head capsule, md – mandible, msn – mesonotum, mst – mesosternum, mtn – metanotum, mtst – metasternum, os – mouth opening, pn – pronotum, pst – prosternum (prosternal extension), sI – abdominal sternite I, sbhp – segmental border between head and prothorax, sbma – segmental border between metathorax and abdomen, sbmm – segmental border between mesothorax and metathorax, sbpm – segmental border between prothorax and mesothorax, sp – spiracle, ssf – supra-antennal sensillary field.

**Figure 2.**
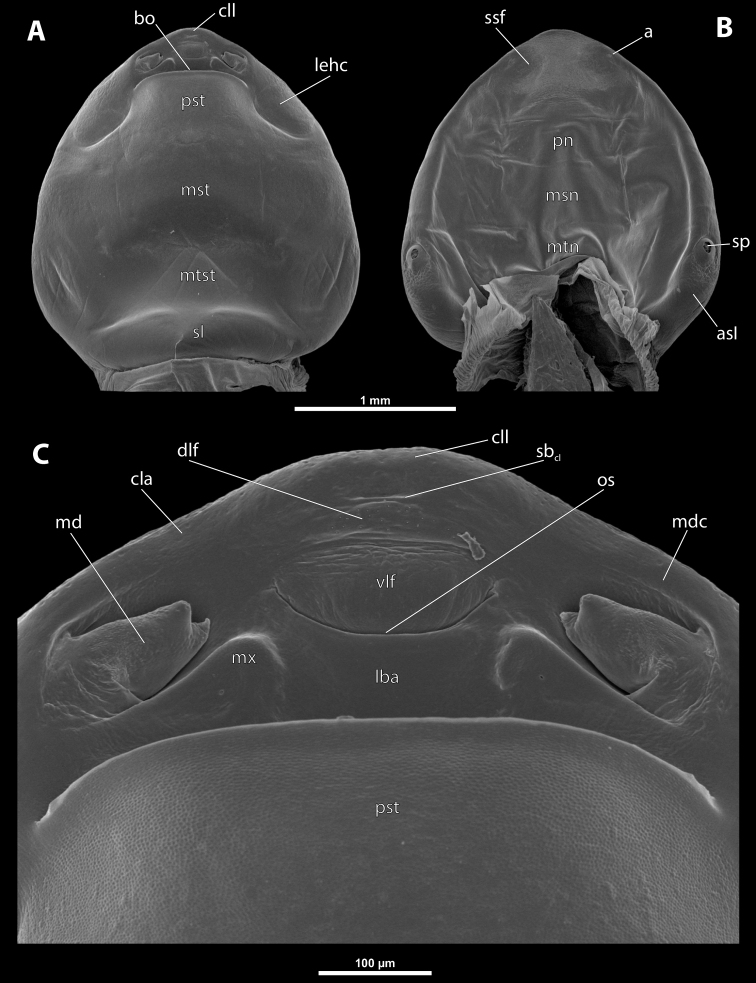
Deltoxenoscf.bequaerti, female, cephalothorax, SEM micrographs **A** ventral side **B** dorsal side **C** mouthparts and base of prosternum, ventral side. Abbreviations: a – vestigial antenna, asI – abdominal segment I, bo – birth opening, cla – clypeal area, cll – clypeal lobe, dlf – dorsal field of labral area, lba – labial area, lehc – lateral extension of head capsule, md – mandible, mdc – mandibular capsule (clypeal origin), msn – mesonotum, mst – mesosternum, mtn – metanotum, mtst – metasternum, mx – vestige of maxilla (maxilla), os – mouth opening, pn – pronotum, pst – prosternum (prosternal extension), sI – abdominal sternite I, sbcl – segmental border between clypeus and labrum, ssf – supra-antennal sensillary field, sp – spiracle, vlf – ventral field of labral area.

**Figure 3.**
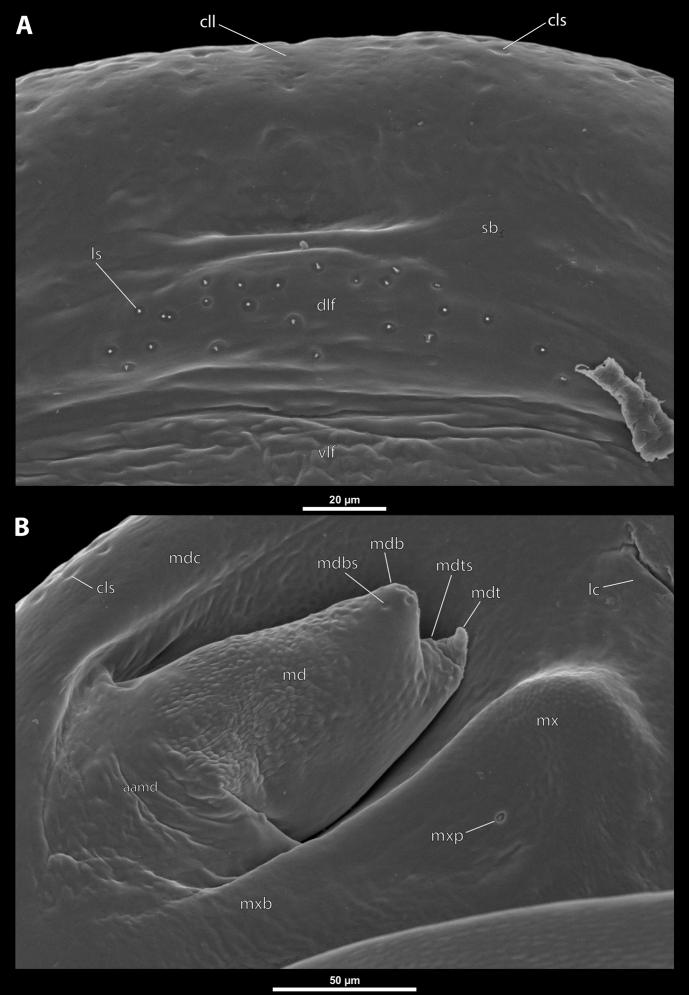
Deltoxenoscf.bequaerti, female, cephalothorax, SEM micrographs **A** clypeus and labrum, detail, ventral side **B** right mandible and maxilla, ventral side. Abbreviations: aamd – sclerotized mandibular membrane, cll – clypeal lobe, cls – clypeal sensillum, dlf – dorsal field of labral area, lc – labial corner, ls – labral seta in cavity (spine-shaped sensilla), md – mandible, mdb – mandibular bulge, mdbs – sensillum of mandibular bulge, mdc – mandibular capsule (clypeal origin), mdt – mandibular tooth, mdts – spine of mandibular tooth, mx – vestige of maxilla (maxilla), mxb – maxillary base (at mandible base), mxp – vestige of maxillary palp, sbcl – segmental border between clypeus and labrum, vlf – ventral field of labral area.

**Figure 4.**
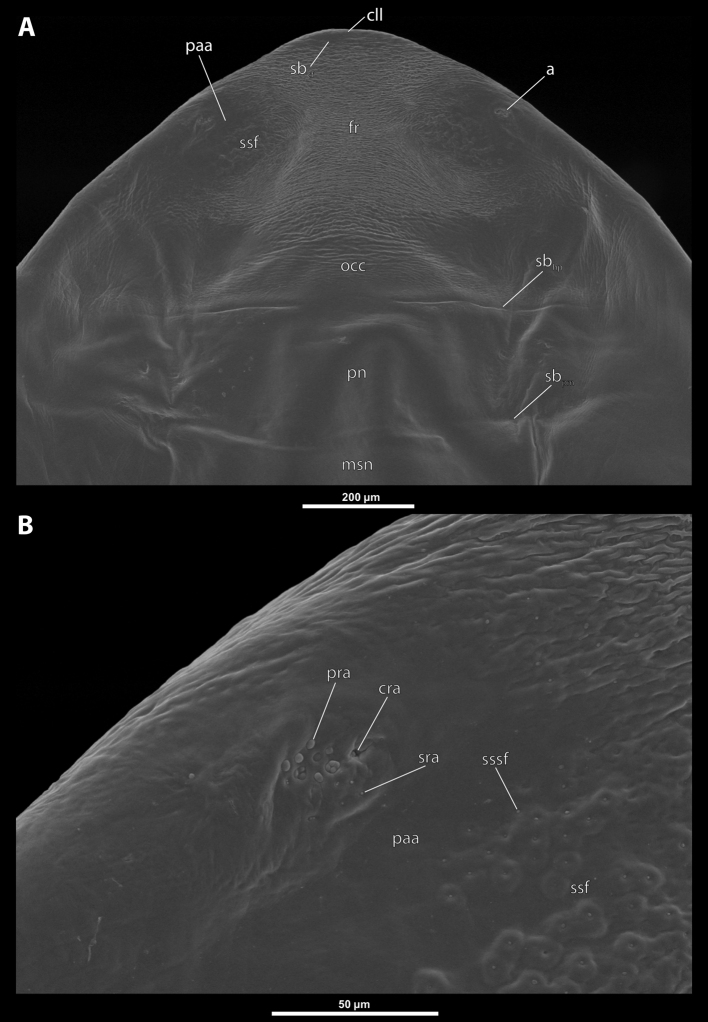
Deltoxenoscf.bequaerti, female, cephalothorax, SEM micrographs **A** anterior part of cephalothorax, dorsal side **B** vestigial antenna, dorsal side. Abbreviations: a – vestigial antenna, cll – clypeal lobe, cra – cavity of vestigial antenna, fr – frontal region, msn – mesonotum, occ – occipital area, paa – periantennal area, pn – pronotum, pra – plate of vestigial antenna, sbhp – segmental border between head and prothorax, sbpm – segmental border between prothorax and mesothorax, sra – sensillum of vestigial antenna, ssf – supra-antennal sensillary field, sssf – sensillum of supra-antennal sensillary field.

**Head capsule.** Prognathous, dorsoventrally more or less strongly flattened. Head length including lateral extensions of head capsule making up ~ ¼ ~ ½ the length of entire cephalothorax (Figs 1A, 2A). Posterior part almost completely fused to prothorax but still distinctly separated from it by birth opening (opening of the brood canal) medially, and by a suture laterally (Fig. 1A); completely separated by birth opening over the entire width of the ventral side only in *Paragioxenos* Ogloblin (Fig. 8A). Compound eyes and cephalic sutures missing. Labrum not present as a defined cephalic element. Clypeus poorly separated from frontal region, epistomal suture (frontoclypeal transverse strengthening ridge) missing, both cephalic areas thus fused; clypeal area tentatively marked by several sensilla; central part of clypeal area often forming a clypeal lobe; if present, then clypeal lobe well visible on head apex and protruding beyond the anterior edge of head capsule (Figs 1B, 2A, 2C); sensilla evenly dispersed over entire clypeal area or more concentrated on clypeal lobe (Fig. 3); lateral clypeal areas forming a mandibular capsule, also beset with sensilla (Fig. 3B). Frontal region not present as a delimited cephalic element, with variable microsculpture: smooth and shiny or rough, often forming reticulate structures or papillae (Fig. 25F). Border between head and thorax obsolete dorsally, but in some species with an interrupted suture and strongly pigmented (Figs 1B, 4A).

**Supra-antennal sensillary field.** Paired rounded areas, probably of frontal origin, present dorsomedially, with variable microsculpture and many sensilla, close to vestigial antennae (Fig. 4B).

**Antenna.** Vestigial, located dorsally on the head, close to the lateral margin, at the same level as maxillary vestige, either preserved as a groove, or as a cavity, or as a poorly defined area with several small, rounded plates and sensilla or setae (Fig. 4B); in some cases, antennal cavity also bearing plates and sensilla. Complete and distinct antennal torulus always missing, but incomplete vestigial torulus visible in some species. Periantennal area present close to vestigial antennae, lacking sensilla and defining the mesal border between antenna and supra-antennal sensillar field.

**Labrum.** Fused with head capsule, but still defined as oval area anterior to mouth opening; divided into dorsal labral field, likely corresponding with dorsal labral surface, and ventral labral field (Figs 2C, 3A), likely homologous to anterior epipharynx; dorsal field usually bearing several to many setae inserted in cavities, presumably of labral origin, varying in number from 10 to ~ 41; these setae cannot be clearly recognized in some cases. Lower margin of ventral field delimited by mouth opening; ventral field semicircular or oval shaped.

**Mandible.** Anteromedially directed, usually with hook-shaped apex directed anteriad, anteromesad, or anteroventrad; the angle varies between 20° and 75°. Anteriorly, mandibles partially enclosed by mandibular capsule, probably of clypeal origin (Fig. 3B). Anterior mandibular part bearing serrate tooth, directed distally and more or less covered with small spines; protuberant mandibular bulge sometimes present laterally, usually bearing several sensilla; cuticle of mandible variously sculptured, reticulate, covered by longitudinal grooves, or completely smooth. Laterally, mandible connected with head capsule by sclerotized mandibular membrane.

**Maxilla.** Highly variable, inserted posteromesad of mandibles; well-developed, reduced, or completely fused with labial area, placed ventromedially between mandibles (Fig. 2C); connected medially in some taxa. Maxillary base placed below mandible close to its articulatory area (Fig. 3B); anterior maxillary region reaching beyond mandibular tip in some species. Maxillary endite lobes and well-defined maxillary palp missing; variously placed concavity likely representing a rudiment of the latter. Maxillary surface smooth or sculptured, for instance reticulate. Maxillary bases usually continuous with submaxillary groove, which is not part of maxilla; adjacent to border between head and prothorax. In species with a distinctly produced submaxillary groove, this area is visible between the submaxillary groove and the ventrolateral cephalo-prothoracic suture (Figs 18A, 34A, 37A).

**Labium and hypopharynx.** Labium not recognizable as a separate structure, probably fused to anteroventral cephalic capsule; the well-delimited area between maxillae is probably of labial origin, anteriorly delimited by the mouth opening and posteriorly by the birth opening (Fig. 2C). Labial area raised anteriorly in some taxa as a small spine projecting beyond the mouth opening, or laterally as paired labial corners (Fig. 3B). Hypopharynx absent or rarely present as inconspicuous protuberance.

**Mouth opening.** Present as narrow transverse cleft between mandibles, maxillae, and labium (Fig. 2C); semicircular to shallowly U-shaped, sometimes arcuate or bi-arcuate; usually sclerotized marginally, mainly on the labial side.

**Salivarium.** Not developed.

**Birth opening.** Present as narrow cleft on ventral side of cephalothorax, indicating border between head and prothorax (Figs 1A, 2A); usually continuous with a suture posterolaterally, but extending over the entire width of the ventral side in *Paragioxenos* (Fig. 8A). In virgin females, the birth opening is closed by larval cuticle (brood canal membrane, Fig. 47C), which is very thin there, translucent, and nearly invisible under an optical microscope (Fig. 45C); remnants of ruptured membrane visible in mated females (Fig. 14C).

**Thorax and abdominal segment I.** Three thoracic segments completely fused with each other and also with abdominal segment I. Cephalothorax broadest at level of abdominal spiracles I. Thoracic segmental borders and thoraco-abdominal border distinct to different degrees, well visible, in distinct to almost completely invisible; segmental borders less distinct dorsally; in many cases only some of them visible (differentiation of thoracic segments varies even within species, not only between species and genera). Thoracic segments usually separated by mesal furrows combined with pigmented stripes or spots (Fig. 1A, 1B); pigmented areas sometimes without furrows and with changed cuticular sculpture. Cuticle on ventral side of thoracic segments displaying reticulate pattern, with scattered inconspicuous or more distinct pigmented papillae usually forming specific pattern (Figs 10A, 28C); cuticular surface on thorax dorsally smooth or slightly wrinkled. Border between metathorax and abdomen usually indicated by an edge, color change, or change of cuticular microsculpture (Fig. 1A). Prothorax with prosternal extension reaching towards head capsule (Fig. 1A). Cephalothoracic tergites, pleurites and sternites fused. Legs missing. Transverse medial constriction of abdominal segment I in direct contact with host intersegmental membrane, forming posterior border of cephalothorax (Fig. 1A); posterior part of abdominal segment I and remaining abdominal segments located in body cavity of host. Spiracles of abdominal segment I functional; setae, and cuticular spines present on this segment laterally, below spiracles (Figs 17E, 21E); this area is distinctly wrinkled in some species (Fig. 13E) and sometimes extruding as spiracular corner (Fig. 28D).

**Spiracles.** Paired, annular or semicircular, located laterally or dorsolaterally on posterior most part of cephalothorax; surrounding cuticle forming distinct ring-shaped microstructure but only slightly elevated (Figs 1B, 2B). Spiracle orientation variable, but in most species anterolateral.

### ﻿General description of the cephalotheca of the male puparium in Xenidae

**Cephalotheca shape.** Rounded to elliptic in frontal view; always broader than long, distinctly flattened or almost circular in cross section; rounded or pointed apically in lateral view.

**Figure 5.**
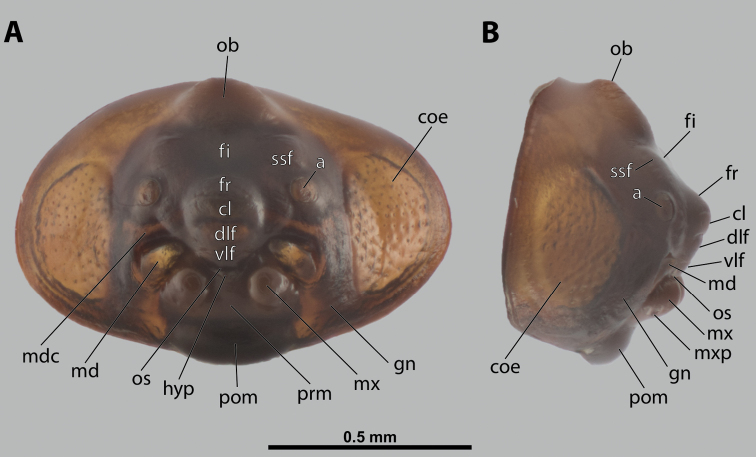
Deltoxenoscf.bequaerti, male, cephalotheca, photomicrographs **A** frontal view **B** lateral view. Abbreviations: a – vestigial antenna, cl – clypeus, coe – compound eye, dlf – dorsal field of labral area, fi – frontal impression, fr – frontal region, gn – gena, hyp – hypopharynx, md – mandible, mdc – mandibular capsule (clypeal origin), mx – vestige of maxilla (maxilla), ob – occipital bulge, os – mouth opening, pom – postmentum, prm – praementum, ssf – supra-antennal sensillary field, vlf – ventral labral field of area.

**Figure 6.**
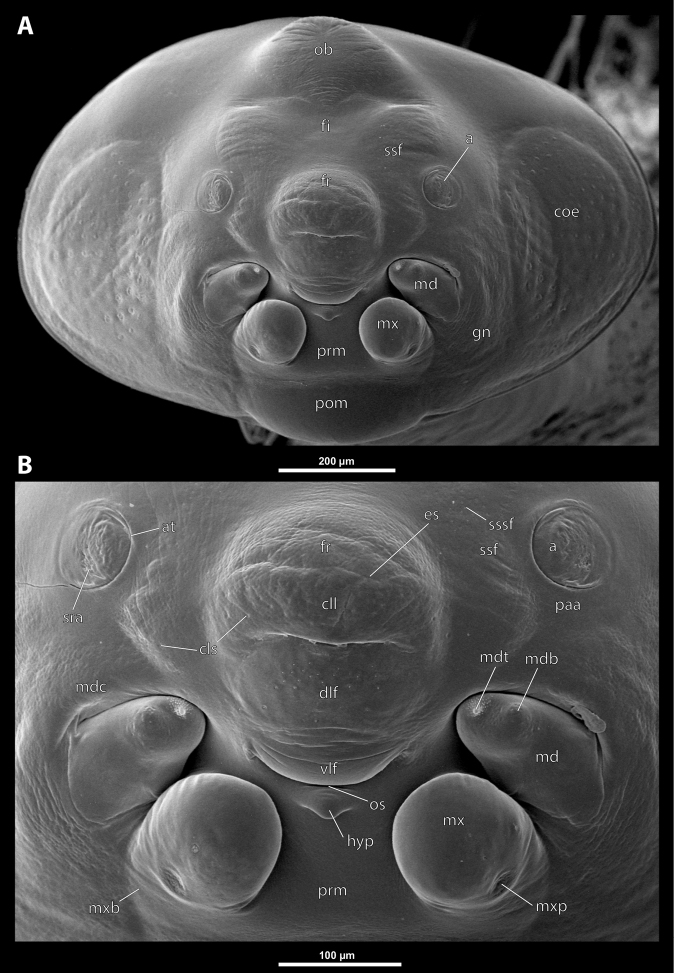
Deltoxenoscf.bequaerti, male, cephalotheca, SEM micrographs **A** frontal view **B** mouthparts. Abbreviations: a – vestigial antenna, at – antennal torulus (rudiments of antennal torulus), cll – clypeal lobe, cls – clypeal sensillum, coe – compound eye, dlf – dorsal field of labral area, es – epistomal suture, fi – frontal impression, fr – frontal region, gn – gena, hyp – hypopharynxgeal protuberance, md – mandible, mdb – mandibular bulge, mdc – mandibular capsule (clypeal origin), mdt – mandibular tooth, mx – vestige of maxilla (maxilla), mxb – maxillary base (at mandible base), ob – occipital bulge, os – mouth opening, paa – periantennal area, pom – postmentum, prm – praementum, sra – sensillum of vestigial antenna, ssf – supra-antennal sensillary field, sssf – sensillum of supra-antennal sensillary field, vlf – ventral field of labral area.

**Cephalothecal capsule.** Compound eyes present (Figs 5A, 6A); individual ommatidia usually visible as dark sclerotized impressions on pale background of ocular area except for some *Xenos* spp. with ocular area completely dark. Clypeus (**cl**) well developed, flattened, and elongated, with epistomal suture separating it from frontal region; shape variable, more or less curved or nearly straight, usually medially protruding from cephalotheca as clypeal lobe. Clypeal sensilla (Fig. 6B) distributed over entire surface, evenly dispersed, or mainly concentrated on clypeal lobe medially. Lateral clypeal portions forming mandibular capsule. Frontal region well-delimited against clypeus, usually with frontal impression or furrows. Genal regions visible but not clearly delimited. Occipital bulge more or less distinctly developed or absent; usually with coarser microsculpture (Fig. 5).

**Supra-antennal sensillary field.** Paired kidney-shaped and bulging supra-antennal sensillary fields, probably of frontal origin, located mesad of vestigial antennae; with numerous sensilla; on its mesal side often delimited by a more or less distinct furrow (Figs 15A, D, 19A, D) which also delimits the mesal part of the frontal region connected with the clypeal lobe.

**Antenna.** Vestigial, inserted between compound eye and supra-antennal sensillary field; rounded and blunt; surrounding area well-defined, equipped with sensilla and delimited by a distinct antennal torulus (Fig. 6B), which is interrupted in some cases. A periantennal area is present close to the vestigial antennae; it lacks sensilla and separates the antenna from supra-antennal sensillary field mesally.

**Labrum.** Fused with head capsule, but still defined as oval area anterior to mouth opening; divided into dorsal and ventral labral fields (Figs 5A, B, 6B), the former equipped with variable number of setae inserted in cavities. Dorsal field likely homologous with upper labral surface, ventral field with anterior epipharynx.

**Mandible.** Directed anteromesally, enclosed by mandibular capsule located anterolaterally (Fig. 6B); with small, anteriorly directed serrate tooth anteromesally, bearing dense field of minute spines. A protuberant mandibular bulge present anterolaterally, usually bearing several sensilla.

**Maxilla.** Inserted posteromesad of mandibles, well-developed as separate structures or completely fused with labial area, which is medially enclosed between the maxillae (Fig. 6B). Vestigial maxillary palp present on maxillary base.

**Labium and hypopharynx.** Labium distinctly recognizable between and below maxillae, usually clearly subdivided into praementum and postmentum (Figs 5A, 6A). Small median external protuberance (Fig. 6B), possibly homologous with the distal hypopharyngeal region, often present below mouth opening.

**Mouth opening.** Present as narrow transverse cleft between mandibles and maxillae (Figs 5A, 6B), semicircular to U-shaped, and covered by ventral labral field in some taxa.

**Salivarium.** Not developed.

## ﻿Review of genera of Xenidae

### ﻿ Paragioxenos

Taxon classificationAnimaliaStrepsipteraXenidae

Ogloblin, 1923

C1ECE3D2-24E8-56FA-9E3B-1F8262421D3F

Paragioxenos Ogloblin, 1923: 46. Type species: Paragioxenosbrachypterus Ogloblin, 1923, by original designation.

#### Diagnosis of female cephalothorax.

Differing from other Xenidae in following characters. Head and prothorax completely separated by birth opening on ventral side (Fig. 8A). Mandibles distinctly protruding from mandibular capsule; angle of mandibles 75°. Dorsal labral field elliptic, ~ 2× wider than long in midline, distinctly protuberant, straight (Fig. 8A). Conspicuous swelling present on prosternum (Fig. 8A), similar to some *Paraxenos* spp.

#### Description of female cephalothorax.

**Figure 7.**
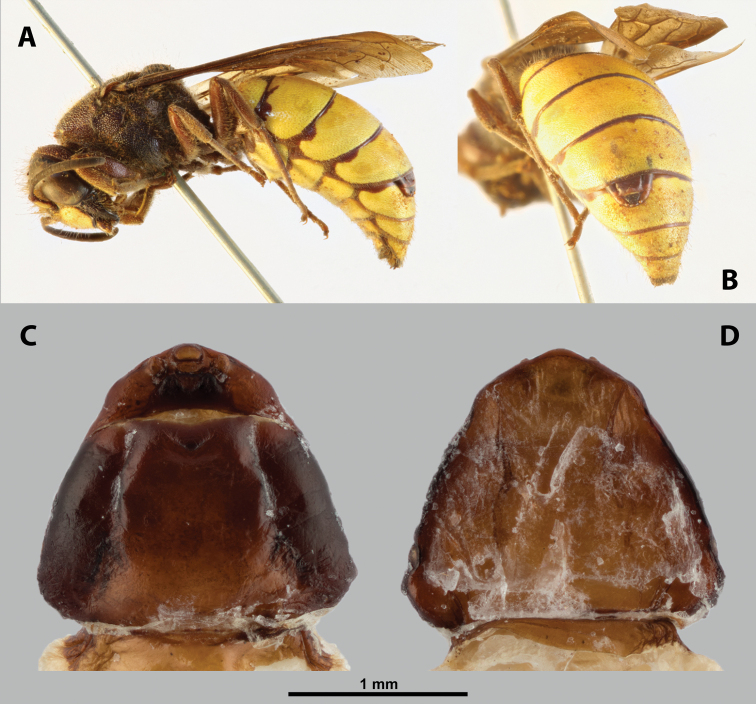
*Paragioxenosbrachypterus* Ogloblin, host, female, cephalothorax, photomicrographs **A**Paragiacf.decipiens Shuckard stylopized by female of *P.brachypterus*, lateral view **B** detail of host abdomen with adult female inside **C** ventral side of cephalothorax **D** dorsal side of cephalothorax.

**Shape and coloration.** Nearly triangular, slightly wider than long, length 1.68 mm, width 1.82 mm. Anterior cephalic margin very slightly protruding anteriorly. Thorax distinctly widening posteriorly. Coloration comprising multiple brown shades forming distinct pattern, mostly dark (Fig. 7C, D).

**Figure 8.**
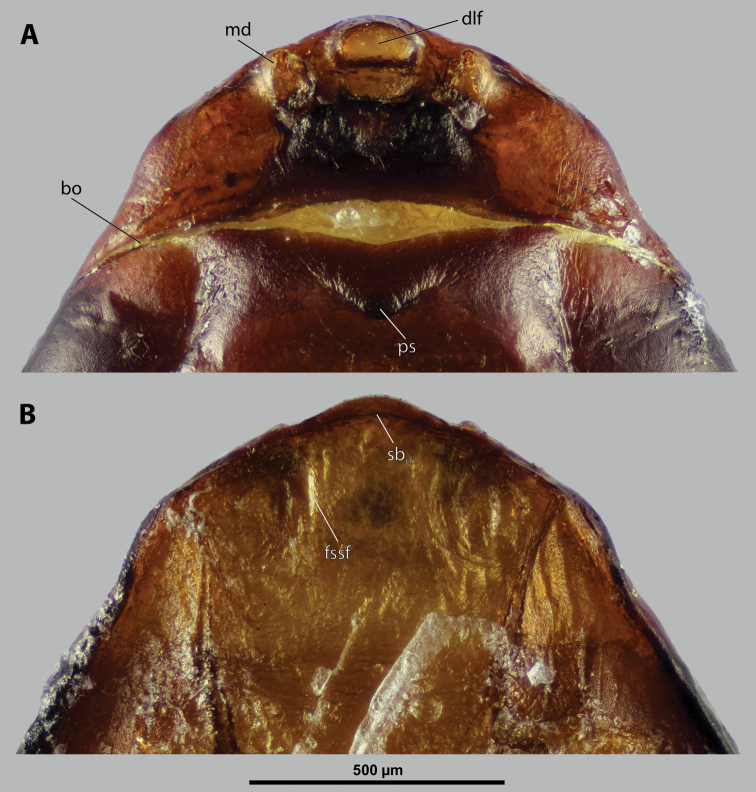
*Paragioxenosbrachypterus* Ogloblin, anterior part of female cephalothorax, photomicrographs **A** anterior part of cephalothorax, ventral side **B** Anterior part of cephalothorax, dorsal side. Abbreviations: bo – birth opening, dlf – dorsal field of labral area, fssf – furrow of supra-antennal sensillary field, md – mandible, ps – prosternal swelling, sbcf – segmental border between clypeus and frontal region.

**Head capsule.** Approximately ⅓ as long as entire cephalothorax including lateral cephalic extensions. Coloration mostly brown, including sclerotized labial area and strongly sclerotized mandible; dorsal labral field pale. Clypeal and labral area separated, the former slightly protruding anteriorly, forming inconspicuous clypeal lobe; surface of clypeal area slightly wrinkled; sensilla present. Border between clypeal and frontal regions quite indistinct. Cuticle of frontal region slightly wrinkled. Segmental border between head and prothorax indistinct dorsally; on ventral side completely separated by birth opening (Fig. 8A).

**Supra-antennal sensillary field.** More or less distinctly delimited by furrow on mesal side (Fig. 8B).

**Antenna.** Presence or absence of vestige of antennae not verified.

**Labrum.** Ventral labral field elliptic, not protruding; dorsal field elliptic, ~ 2× wider than long in midline, distinctly protuberant, straight (Fig. 8A). Presence or absence of setae not verified.

**Mandible.** Anteroventrally directed, distinctly protruding from mandibular capsule, nearly reaching or projecting slightly beyond anterior edge of head (Fig. 8A). Mandibular bulge distinctly raised, with sensilla. Mandibular tooth conspicuous.

**Maxilla.** Anteriorly directed, distinctly prominent, strongly sclerotized. Bases wide, connected in midline. Apical portion not projecting beyond mandible. Presence or absence of vestige of palp not verified. Submaxillary groove absent.

**Labium.** Triangular, sclerotized, and flat, located between maxillae, delimited anteriorly by mouth opening and posteriorly by connected maxillae.

**Mouth opening.** Fissure-shaped, straight medially, curved laterally, with sclerotized margin.

**Thorax and abdominal segment I.** Two longitudinal ventral furrows present mesally over whole length of thorax, slightly widening posteriorly. Pro-mesothoracic and meso-metathoracic borders indistinct. Border between metathorax and abdomen formed by ridge on dorsal side, indistinct on ventral side. Cuticle of thoracic segments dark laterally, less pigmented mesally between longitudinal furrows. Dorsal surface mostly with uniformly brown coloration except for lateral most region. Prosternum with pointed swelling but lacking extension (Fig. 8A). Setae and cuticular spines on lateral parts of abdominal segment I not examined.

**Spiracles.** Situated on posterior third of cephalothorax, slightly elevated, with anterolateral orientation.

#### Diagnosis of male cephalotheca.

No male cephalotheca was examined (absent in Ogloblin’s type material in NMPC).

#### Phylogenetic relationships.

Unknown.

#### Diversity and distribution.

Monotypic, restricted to Australia.

#### Host.

*Paragia* spp. (Vespidae: Masarinae).

### ﻿List of species

#### ﻿ Paragioxenos brachypterus

Taxon classificationAnimaliaStrepsipteraXenidae

Ogloblin, 1923

DA4840AB-6B5F-583F-9EB1-F9CDCA432A2D

Paragioxenos brachypterus Ogloblin, 1923: 46.

##### Hosts.

Paragiacf.decipiens Shuckard, 1837 (Ogloblin 1923); *Paragiadecipiens* Shuckard, 1837; *Paragiatricolor* Smith, 1850 (Hofeneder 1928).

##### Distribution.

South Australia: Gawler (Ogloblin 1923; Hofeneder 1928).

#### ﻿ Nipponoxenos

Taxon classificationAnimaliaStrepsipteraXenidae

Kifune & Maeta, 1975, stat. res.

70DA113D-322F-5822-B31F-6DF9C946D6B8

Nipponoxenos Kifune & Maeta, 1975: 446 (as a subgenus of Xenos Rossi). Type species: Xenos (Nipponoxenos) vespularum Kifune & Maeta, 1975, by original designation.

##### Diagnosis of female cephalothorax.

Differing from most genera in following combination of characters. Mandibles protruding distinctly from mandibular capsule, reaching or slightly projecting beyond cephalic edge (Fig. 10A). Maxilla anteriorly directed, strongly sclerotized. Maxillary bases conspicuously wide, connected in midline along birth opening. Anterior part of maxilla pointed (Fig. 10A). In contrast to *Paragioxenos*, head and prothorax ventrally delimited by birth opening medially and by suture laterally. Cephalothorax mostly pale.

##### Description of female cephalothorax.

**Figure 9.**
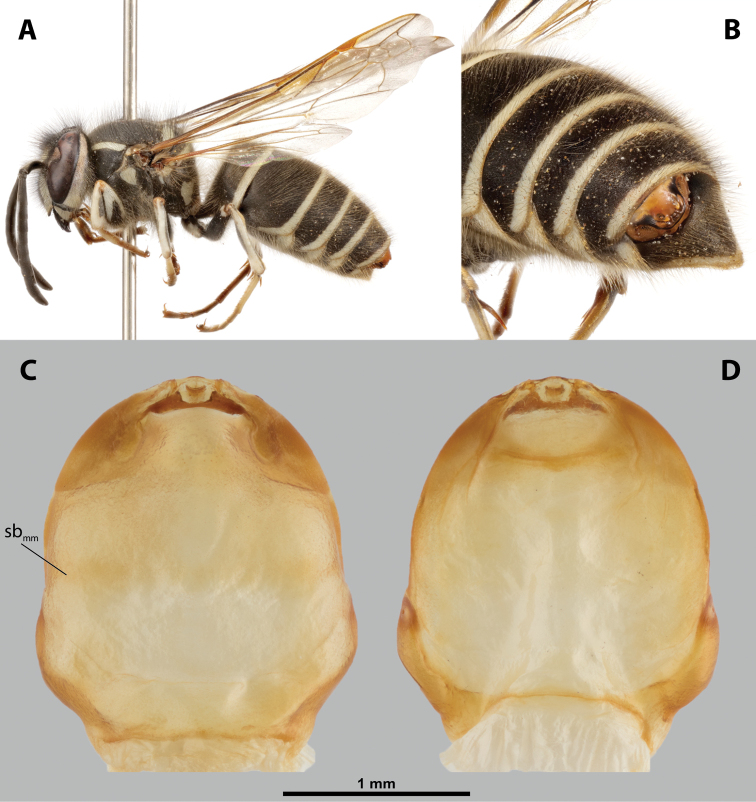
*Nipponoxenosvespularum* Kifune & Maeta, host, male, female, cephalothorax, photomicrographs **A***Vespulashidai* Ishikawa, Sk. Yamanne & Wagner stylopized by male of *N.vespularum*, lateral view **B** detail of host abdomen with male puparium inside **C** ventral side of female cephalothorax **D** dorsal side of female cephalothorax. Abbreviation: sbmm – segmental border between mesothorax and metathorax.

**Shape and coloration.** Cephalothorax distinctly longer than wide, length 2.0 mm, maximum width 1.76 mm. Anterior head margin not protruding. Thorax nearly straight. Meso-metathoracic border slightly constricted (Fig. 9C). Coloration with distinct pattern of different pale brown shades; usually medially pale and slighter darker laterally in ventral and dorsal view.

**Figure 10.**
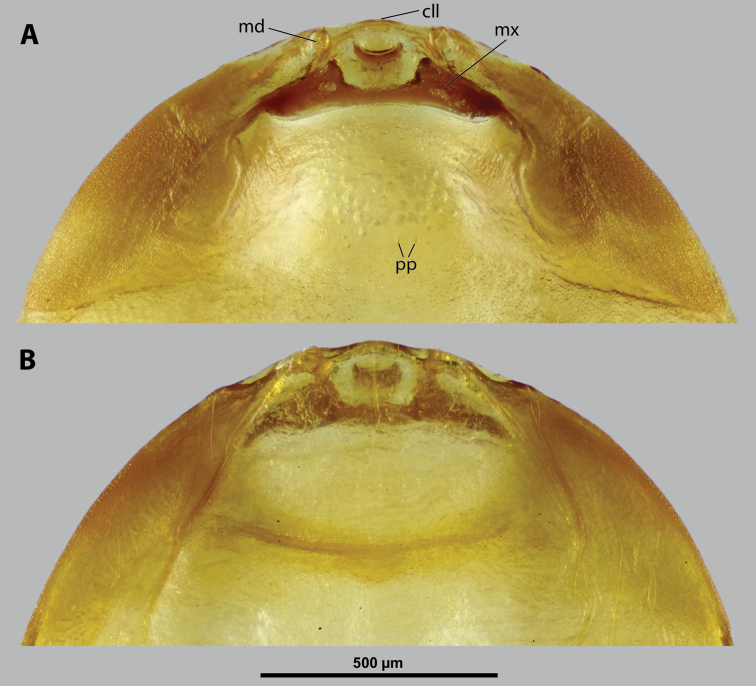
*Nipponoxenosvespularum* Kifune & Maeta, anterior part of female cephalothorax, photomicrographs **A** anterior part of cephalothorax, ventral side **B** Anterior part of cephalothorax, dorsal side. Abbreviations: cll – clypeal lobe, md – mandible, mx – vestige of maxilla (maxilla), pp – pigmented papillae.

**Head capsule.** Almost ⅓ as long as entire cephalothorax including lateral cephalic extensions. Coloration mostly pale brown, but darker on lateral extensions and on distinctly sclerotized maxillae (Fig. 10A). Clypeal area delimited from labral area, slightly protruding anteriorly, forming inconspicuous, slightly pigmented clypeal lobe (Fig. 10A); clypeal sensilla present. Border between clypeal and frontal region distinct. Cuticle of frontal region slightly wrinkled. Segmental border between head and prothorax indistinct dorsally but indicated by coloration; on ventral side separated by birth opening medially and by suture laterally.

**Supra-antennal sensillary field.** Not delimited by furrow mesally.

**Antenna.** Presence or absence of antennal vestige not verified.

**Labrum.** Ventral labral field elliptic, not protruding but slightly convex. Dorsal labral field elliptic, ~ 5× wider than long, slightly arcuate. Presence or absence of labral sensilla not verified.

**Mandible.** Anteromedially directed at angle of 60°, distinctly protruding from mandibular capsule, reaching or slightly projecting beyond anterior edge of head (Fig. 10A). Bulge not distinctly raised. Sensilla not examined. Mandibular tooth narrow or moderately widened, pointed apically.

**Maxilla.** Anteriorly directed, pointed, strongly sclerotized. Bases wide, connected medially. Apical region not projecting beyond mandible anteriorly. Presence of palp vestige not verified. Submaxillary groove slightly produced.

**Labium.** Labial area inserted between maxillae, slightly pigmented medially; anteriorly delimited by mouth opening and posteriorly by connected maxillary bases.

**Mouth opening.** Mouth opening slightly curved, sclerotized along margin.

**Thorax and abdominal segment I.** Pro-mesothoracic and meso-metathoracic borders vaguely indicated ventrally by pigmented stripes with specific cuticular surface, but nor recognizable on dorsal side (Fig. 9C, D). Border between metathorax and abdomen marked by ridge and change of cuticular sculpture and pigmentation. Entire abdominal segment I darker than thorax. Cuticle of thoracic segments on ventral side wrinkled or reticulate, with several small, pigmented papillae on prothorax. Prosternal extension undifferentiated, evenly arched. Dorsal side of thorax mostly smooth. Meso- and metathorax unmodified in shape, transverse. Setae and cuticular spines on lateral region of abdominal segment I not examined.

**Spiracles.** Situated on posterior ⅓ of cephalothorax, slightly elevated, with anterolateral orientation.

##### Diagnosis of male cephalotheca.

**Figure 11.**
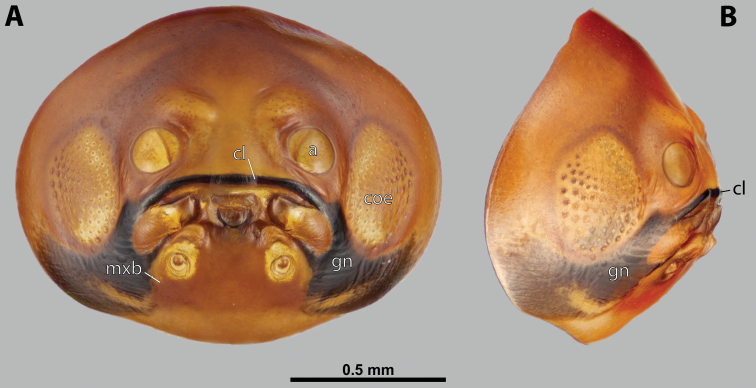
*Nipponoxenosvespularum* Kifune & Maeta, male, cephalotheca, photomicrographs **A** frontal view **B** lateral view. Abbreviations: a – vestigial antenna, cl – clypeus, coe – compound eye, gn – gena, mxb – maxillary base.

Less pigmented than in other genera of Xenidae. With conspicuous, nearly black clypeus and very short and black genae, very distinct on lightly colored surrounding areas of cephalotheca (Fig. 11). Antennal vestige very large (Fig. 11A).

##### Description of male cephalotheca.

**Shape and coloration.** Rounded laterally in frontal view, widely elliptic (Fig. 11A); rounded in lateral view (Fig. 11B). Coloration pale except for clypeus and genae (Fig. 11).

Cephalothecal capsule. Compound eyes with individual ommatidia well visible. Clypeus black colored; inconspicuous clypeal lobe straight in frontal view; sensilla mainly concentrated on clypeal lobe and on lateral parts of clypeus. Frontal region not deformed, lacking frontal impression. Occipital bulge rather indistinct. Diameter of genae (black) between maxillary base and compound eye very small, subequal to antennal diameter (Fig. 11A). Occipital bulge absent.

**Supra-antennal sensillary field.** Kidney-shaped and bulging, delimited medially by quite indistinct furrow.

**Antenna.** Antennal vestige very large, with complete torulus. Periantennal area distinctly delimited.

**Labrum.** Labral area distinct. Setae of dorsal field present.

**Mandible.** Anteromedially directed. Coloration darker anteriorly and less pigmented posteriorly. Bulge pointed.

**Maxilla.** Distinct, prominent. Coloration darker anteriorly, posterior part around vestige of palp less pigmented.

**Labium and hypopharynx.** Located between and below maxillae. Praementum and postmentum distinct, separated by slightly paler coloration of postmentum. Hypopharyngeal protuberance inconspicuous.

**Mouth opening.** Mouth opening distinctly arcuate, nearly U-shaped.

##### Phylogenetic relationships.

One of the earliest diverging lineages of Xenidae with a Palearctic origin (Benda et al. 2019). Placed either as sister to *Tachytixenos* Pierce + *Paraxenos* Saunders or as the earliest diverging group, sister to all other Xenidae (Benda et al. 2021).

##### Diversity and distribution.

Monotypic, restricted to East Asia.

##### Hosts.

*Vespula* spp. (Vespidae: Vespinae).

##### Comments.

The monotypic *Nipponoxenos* was originally described as a subgenus of *Xenos* by Kifune and Maeta (1975). We classify it as a valid genus, based on a molecular phylogeny (Benda et al. 2019) and morphological characters newly reported here.

### ﻿List of species

#### ﻿ Nipponoxenos vespularum

Taxon classificationAnimaliaStrepsipteraXenidae

Kifune & Maeta, 1975

0B8236B6-3A26-5EEA-BADD-2178AD50393B

Xenos (Nipponoxenos) vespularum Kifune & Maeta, 1975: 447.

##### Hosts.

*Vespulaflaviceps* (Smith, 1870) (as *Vespulalewisi* Cameron, 1903) (Kifune and Maeta 1975); *Vespulaflavicepsflaviceps* (Smith, 1870) (Kifune and Yamane 1991), *Vespulashidai* Ishikawa, Sk. Yamanne & Wagner, 1980 (Nakase and Kato 2013).

##### Distribution.

Japan: Honshu; Russia: Primorskij Kraj, Ussurijsk (Kifune and Yamane 1991).

##### Note.

This species was described under the monotypic subgenus NipponoxenosKifune and Maeta 1975.

#### ﻿ Tachytixenos

Taxon classificationAnimaliaStrepsipteraXenidae

Pierce, 1911, stat. res.

1AC6FE80-0F7C-5431-85C2-77A14EFA87BB

Tachytixenos Pierce, 1911: 501. Type species: Tachytixenosindicus Pierce, 1911, by original designation.
Pseudoxenos Saunders, 1872 (partim!) (synonymy proposed by Hofeneder 1949: 148).
Paraxenos Saunders, 1872 (partim!) (synonymy proposed by Kinzelbach 1971b: 162).

##### Diagnosis of female cephalothorax.

Differing from the other genera by a specific shape of the mandibular tooth, which is very wide basally and reaches the area of mandibular bulge. Tooth with pointed, ventrally directed apex. Base of tooth ventrally covered with small depressions continuous with several rows of spines (Fig. 14E). Prosternal extension undifferentiated (compared to similar genus *Paraxenos*), evenly arched, without any swelling or color differentiation. Maxillae distinctly prominent as in *Pseudoxenos*, *Tuberoxenos*, and some *Paraxenos* species. Mandible not protruding from capsule. In contrast to *Paragioxenos*, head and prothorax ventrally delimited by birth opening medially and by suture laterally.

##### Description of female cephalothorax.

**Figure 12.**
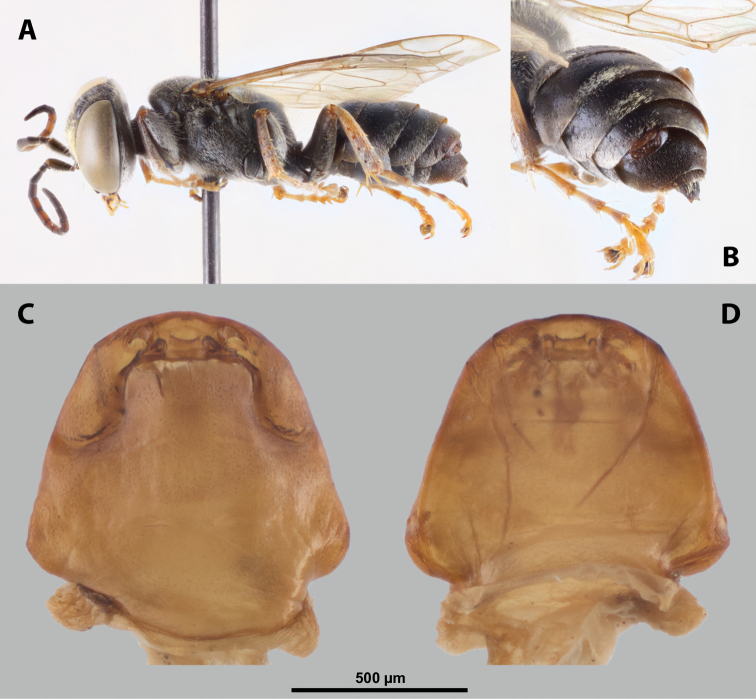
Tachytixenoscf.indicus Pierce, host, female, cephalothorax, photomicrographs **A***Tachytes* sp. stylopized by female of T.cf.indicus, lateral view **B** detail of host abdomen with adult female inside **C** ventral side of cephalothorax **D** dorsal side of cephalothorax.

**Shape and coloration.** Cephalothorax compact, ca. as long as wide, or slightly wider than long, or vice versa. Size varying strongly within genus, length 0.94–1.82 mm, width 0.88–1.88 mm. Anterior head margin evenly rounded or projecting. Thorax slightly widening posteriorly. Coloration comprising multiple brown shades and distinct patterns (Fig. 12C, D).

**Head capsule.** Approximately ¼ ~ ½ as long as entire cephalothorax including lateral extensions. Coloration variable, pale, completely dark brown, or forming specific color pattern. Clypeal area well delimited from labral area, arcuate, or slightly protruding anteriorly forming clypeal lobe. Surface of clypeal area smooth or slightly wrinkled. Sensilla (~ 40–55) regularly dispersed over clypeal surface or mainly concentrated on clypeal lobe. Border between clypeal and frontal region present but indistinct. Frontal region smooth or slightly wrinkled. Dorsal segmental border between head and prothorax distinct or only recognizable.

**Figure 13.**
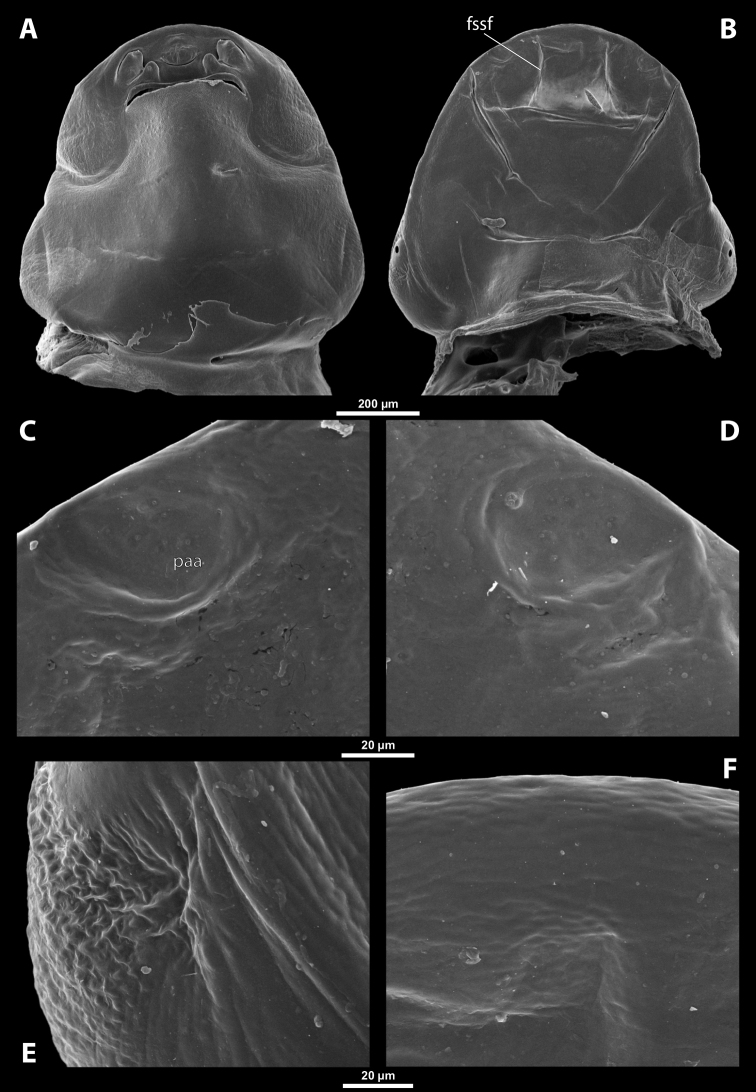
Tachytixenoscf.indicus Pierce, female, cephalothorax, SEM micrographs **A** ventral side **B** dorsal side **C** left vestigial antenna, dorsal side **D** right vestigial antenna, dorsal side **E** left lateral border of abdominal segment I below spiracle, dorsal side **F** detail of anterior border of cephalothorax, dorsal side. Abbreviations: fssf – furrow of supra-antennal sensillary field, paa – periantennal area.

**Supra-antennal sensillary field.** Smooth with dispersed sensilla, delimited by distinct furrow on medial side (Fig. 13B).

**Antenna.** Preserved as poorly defined area with several minute rounded plates, antennal sensilla, or cavity, in some cases all three combined. Periantennal area smooth, flat, or forming incomplete elliptic wall between antenna and supra-antennal sensillary field (Fig. 13C, D).

**Labrum.** Ventral field wider than long, elliptic. Dorsal field slightly arcuate, at least 3× wider than long in midline. Dorsal field bearing ~ 15–30 setae inserted in cavities.

**Figure 14.**
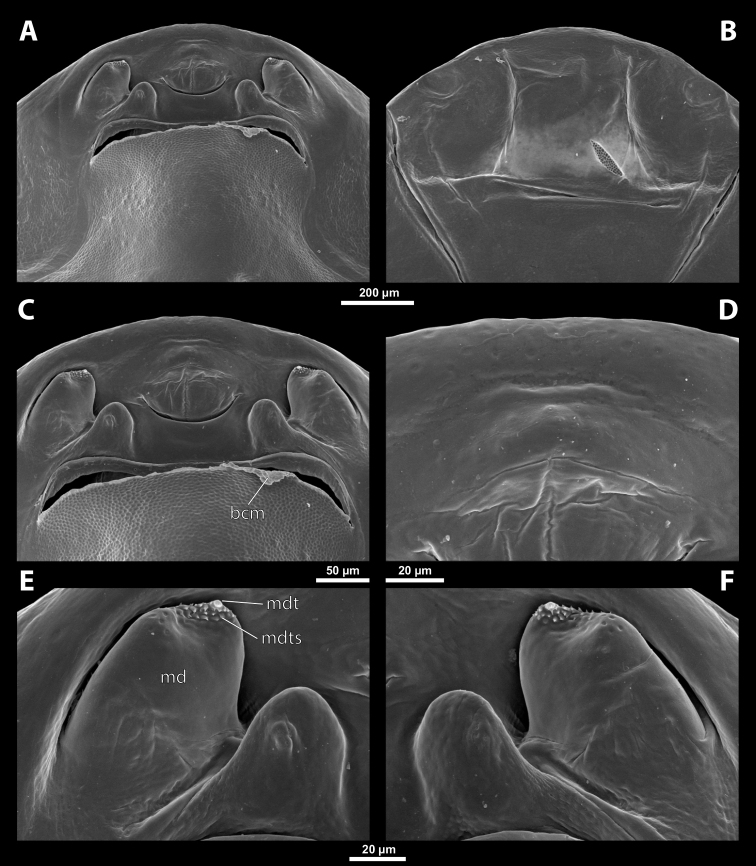
Tachytixenoscf.indicus Pierce, female, cephalothorax, SEM micrographs **A** anterior part of cephalothorax, ventral side **B** anterior part of cephalothorax, dorsal side **C** mouthparts, ventral side **D** detail of anterior border of cephalothorax, ventral side **E** right mandible and maxilla, ventral side **F** left mandible and maxilla, ventral side. Abbreviations: bcm – brood canal membrane, md – mandible, mdt – mandibular tooth, mdts – spine of mandibular tooth.

**Mandible.** Anteromedially directed at angle of 40–65°, enclosed in mandibular capsule. Mandibular bulge not distinctly raised, with several sensilla. Cuticle completely smooth to slightly sculptured. Mandibular tooth very wide on its base, reaching area of mandibular bulge. Tooth ventrally directed and pointed apically. Base with small depressions continuous with several rows of spines (Fig. 14E).

**Maxilla.** Well-developed, prominent, and clearly separated from labial area, strongly sclerotized, directed anteriorly or anteromedially. Not or very slightly overlapping with mandible proximally, not projecting beyond mandibular apex anteriorly. Cuticle usually smooth, rarely wrinkled. Vestige of palp distinct, forming small bulge with more or less distinct plates, situated medially on ventral side of maxilla. Submaxillary groove slightly produced posterolaterally.

**Labium.** Labial area between maxillae distinct, delimited anteriorly by mouth opening and posteriorly by birth opening. Wider than long in midline and flat or convex. Cuticular surface smooth or slightly reticulated.

**Mouth opening.** Mouth opening arcuate, sclerotized along margin.

**Thorax and abdominal segment I.** Pro-mesothoracic and meso-metathoracic borders more or less distinct, usually separated by mesal furrows, often combined with pigmented stripes or spots on dorsal side. Border between metathorax and abdomen usually formed by ridge. Cuticle of thoracic segments on ventral side reticulate, with small scattered pigmented papillae. Dorsal side of thorax smooth or slightly reticulated. Prosternal extension undifferentiated, evenly arched. Shape of meso- and metathorax unmodified, transverse. Setae present on lateral region of abdominal segment I. Cuticular surface distinctly sculptured in cases with sparse setation (Fig. 13E).

**Spiracles.** Located on posterior ~ ⅓ of cephalothorax, slightly elevated, with lateral, anterolateral, or dorsal orientation.

##### Diagnosis of male cephalotheca.

**Figure 15.**
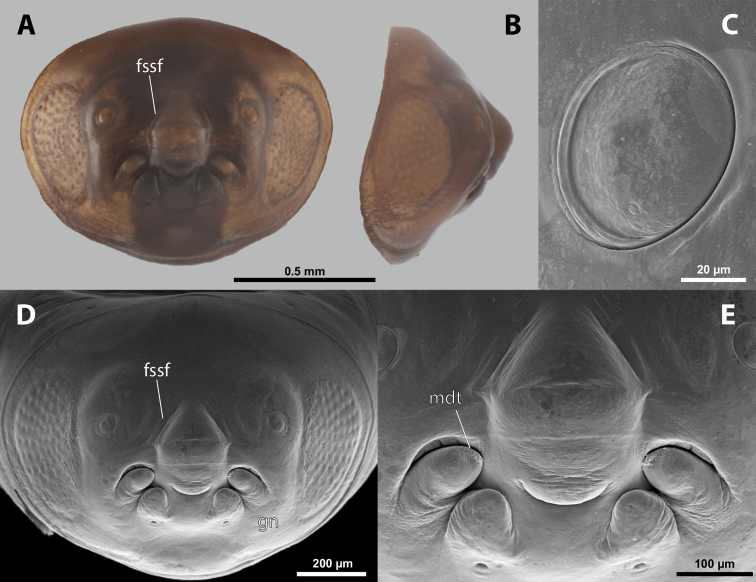
Tachytixenoscf.indicus Pierce, male, cephalotheca, photomicrographs, SEM micrographs **A** frontal view **B** lateral view **C** vestigial antenna **D** frontal view **E** mouthparts. Abbreviations: fssf – furrow of supra-antennal sensillary field, gn – gena, mdt – mandibular tooth.

Genus characterized by combination of distinct paired furrow of supra-antennal sensillary field (Fig. 15A, D) and shape of mandibular tooth. Mandibular tooth very wide on its base and reaching area of mandibular bulge. Tooth base with small depressions continuous with several rows of spines (Fig. 15E, see also 14E). Diameter of genae between maxillary base and compound eye at least 2× as large as diameter of vestigial antenna.

##### Description of male cephalotheca.

**Shape and coloration.** Shape of cephalotheca rounded laterally in frontal view, widely elliptic. Anteriorly pointed in lateral view. Coloration forming pattern of pale and dark shades.

**Cephalothecal capsule.** Compound eyes with darker individual ommatidia well visible on pale background. Clypeal lobe straight in frontal view, distinctly prominent in lateral view. Sensilla mainly concentrated on clypeal lobe. Frontal region with paired furrow of supra-antennal sensillary field, lacking frontal impression. Diameter of genae between maxillary base and compound eye large, ~ 3× as large as diameter of vestigial antenna. Occipital bulge absent.

**Supra-antennal sensillary field.** Kidney-shaped and bulging, delimited medially by distinct furrow. Furrows relatively wide and not interconnected anteriorly (Fig. 15A, D).

**Antenna.** Of standard shape, small, with complete torulus. Periantennal area not distinctly delimited. Sensilla present (Fig. 15C).

**Labrum.** Labral area distinct. Setae on dorsal field present.

**Mandible.** Mandible anteromedially directed. Mandibular tooth very wide on its base and reaches area of mandibular bulge. Tooth base with small depressions continuing in several rows of spines (Fig. 15E). Mandibular bulge bears several sensilla.

**Maxilla.** Maxilla distinct, prominent, completely dark. Vestige of maxillary palp distinct.

**Labium and hypopharynx.** Well-developed between and below maxillae, completely dark. Praementum and postmentum slightly separated by furrow. Hypopharyngeal protuberance absent.

**Mouth opening.** Mouth opening well visible, not covered by ventral labral field, slightly arcuate.

##### Phylogenetic relationships.

One of the earliest diverging lineages of Xenidae. Forming a clade of Palearctic origin with its sister genus *Paraxenos* (Benda et al. 2019).

##### Diversity and distribution.

Monotypic, restricted to the Old World.

##### Hosts.

*Tachytes* spp. (Crabronidae: Crabroninae).

##### Comments.

The monotypic genus *Tachytixenos* was described by Pierce (1911) but only superficial descriptions of the female and male without illustrations were provided. Hofeneder (1949) synonymized it with *Pseudoxenos*, but it was later classified as *Paraxenos* by Kinzelbach (1971b). We restored *Tachytixenos* from synonymy and classify it as a valid genus based on monophyly revealed by the molecular phylogeny (Benda et al. 2019, 2021) and based on morphological characters newly reported here

##### Note.

Cook (1919) noted that Bohart synonymized *Tachytixenos* with *Pseudoxenos* but it was done laterally by Hofeneder (1949).

### ﻿List of species

#### ﻿ Tachytixenos indicus

Taxon classificationAnimaliaStrepsipteraXenidae

Pierce, 1911

1F9A9086-FB5A-5D9D-8DF7-8ABBA9921E17

Tachytixenos indicus Pierce, 1911: 502.
Pseudoxenos indicus (Pierce, 1911) (new combination by Hofeneder 1949).
Paraxenos indicus (Pierce, 1911) (new combination by Kinzelbach 1971b).

##### Hosts.

*Tachytesxenoferus* Rohwer, 1911; *T.maculicornis* Saunders, 1910; *T.modestus* Smith, 1856 (Pierce 1911; Kinzelbach 1978; Kifune and Hirashima 1980); *T.vischnu* Cameron (Cook 2019).

##### Distribution.

Algeria; India: Deesa; Thailand: Peninsular Siam; China; Sri Lanka (Pierce 1911; Kinzelbach 1978; Kifune and Hirashima 1980); Denmark? (Cook 2019).

##### Note.

Benda et al. (2021) reported two lineages possibly representing separate species. A more comprehensive sampling and a detailed study are necessary for a taxonomic revision of this genus.

#### ﻿ Paraxenos

Taxon classificationAnimaliaStrepsipteraXenidae

Saunders, 1872

97DA3E0B-CED8-51D7-9550-E71CE1C08F07

Paraxenos Saunders, 1872: 45. Type species: Paraxenoserberi Saunders, 1872, subsequent designation by Pierce (1908).
Paraxenos (Bembicixenos) (Székessy, 1955: 280) (considered as subgenus by Kinzelbach 1971b: 162).
Bembicixenos Székessy, 1955: 280 (synonymized by Kinzelbach 1978: 82). Type species: Pseudoxenos (Bembicixenos) hungaricus Székessy, 1955, by original designation.

##### Diagnosis of female cephalothorax.

Differing from *Tachytixenos* by a narrower mandibular tooth and a differentiated prosternal extension. Prosternum with anterior swelling (Fig. 18A) similar to *Paragioxenos*, or with distinct color pattern. Clypeal sensilla well visible, extending to ventral side of clypeal area. Vestige of antenna preserved as cavity (Fig. 17D), additional rounded plates rarely present. Maxillae of two types, fused with labial area or distinctly separated and prominent as in *Tachytixenos*, *Pseudoxenos*, and *Tuberoxenos*. In contrast to *Paragioxenos*, head and prothorax ventrally delimited by birth opening medially and by suture laterally.

##### Description of female cephalothorax.

**Figure 16.**
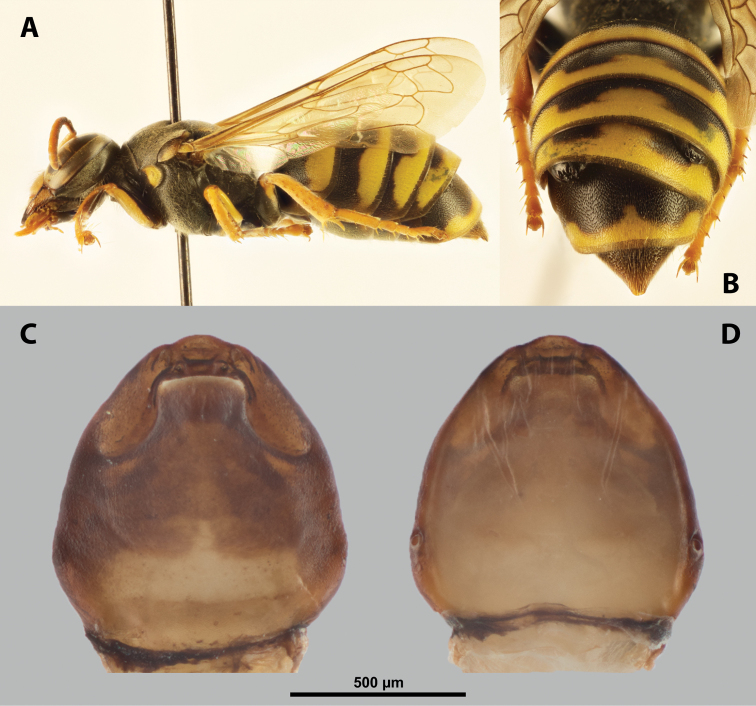
*Paraxenoserberi* Saunders, host, male, female, cephalothorax, photomicrographs **A***Bembecinusperegrinus* (Smith) stylopized by *P.erberi*, lateral view **B** detail of host abdomen with female under third tergite and male puparium under fourth tergite **C** ventral side of female cephalothorax **D** dorsal side of female cephalothorax.

**Shape and coloration.** Compact, very variable in shape, distinctly longer than wide, or wider than long. Size very variable, length 0.94–1.9 mm, maximum width 0.8–2.57 mm. Anterior head margin distinctly protruding. Thorax slightly widening posteriorly, sometimes subparallel. Coloration varying from light to dark brown. Cephalothorax displaying multiple brown shades forming distinct patterns (Fig. 16C, D).

**Head capsule.** Ca. ⅓–½ as long as entire cephalothorax including lateral extensions. Coloration pale to dark, always with species specific patterns. Clypeal area not delimited or well separated from labral area, protruding anteriorly, always forming clypeal lobe. Surface smooth or very slightly wrinkled. Very distinct sensilla mainly concentrated on clypeal lobe and extending to ventral side of clypeal area. Border between clypeal and frontal region usually not clearly recognizable but present, rarely more distinct. Frontal region distinctly wrinkled or covered by papillae. Segmental border between head and prothorax very indistinct on dorsal side, in most specimens virtually unrecognizable.

**Figure 17.**
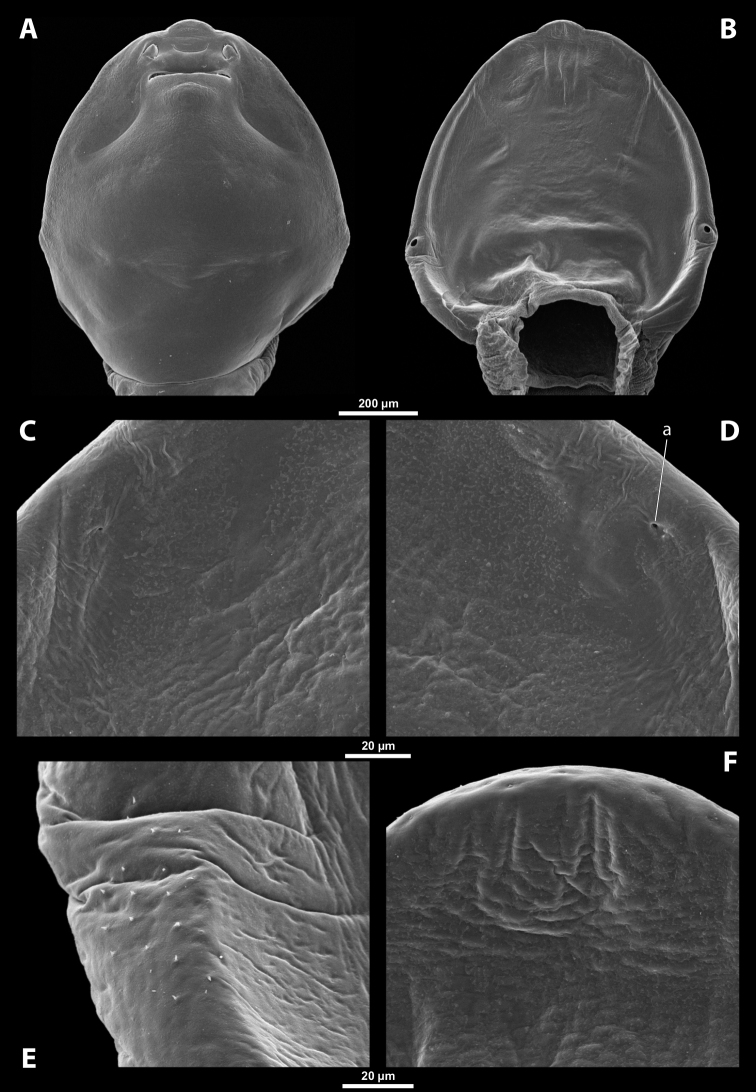
*Paraxenos* sp., female, cephalothorax, SEM micrographs **A** ventral side **B** dorsal side **C** left vestigial antenna, dorsal side **D** right vestigial antenna, dorsal side **E** left lateral border of abdominal segment I below spiracle, dorsal side **F** detail of anterior border of cephalothorax, dorsal side. Abbreviation: a – vestigial antenna.

**Figure 18.**
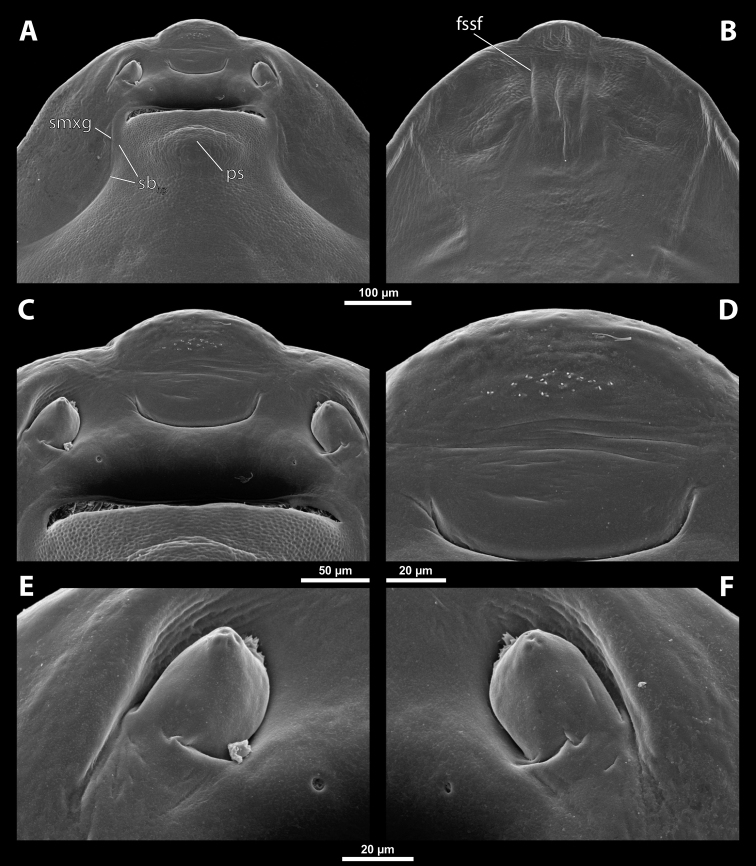
*Paraxenos* sp., female, cephalothorax, SEM micrographs **A** anterior part of cephalothorax, ventral side **B** anterior part of cephalothorax, dorsal side **C** mouthparts, ventral side **D** detail of anterior border of cephalothorax, ventral side **E** right mandible and maxilla, ventral side **F** left mandible and maxilla, ventral side. Abbreviations: fssf – furrow of supra-antennal sensillary field, ps – prosternal swelling, sbhp – segmental border between head and prothorax, smxg – submaxillary groove.

**Supra-antennal sensillary field.** Smooth or slightly wrinkled, with dispersed sensilla, delimited by distinct furrow on medial side (Fig. 18B).

**Antenna.** Preserved as cavity (Fig. 17D), rarely combined with rounded plates. Antennal sensilla or vestigial setae missing. Periantennal area smooth, sometimes reduced when supra-antennal sensillary field almost reaches vestige of antennae.

**Labrum.** Ventral field distinctly wider than long, elliptical or semicircular. Dorsal field arcuate to nearly straight, > 3× wider than long in midline. Dorsal field with ~ 20–25 setae inserted in cavities.

**Mandible.** Anteromedially directed at an angle of 30–65°, enclosed in mandibular capsule or rarely protruding from it. Mandibular bulge not distinctly raised, with ~ 5–18 sensilla. Cuticle completely smooth, or partially sculptured on articulatory area. Mandibular tooth narrow or slightly widened, pointed or blunt, armed with distinct spines.

**Maxilla.** Very variable, well-developed and separated from labial area, or fused with it and strongly reduced. Cuticle always smooth. Prominent, anteriorly or anteromedially directed, in some cases partially overlapping with mandible proximally. Distal maxillary region not projecting beyond mandible anteriorly. Vestige of palp distinct, forming cavity or small bulge with more or less distinct plate. Located anteriorly or medially on ventral side of maxilla. Submaxillary groove distinctly produced posterolaterally (Fig. 18A).

**Labium.** Labial area between maxillae distinct, delimited anteriorly by mouth opening and posteriorly by birth opening. Wider than long in midline and flat. Cuticular surface smooth or slightly reticulated.

**Mouth opening.** Distinctly arcuate to straight, sclerotized around margin.

**Thorax and abdominal segment I.** Pro-mesothoracic and meso-metathoracic borders more or less distinct, usually separated by mesal furrows on ventral side, rarely combined with pigmented stripes or spots on dorsal side, but not recognizable dorsally in most specimens. Border between metathorax and abdomen usually formed by ridge. Cuticle of thoracic segments reticulate on ventral side, often with small, scattered pigmented papillae. Dorsal side of thorax smooth or slightly reticulated. Prosternal extension anteriorly with arcuate to semicircular swelling in most species, or lacking swelling but with distinct color pattern. Meso- and metathorax unmodified in shape, transverse. Setae and cuticular spines present on lateral region of abdominal segment I (Fig. 17E).

**Spiracles.** On posterior third of cephalothorax, slightly elevated, with anterolateral or anterodorsal orientation.

##### Diagnosis of male cephalotheca.

**Figure 19.**
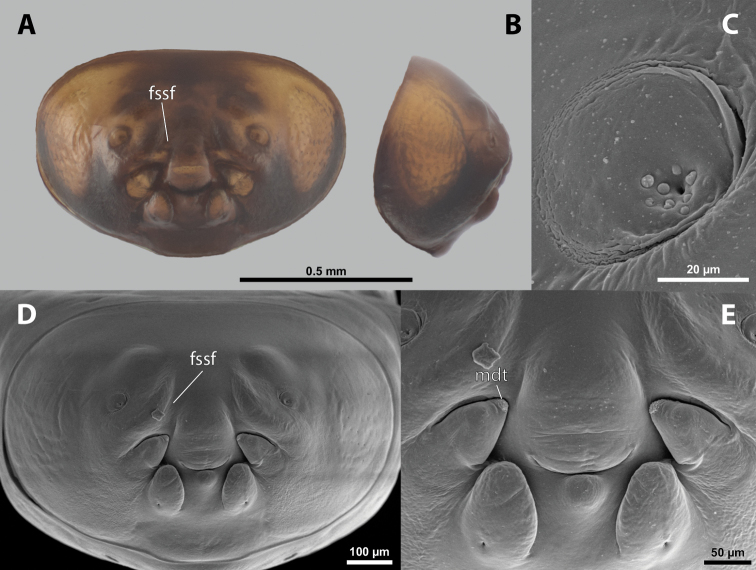
*Paraxenoserberi* Saunders, male, cephalotheca, photomicrographs, SEM micrographs **A** frontal view **B** lateral view **C** vestigial antenna **D** frontal view **E** mouthparts. Abbreviations: fssf – furrow of supra-antennal sensillary field, mdt – mandibular tooth.

Characterized by distinct and relatively wide furrow of supra-antennal sensillary field (Fig. 19A, D). Differing from sister genus *Tachytixenos* in shape of the mandibular tooth, which is conspicuously pointed and not in contact with mandibular bulge. Diameter of genae between maxillary base and compound eye 2× or several times larger than diameter of vestigial antenna. Cephalotheca of elliptic shape in frontal view.

##### Description of male cephalotheca.

**Shape and coloration.** Elliptic and rounded laterally in frontal view, also almost rounded in lateral view. Coloration forming pattern of pale and dark shades.

**Cephalothecal capsule.** Compound eyes with darker individual ommatidia well visible on pale background. Clypeal lobe straight in frontal view, not prominent in lateral view. Sensilla dispersed on clypeal surface. Frontal region with paired furrow of supra-antennal sensillary field, lacking impression or occipital bulge. Diameter of genae between maxillary base and compound eye very large, > 3× as large as diameter of vestigial antenna.

**Supra-antennal sensillary field.** Kidney-shaped and bulging, delimited medially by distinct furrow. Furrows relatively wide, not connected anteriorly (Fig. 19A, D).

**Antenna.** Of standard shape, small, with small plates and cavity (Fig. 19C), torulus interrupted. Periantennal area not clearly delimited from supra-antennal sensillary field.

**Labrum.** Labral area distinct. Setae present on dorsal field.

**Mandible.** Anteromedially directed. Tooth apically pointed, not very wide basally, not reaching area of mandibular bulge (Fig. 19E), which bears sensilla.

**Maxilla.** Distinct, prominent. Coloration pale centrally and dark laterally. Vestige of palp distinct, dark.

**Labium and hypopharynx.** Labium distinct between and below maxillae, dark. Praementum and postmentum indistinctly separated by furrow. Hypopharyngeal protuberance present or not.

**Mouth opening.** Well visible, not covered by ventral labral field, slightly arcuate.

##### Phylogenetic relationships.

Forming a clade of Palearctic origin with *Tachytixenos* (Benda et al. 2019).

##### Diversity and distribution.

Thirteen described species, distributed in the Old World and Australia.

##### Hosts.

*Bembecinus*, *Bembix* and *Stizus* spp. (Bembicidae: Bembicinae).

##### Comments.

*Paraxenos* was described by Saunders (1872) but only a superficial description of the male was provided. Kinzelbach (1971b) synonymized several additional genera with *Paraxenos*, all of them described by Pierce (1908, 1909, 1911, 1919) from the New World (*Eupathocera*, *Opthalmochlus*, *Homilops*, *Sceliphronechthrus*) and Old World (*Tachytixenos*). He also classified *Bembicixenos* described by Székessy (1955) as subgenus of *Paraxenos*, but later considered it a synonym of *Paraxenos* (Kinzelbach 1978). We classify *Paraxenos* as a valid genus based on monophyly revealed by a molecular phylogeny (Benda et al. 2019, 2021) and based on morphological characters newly reported here.

### ﻿List of species

#### ﻿ Paraxenos australiensis

Taxon classificationAnimaliaStrepsipteraXenidae

Kifune & Hirashima, 1987

959F04CA-E5F6-5931-B9D4-A16E75637455

Paraxenos australiensis Kifune & Hirashima, 1987: 157.

##### Host.

*Bembixmusca* (Handlirsch, 1893) (Kifune and Hirashima 1987).

##### Distribution.

Australia: Queensland (Kifune and Hirashima 1987).

#### ﻿ Paraxenos beaumonti

Taxon classificationAnimaliaStrepsipteraXenidae

(Pasteels, 1951)

652465DC-5EC3-5E65-825F-B2537FDB6461

Pseudoxenos beaumonti Pasteels, 1951: 76.
Paraxenos beaumonti (Pasteels, 1951) (new combination by Kinzelbach 1971b).

##### Host.

*Stizusmarthae* Handlirsch, 1892 (Pasteels 1951).

##### Distribution.

Algeria (Pasteels 1951).

#### ﻿ Paraxenos biroi

Taxon classificationAnimaliaStrepsipteraXenidae

(Székessy, 1956)

5F3F70CF-0724-5048-AC92-48B67CACAC47

Pseudoxenos biroi Székessy, 1956: 147.
Paraxenos biroi (Székessy, 1956) (new combination by Kinzelbach 1971b).

##### Host.

*Bembecinusantipodum* (Handlirsch, 1892) (Székessy 1956).

##### Distribution.

New Guinea (Székessy 1956).

#### ﻿ Paraxenos erberi

Taxon classificationAnimaliaStrepsipteraXenidae

Saunders, 1872

35D2DE75-C1C7-528C-B88E-6B915036B9ED

Paraxenos erberi Saunders, 1872: 46.
Pseudoxenos crassidens Pasteels, 1954 (synonymized by Kinzelbach 1978).

##### Hosts.

*Bembecinushungaricus* (Frivaldsky, 1876); *Bembecinusperegrinus* (Smith, 1856); *Bembecinustridens* (Fabricius, 1781) (Saunders 1872; Kinzelbach 1978).

##### Distribution.

Algeria; Europe (Kinzelbach 1978).

#### ﻿ Paraxenos hofenederi

Taxon classificationAnimaliaStrepsipteraXenidae

(Pasteels, 1956)

4B094FDB-95D5-5973-AC16-74A3367CBDD5

Pseudoxenos hofenederi Pasteels, 1956: 111.
Paraxenos hofenederi (Pasteels, 1956) (new combination by Kinzelbach 1971b).

##### Hosts.

*Spheciusnigricornis* (Dufour, 1838), *Stizusbiclypeatus* (Christ, 1791), *Stizusbizonatus* Spinola, 1839, *Stizuspubescens* (Klug, 1835), *Stizusruficornis* (Fabricius, 1787) (Pasteels 1956; Kinzelbach 1978).

##### Distribution.

Algeria; Cyprus; Egypt; Greece; India; Jordan; Tajikistan (Kinzelbach 1978; Batelka and Straka 2005); Senegal? (Kinzelbach 1978).

#### ﻿ Paraxenos hofenederianus

Taxon classificationAnimaliaStrepsipteraXenidae

Luna de Carvalho, 1978

F6F24B6C-2985-5BA6-8A62-339C8590F84C

Paraxenos hofenederianus Luna de Carvalho, 1978: 95.

##### Host.

*Stizusruficornis* (J. Förster, 1771) (as *Stizusdistinguendus* Handlirsch, 1901) (Luna de Carvalho 1978a).

##### Distribution.

Senegal (Luna de Carvalho 1978a).

#### ﻿ Paraxenos hungaricus

Taxon classificationAnimaliaStrepsipteraXenidae

(Székessy, 1955)

FC216284-D4AD-5FA2-81FB-E70BF0BB6C67

Pseudoxenos (Bembicixenos) hungaricus Székessy, 1955: 281.
Paraxenos hungaricus (Székessy, 1955) (new combination by Kinzelbach 1971b).

##### Hosts.

*Bembixoculata* Panzer, 1801, *Bembixrostrata* (Linnaeus, 1758), *Bembix* sp. (Kinzelbach 1978).

##### Distribution.

Czech Republic; Germany; Hungary; Italy; Mongolia; Spain (Székessy 1955; Kinzelbach 1978; Benda et al. 2021); Turkey (this study).

#### ﻿ Paraxenos krombeini

Taxon classificationAnimaliaStrepsipteraXenidae

Kifune & Hirashima, 1987

79240736-6C00-56B1-8EFE-0F84155B848A

Paraxenos krombeini Kifune & Hirashima, 1987: 155.

##### Host.

*Bembixorientalis* (Handlirsch, 1893) (Kifune and Hirashima 1987).

##### Distribution.

Sri Lanka (Kifune and Hirashima 1987).

#### ﻿ Paraxenos nagatomii

Taxon classificationAnimaliaStrepsipteraXenidae

Kifune, 1985

C245F4FE-EFDB-5FEA-ADE9-D6E3F74EDF36

Paraxenos nagatomii Kifune & Yamane, 1985: 49.

##### Host.

*Bembecinusbimaculatus* (Matsumura & Uchida, 1926) (Kifune and Yamane 1985).

##### Distribution.

Japan (Kifune and Yamane 1985).

#### ﻿ Paraxenos novaeguineae

Taxon classificationAnimaliaStrepsipteraXenidae

(Székessy, 1956)

B49DB33A-7C9E-5F6F-9977-F01C60461D79

Pseudoxenos novaeguineae Székessy, 1956: 147.
Paraxenos novaeguineae (Székessy, 1956) (new combination by Kinzelbach 1971b).

##### Host.

*Bembecinusgazagnairei* (Handlirsch, 1892) (Székessy 1956).

##### Distribution.

New Guinea (Székessy 1956).

#### ﻿ Paraxenos occidentalis

Taxon classificationAnimaliaStrepsipteraXenidae

Kifune & Hirashima, 1987

C59C69FE-F865-548C-B100-7DD8186600B3

Paraxenos occidentalis Kifune & Hirashima, 1987: 156.

##### Host.

*Bembixatrifrons* (F. Smith, 1956) (Kifune and Hirashima 1987).

##### Distribution.

Australia: Western Australia (Kifune and Hirashima 1987).

#### ﻿ Paraxenos polli

Taxon classificationAnimaliaStrepsipteraXenidae

(Pasteels, 1956)

B4757A7B-D44B-581E-B5B8-BCA23F20CC07

Pseudoxenos polli Pasteels, 1956: 109.
Paraxenos polli (Pasteels, 1956) (new combination by Kinzelbach 1971b).

##### Host.

*Bembecinusbraunsii* (Handlirsch, 1894) (as *Spheciusfraunsi* Handlirsch, 1894) (Pasteels 1956).

##### Distribution.

Democratic Republic of Congo (Pasteels 1956).

#### ﻿ Paraxenos rieki

Taxon classificationAnimaliaStrepsipteraXenidae

(Pasteels, 1956)

BDA8B557-4C80-53B9-AF83-C6FB13B5596F

Pseudoxenos rieki Pasteels, 1956: 113.
Paraxenos rieki (Pasteels, 1956) (new combination by Kinzelbach 1971b).

##### Host.

*Stizusbasalis* Guérin-Méneville, 1844 (Pasteels 1956).

##### Distribution.

Mali: Djenné (Pasteels 1956).

#### ﻿ Brasixenos

Taxon classificationAnimaliaStrepsipteraXenidae

Kogan & Oliveira, 1966, stat. res.

BA7C77AF-5024-5248-96C3-B7DE6E89776A

Brasixenos Kogan & Oliveira, 1966: 358. Type species: Brasixenosfluminensis Kogan & Oliveria, 1966, by original designation.
Xenos Rossi, 1793 (partim!) (synonymy proposed by Kinzelbach 1971b: 160).
Brasixenos Kogan & Oliveira, 1966 (restored from synonymy by Trois 1988: 268).
Xenos Rossi, 1793 (partim!) (synonymy proposed by Cook 2019: 232).

##### Diagnosis of female cephalothorax.

Maxilla distinctly reduced, flattened, anteriorly rounded, not distinctly prominent; fused to labial area but well defined by its strong sclerotization, conspicuous compared to usually pale cephalothorax as in *Nipponoxenos* and some species of *Xenos*. Maxillary bases appear connected and fused to each other. Vestigial palps differ from those of all other genera, preserved only as inconspicuous concavity on wrinkled maxillary surface, without any vestigial plate. Located anteriorly on ventral side of maxilla, at level of mandibles (Fig. 22E). Clypeal area not delimited from labral area, apparently more or less fused (Fig. 22D). Mandible nested in capsule. In contrast to *Paragioxenos*, head and prothorax ventrally delimited by birth opening medially and by suture laterally.

##### Description of female cephalothorax.

**Shape and coloration.** Compact and usually ovoid, ca. as long as wide, or slightly wider, rarely longer than wide. Abdominal segment I of some species extruded laterally, forming corner below abdominal spiracles. Species relatively variable in size, length 0.76–1.62 mm, maximum width 0.72–1.74 mm. Anterior head margin evenly rounded or protruding. Thorax slightly to strongly widening posteriorly, sometimes subparallel. Coloration mostly pale, with light shadows of brown dominating. Some parts of cephalothorax, especially maxillae, dark and sclerotized.

**Head capsule.** Including lateral extensions ~ ⅓–½ as long as entire cephalothorax. Color pattern formed by shades of pale and dark brown, with maxillae always dark. Clypeal area not delimited from labral area, apparently more or less fused, slightly or distinctly protruding anteriorly, always forming clypeal lobe (Fig. 22D). Surface wrinkled apically on clypeal lobe (sometimes with lamellar structures), smooth ventrolaterally and dorsally. Clypeal surface with ~ 50–70 sensilla or more. Border between clypeal and frontal region indistinguishable. Frontal area smooth. Segmental border between head and prothorax difficult to recognize on dorsal side in some specimens.

**Supra-antennal sensillary field.** Smooth or slightly wrinkled, with dispersed sensilla. Not delimited or indistinctly by furrow on medial side.

**Antenna.** Preserved only as elongated depression or inconspicuous furrow (Fig. 21C). Rounded plate, small cavity or sensilla missing. Periantennal area slightly wrinkled or smooth.

**Labrum.** Ventral field slightly wider than long, nearly circular. Dorsal field anterior to mouth opening slightly arcuate, at least 4× wider than long at midline, with setae inserted in cavities on surface.

**Mandible.** Anteriorly to anteromedially directed at angle of 40–70°, enclosed in capsule. Mandibular bulge sometimes indistinct, with up to ten spine-shaped or blunt sensilla, or lacking these structures. Cuticle completely sculptured or partially smooth. Tooth narrow, armed with several rows of spines.

**Figure 20.**
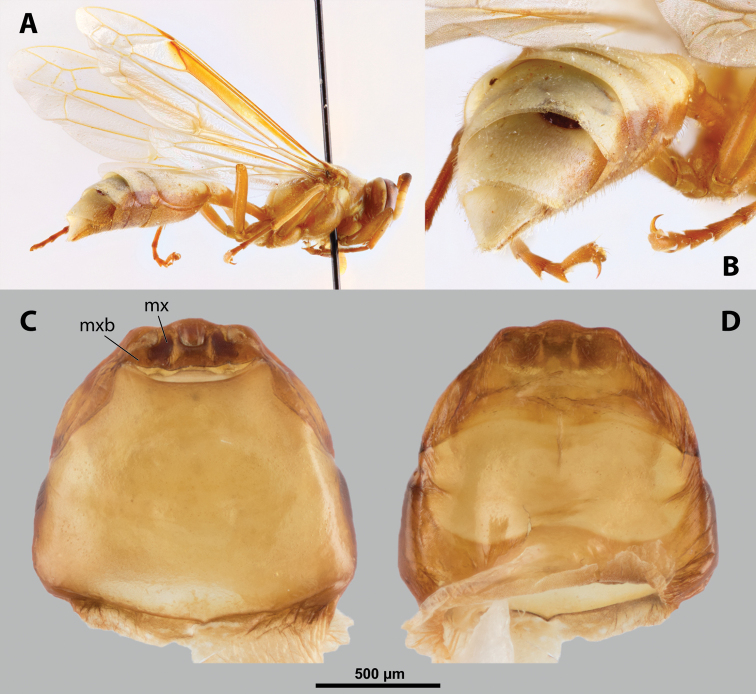
*Brasixenosaraujoi* (Oliveira & Kogan), host, female, cephalothorax, photomicrographs **A***Apoicapallens* (Fabricius) stylopized by female of *B.araujoi*, lateral view **B** detail of host abdomen with adult female inside **C** ventral side of cephalothorax **D** dorsal side of cephalothorax. Abbreviations: mx – vestige of maxilla, mxb – maxillary base.

**Maxilla.** Reduced and not protruding, fused to labium but clearly indentifiable by distinct sclerotization; appearing connected and fused medially, with sclerotization continuous along birth opening. Cuticle distinctly wrinkled. Apical maxillary region almost reaching upper edge of mandible in some species. Vestige of palp present as inconspicuous cavity on wrinkled maxillary surface, lacking vestigial plate. Located anteriorly on ventral side, at level of mandibles. Maxillary base slightly raised and less sclerotized than anterior region (Fig. 20C). Submaxillary groove slightly produced posterolaterally.

**Labium.** Labial area recognizable between maxillae but fused with them, anteriorly delimited by mouth opening; convex, wider than long in midline, pale laterally, strongly sclerotized medially and around mouth opening. Cuticular surface smooth or wrinkled, with wrinkles indistinct on well sclerotized areas.

**Mouth opening.** Arcuate to distinctly U-shaped, sclerotized around margin.

**Figure 21.**
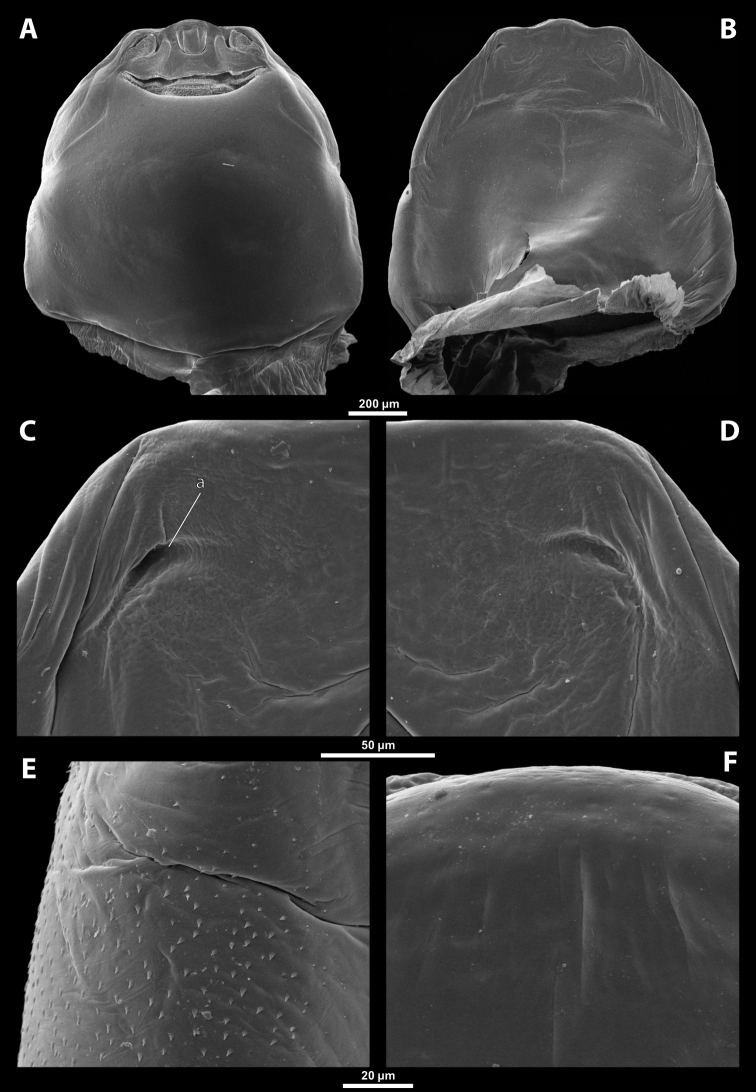
*Brasixenosaraujoi* (Oliveira & Kogan), female, cephalothorax, SEM micrographs **A** ventral side **B** dorsal side **C** left vestigial antenna, dorsal side **D** right vestigial antenna, dorsal side **E** left lateral border of abdominal segment I below spiracle, dorsal side **F** detail of anterior border of cephalothorax, dorsal side. Abbreviation: a – vestigial antenna.

**Thorax and abdominal segment I.** Pro-mesothoracic and meso-metathoracic borders more or less distinct, usually indicated by pigmented stripes or changed coloration on dorsal side. Mesal furrows absent. Border between metathorax and abdomen usually indicated by change in coloration or cuticular sculpture, separating ridge indistinct. Cuticle of thoracic segments with smooth surface on the ventral side, in some cases with small scattered pigmented papillae. Dorsal side of thorax usually completely smooth. Prosternal extension not very distinctly prolonged, usually evenly arched. Thoracic segments constricted laterally, distance between lateral extensions of head and spiracles thus reduced (Fig. 20D). Setae and cuticular spines present on lateral region of abdominal segment I (Fig. 21E).

**Figure 22.**
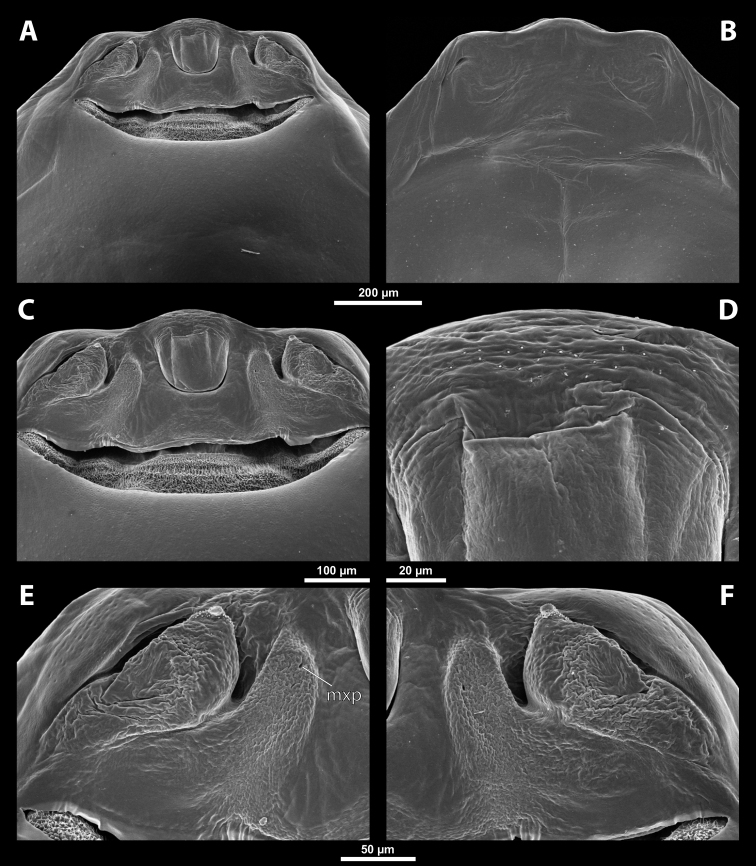
*Brasixenosaraujoi* (Oliveira & Kogan), female, cephalothorax, SEM micrographs **A** anterior part of cephalothorax, ventral side **B** anterior part of cephalothorax, dorsal side **C** mouthparts, ventral side **D** detail of anterior border of cephalothorax, ventral side **E** right mandible and maxilla, ventral side **F** left mandible and maxilla, ventral side. Abbreviation: mxp – vestige of maxillary palp.

**Spiracles.** Spiracles situated on posterior half or posterior third of cephalothorax, slightly elevated, with anterolateral orientation.

##### Diagnosis of male cephalotheca.

**Figure 23.**
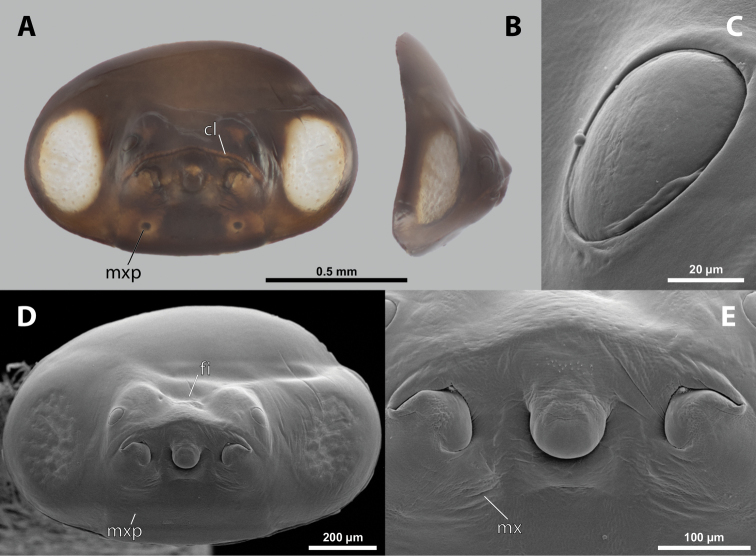
*Brasixenos* sp., male, cephalotheca, photomicrographs, SEM micrographs **A** frontal view **B** lateral view **C** vestigial antenna **D** frontal view **E** mouthparts. Abbreviations: cl – clypeus, fi – frontal impression, mx – vestige of maxilla, mxp – vestige of maxillary palp.

Differing from other genera by fusion of maxilla with cephalotheca. Maxillary cuticular surface with longitudinal grooves (Fig. 23E). Vestige of maxillary palp visible (distinct in optical microscope, very inconspicuous on SEM micrographs) (Fig. 23A, D).

##### Description of male cephalotheca.

**Shape and coloration.** Laterally rounded in frontal view, elliptic, in lateral view pointed anteriorly. Coloration mostly dark, but with some lighter areas such as ocular region or surroundings of maxillary palps (Fig. 23A).

**Cephalothecal capsule.** Compound eyes with darker individual ommatidia well visible on pale ocular background. Clypeus with longitudinal pale line (Fig. 23A). Clypeal lobe arcuate or straight in frontal view, prominent in lateral view; with sensilla evenly dispersed. Frontal region with conspicuous impression (Fig. 23D). Diameter of genae between maxillary base and compound eye large, > 2× as large as diameter of vestigial antenna. Occipital bulge absent.

**Supra-antennal sensillary field.** Kidney-shaped and bulging, medially delimited by frontal impression, with visible but indistinct furrows.

**Antenna.** Of standard shape, small, with complete torulus. Periantennal area indistinct but present. Sensilla usually absent.

**Labrum.** Labral area well visible but dorsal field not clearly separated from clypeus. Setae on dorsal field present.

**Mandible.** Anteromedially directed, pale centrally and dark laterally. Mandibular bulge not conspicuous, with several sensilla.

**Maxilla.** Not recognizable as separate structure, fused with cephalotheca. Cuticular surface of maxillary area sculptured, with longitudinal grooves (Fig. 23E). Vestige of palp well visible (with light microscope, very indistinct on SEM micrographs) (Fig. 23A, D).

**Labium and hypopharynx.** Distinct, inserted between and below maxillae, completely dark. Praementum and postmentum very indistinctly separated. Hypopharyngeal protuberance recognizable, not well delimited.

**Mouth opening.** Well visible, U-shaped, partially covered by ventral labral field.

##### Phylogenetic relationships.

Sister to a large clade containing representatives of genera previously known as *Pseudoxenos*, *Paraxenos*, and *Xenos* (Benda et al. 2019).

##### Diversity and distribution.

Group of Xenidae with origin in the New World and restricted to this region. Comprising seven species, all of which are known from Brazil.

##### Hosts.

Various genera of Epiponini (Vespidae: Polistinae).

##### Comments.

The genus *Brasixenos* was described and differentiated from *Xenos* by Kogan and Oliveira (1966), but the description of the female cephalothorax was superficial. Although Kinzelbach (1971b) treated *Brasixenos* as a junior synonym of *Xenos*, Trois (1988) attempted to reinstate *Brasixenos* as a valid genus. Nevertheless, no author has followed this opinion (Cook 2019). Although Kogan and Oliveira (1966) expected a close relationship of *Xenos* with *Brasixenos* in their description, Benda et al. (2019) revealed the group as a separate lineage unrelated to *Xenos*. We classify *Brasixenos* as a valid genus, based on a molecular phylogeny (Benda et al. 2019, 2021) and morphological characters newly reported here.

### ﻿List of species

#### ﻿ Brasixenos acinctus

Taxon classificationAnimaliaStrepsipteraXenidae

Kogan & Oliveira, 1966, stat. res.

66E14E3F-88B8-5810-8C84-3AA332F9C28F

Brasixenos acinctus Kogan & Oliveira, 1966: 356.
Xenos acinctus (Kogan & Oliveira, 1966) (synonymy proposed by Kinzelbach 1971b).

##### Host.

*Polybia* sp., close to *Polybiasericea* (Olivier, 1792).

##### Distribution.

Brazil: Rio de Janeiro (Kogan and Oliveira 1966).

#### ﻿ Brasixenos araujoi

Taxon classificationAnimaliaStrepsipteraXenidae

(Oliveira & Kogan, 1962), stat. res.

CD243C17-51A9-53DE-84C0-DD4FEBE5B372

Xenos araujoi Oliveira & Kogan, 1962: 6 (combination restored by Kinzelbach 1971b and Cook 2019).
Brasixenos araujoi (Oliveira & Kogan, 1962) (new combination by Kogan and Oliveira 1966 and Trois 1988).

##### Hosts.

*Apoicapallens* (Fabricius, 1804) (Oliveira and Kogan 1962); *Apoicaflavissima* Vecht, 1973; *Apoicathoracica* Buysson, 1906 (this study).

##### Distribution.

Brazil: Amazonas (Oliveira and Kogan 1962).

#### ﻿ Brasixenos bahiensis

Taxon classificationAnimaliaStrepsipteraXenidae

Kogan & Oliveira, 1966, stat. res.

9F10C8C5-9F5C-5020-848A-3A264DEBA060

Brasixenos bahiensis Kogan & Oliveira, 1966: 353.
Xenos bahiensis (Kogan & Oliveira, 1966) (new combination by Kinzelbach 1971b).

##### Host.

*Polybiaignobilis* (Haliday, 1836).

##### Distribution.

Brazil: Bahia (Kogan and Oliveira 1966).

#### ﻿ Brasixenos brasiliensis

Taxon classificationAnimaliaStrepsipteraXenidae

Kogan & Oliveira, 1966, stat. res.

219B0CA0-8838-5F67-9753-A3D93CE9AF06

Brasixenos brasiliensis Kogan & Oliveira, 1966: 355.
Xenos brasiliensis (Kogan & Oliveira, 1966) (new combination by Kinzelbach 1971b).

##### Host.

*Polybiasericea* (Olivier, 1792).

##### Distribution.

Brazil: Rio de Janeiro, Pará (Kogan and Oliveira 1966).

#### ﻿ Brasixenos fluminensis

Taxon classificationAnimaliaStrepsipteraXenidae

Kogan & Oliveira, 1966, stat. res.

07EBC37A-6A01-5E86-A4F7-30F34EE2F73F

Brasixenos fluminensis Kogan & Oliveira, 1966: 347.
Xenos fluminensis (Kogan & Oliveira, 1966) (new combination by Kinzelbach 1971b).

##### Host.

*Polybiaignobilis* (Haliday, 1836) (as *Polybiaatra* Saussure, 1854).

##### Distribution.

Brazil: Rio de Janeiro (Kogan and Oliveira 1966).

#### ﻿ Brasixenos myrapetrus

Taxon classificationAnimaliaStrepsipteraXenidae

Trois, 1988, stat. res.

60ED71AE-6A5E-500A-AE73-72E5492523BB

Brasixenos myrapetrus Trois, 1988: 277.
Xenos myrapetrus (Trois, 1988) (new combination by Cook, 2019).

##### Host.

Polybia (Myrapetra) paulista Ihering, 1896 (Trois 1988).

##### Distribution.

Brazil (Trois 1988).

#### ﻿ Brasixenos zikani

Taxon classificationAnimaliaStrepsipteraXenidae

Kogan & Oliveira, 1966, stat. res.

116C0BA9-B55E-5585-9AB4-2830BD3F1833

Brasixenos zikani Kogan & Oliveira, 1966: 350.
Xenos zikani (Kogan & Oliveira, 1966) (new combination by Kinzelbach 1971b).

##### Host.

*Polybiatinctipennis* Fox, 1898 (as *Polybiaypiranguensis* Ihering, 1904) (Kogan and Oliveira 1966).

##### Distribution.

Brazil: Rio de Janeiro (Kogan and Oliveira 1966).

#### ﻿ Leionotoxenos

Taxon classificationAnimaliaStrepsipteraXenidae

Pierce, 1909, stat. res.

042D78B1-CBD7-50FF-A3A0-6EC1C3BA4530

Leionotoxenos Pierce, 1909: 137. Type species: Leionotoxenosjonesi Pierce, 1909, by original designation.
Pseudoxenos Saunders, 1872 (partim!) (synonymy proposed by Bohart, 1937: 133). Paraxenos Saunders, 1872 (partim!) (synonymy proposed by Kinzelbach 1971b: 162).
Monobiaphila Pierce, 1909: 139 (syn. nov.). Type species: Monobiaphilabishoppi Pierce, 1909, by original designation.
Montezumiaphila Brèthes, 1923: 45 (syn. nov.). Type species: MontezumiaphilavigiliBrèthes 1923, by monotypy.

##### Diagnosis of female cephalothorax.

Differing from its sister genus *Eupathocera* in the following characters. Frontal region with conspicuous coverage of papillae (Fig. 25F). Supra-antennal sensillary field with wrinkled surface, which almost reaches vestigial antenna. Periantennal area small and indistinct (Fig. 25C). Prothorax ventrally connected to head on same plane, versus usually elevated in *Eupathocera* (Fig. 25A). Position of sensilla on clypeal lobe not extended onto ventral side of clypeal area as in *Xenos* or *Paraxenos*. Rudiments of torulus usually preserved (Figs 25C, 29D). Mandible not protruding from mandibular capsule. In contrast to *Paragioxenos*, head and prothorax ventrally delimited by birth opening medially and by suture laterally.

##### Description of female cephalothorax.

**Figure 24.**
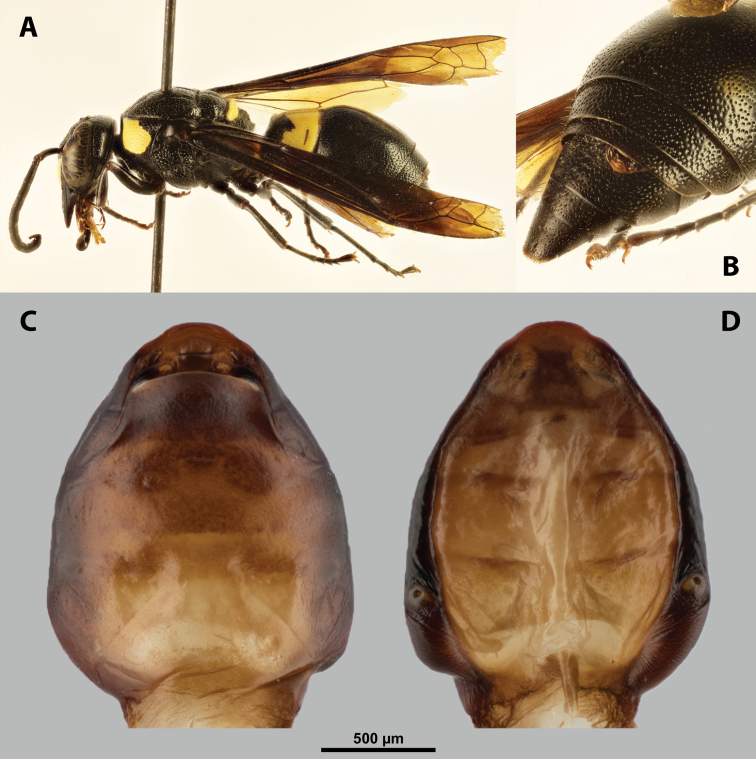
*Leionotoxenosbishoppi* (Pierce), host, female, cephalothorax, photomicrographs **A***Monobiaquadridens* (Linnaeus) stylopized by female of *L.bishoppi*, lateral view **B** detail of host abdomen with adult female inside **C** ventral side of cephalothorax **D** dorsal side of cephalothorax.

**Shape and coloration.** Cephalothorax compact and usually ovoid, varying distinctly in shape, longer than wide to distinctly wider than long. Species relatively variable in size, length 0.88–1.7 mm, maximum width 0.72–1.68. Anterior head margin evenly rounded or slightly protruding anteriorly. Thorax slightly to strongly widening posteriorly, sometimes subparallel. Coloration of cephalothorax with multiple dark and light brown shades forming distinct pattern (Fig. 24C, D).

**Figure 25.**
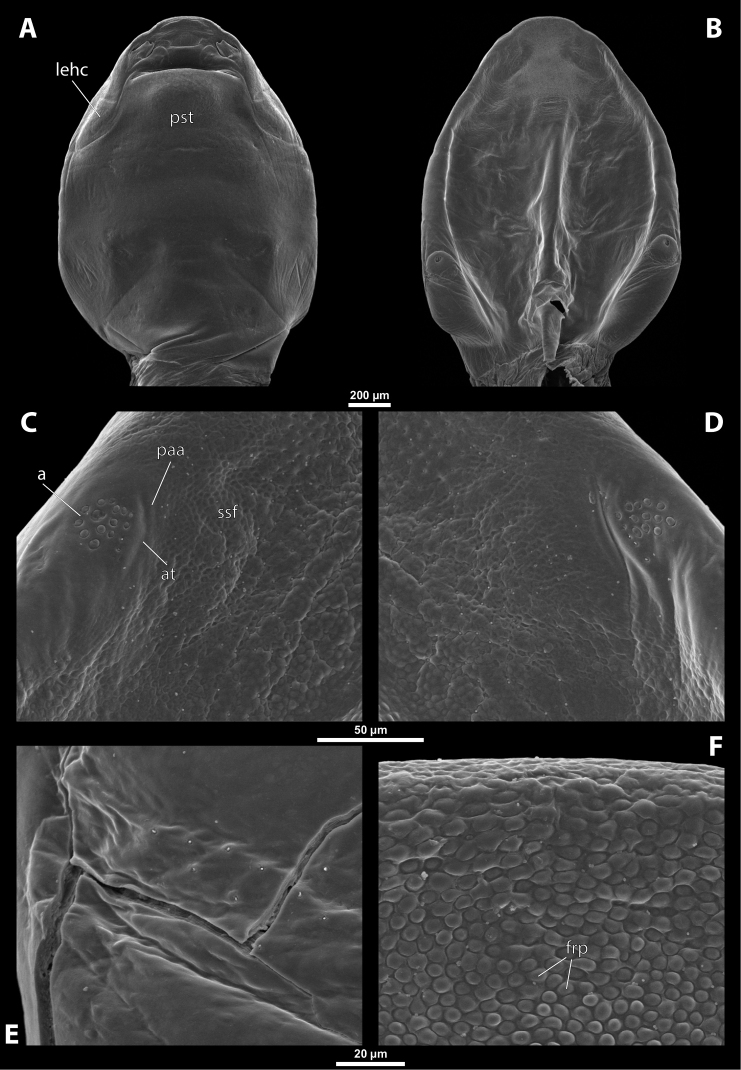
*Leionotoxenosbishoppi* (Pierce), female, cephalothorax, SEM micrographs **A** ventral side **B** dorsal side **C** left vestigial antenna, dorsal side **D** right vestigial antenna, dorsal side **E** left lateral border of abdominal segment I below spiracle, dorsal side **F** detail of anterior border of cephalothorax, dorsal side. Abbreviations: a – vestigial antenna, at – antennal torulus, frp – frontal papillae, lehc – lateral extension of head capsule, paa – periantennal area, pst – prosternum (prosternal extension), ssf – supra-antennal sensillary field.

**Figure 26.**
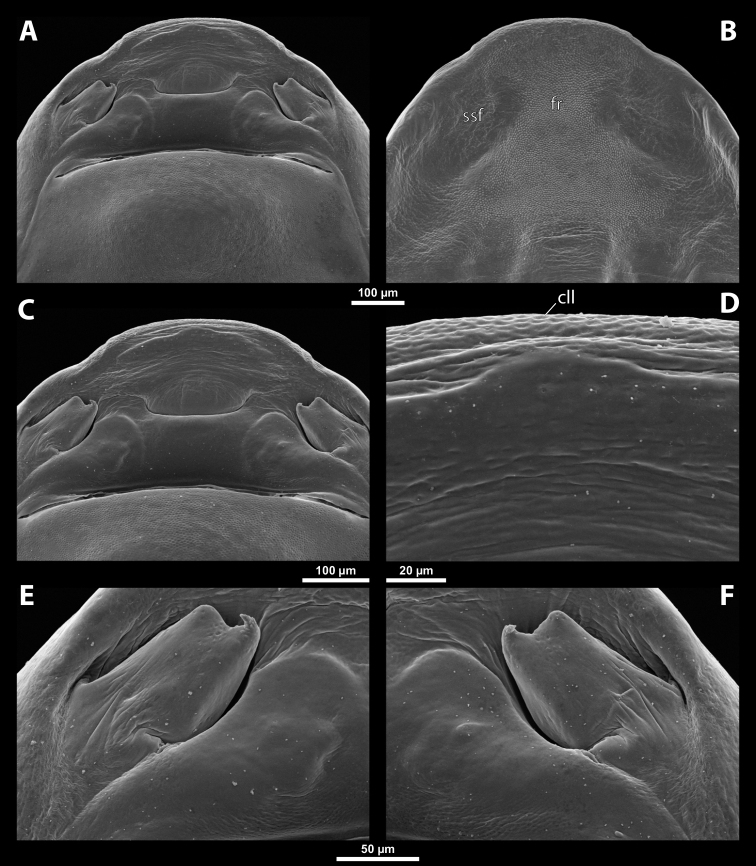
*Leionotoxenosbishoppi* (Pierce), female, cephalothorax, SEM micrographs **A** anterior part of cephalothorax, ventral side **B** anterior part of cephalothorax, dorsal side **C** mouthparts, ventral side **D** detail of anterior border of cephalothorax, ventral side **E** right mandible and maxilla, ventral side **F** left mandible and maxilla, ventral side. Abbreviations: cll – clypeal lobe, fr – frontal region, ssf – supra-antennal sensillary field.

**Head capsule.** Ca. ⅓ to nearly ½ as long as entire cephalothorax including lateral cephalic extensions. Coloration variable, pale to dark brown or forming specific patterns. Clypeal area well delimited from labral region, clypeal lobe indistinct or slightly protruding anteriorly. Surface more or less wrinkled, in some cases with reticulated pattern (Fig. 26D), with 12–26 (or more) sensilla distributed anteriorly. Border between clypeal and frontal region indistinct but still recognizable. Frontal region with conspicuous coverage of papillae (Fig. 25F). Dorsal border between head and prothorax indicated by interrupted suture, distinct coloration, or largely obliterated.

**Supra-antennal sensillary field.** Conspicuously wrinkled or reticulated. Usually delimited by indistinct furrow on medial side, but otherwise by change in cuticular sculpture, with wrinkled surface of supra-antennal sensillary field versus papillae on frontal region (Fig. 26B).

**Antenna.** Preserved as more or less defined area, with several rounded plates and setae (Fig. 25C). Torulus largely reduced or absent, rudiment usually recognizable as interrupted furrow (Fig. 25C). Periantennal area small and indistinct, supra-antennal sensillary field with wrinkled surface almost reaching antennal vestige (Fig. 25C).

**Labrum.** Ventral field wider than long, elliptic. Dorsal field slightly arcuate, at least 3× to 4× wider than medially along midline. Dorsal field with several inconspicuous setae (10 to 20) inserted in cavities.

**Mandible.** Anteromedially directed at an angle of 40–55° and enclosed in capsule. Mandibular bulge more or less distinctly raised, with 5–7 sensilla. Cuticle smooth to slightly sculptured or with longitudinal grooves. Mandibular tooth narrow or slightly widened, with or without spines.

**Maxilla.** Reduced and not distinctly protruding, fused to labium, often not clearly separated from labial area. Cuticle smooth or slightly wrinkled. Maxillary apex not projecting beyond mandible anteriorly. Vestige of palp inconspicuous, forming small bulge, sometimes very indistinct, located medially on ventral side of maxilla. Submaxillary groove more or less distinctly produced posteriorly to maxillary base.

**Labium.** Labial area flat, wider than long in midline or as wide as long, usually recognizable between maxillae but sometimes fused with them. Anteriorly delimited by mouth opening and posteriorly by birth opening. Cuticular surface smooth or slightly reticulated.

**Mouth opening.** Distinctly arcuate to nearly straight, sclerotized marginally.

**Thorax and abdominal segment I.** Pro-mesothoracic and meso-metathoracic borders distinct or indistinct, usually indicated by mesal furrows, often combined with pigmented stripes. Border between metathorax and abdomen usually formed by indistinct ridge or change in cuticular surface. Cuticle of thoracic segments on ventral side reticulate, often with scattered small and pigmented papillae. Dorsal side smooth or slightly wrinkled or reticulated. Prosternal extension either undifferentiated or indicated anteriorly by color pattern, in which case a swelling can be present or absent. Region of prosternal extension evenly connected to head on same plane (Fig. 25A). Meso- and metathorax unmodified in shape, transverse. Setae or cuticular spines present on lateral region of abdominal segment I (Fig. 25E).

**Spiracles.** Located on posterior ~ ⅓ of cephalothorax, slightly elevated, with anterolateral or anterodorsal orientation.

##### Diagnosis of male cephalotheca.

**Figure 27.**
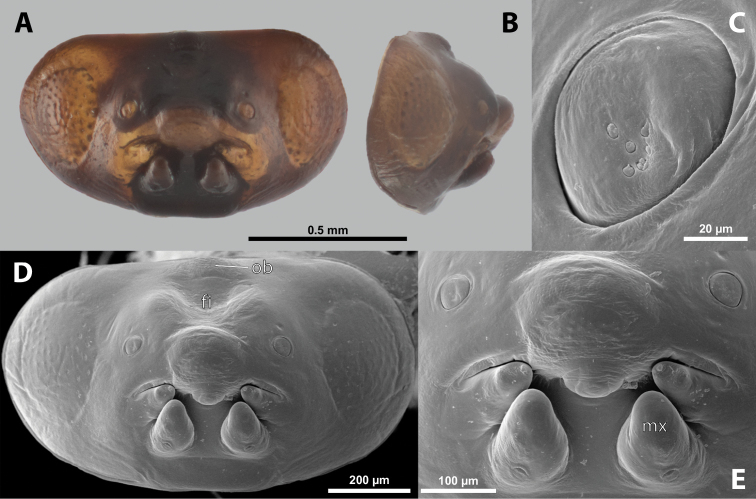
*Leionotoxenos* sp., male, cephalotheca, photomicrographs, SEM micrographs **A** frontal view **B** lateral view **C** vestigial antenna **D** frontal view **E** mouthparts. Abbreviations: fi – frontal impression, mx – vestige of maxilla, ob – occipital bulge.

Differing from other genera in the following characters. Diameter of genae between maxillary base and compound eye at least 2× as large as diameter of vestigial antenna. Distinct paired furrow of supra-antennal sensillary field absent. Cephalotheca always of elliptic shape (Fig. 27A). Frontal fissure very distinct (Fig. 27D). Maxilla prominent, at least 1.5× longer than basally wide (Fig. 27E).

##### Description of male cephalotheca.

**Shape and coloration.** In frontal view rounded laterally, elliptic, in lateral view pointed anteriorly. Coloration with pattern of pale and dark shades.

**Cephalothecal capsule.** Compound eyes with darker individual ommatidia well visible on pale ocular background. Clypeal lobe arcuate in frontal view, prominent in lateral view. Clypeal sensilla mainly concentrated medially on clypeus. Frontal region slightly deformed by frontal impression (Fig. 27D). Occipital bulge present (Fig. 27D). Diameter of genae between maxillary base and compound eye very large, ~ > 3× as large as diameter of vestigial antenna.

**Supra-antennal sensillary field.** Kidney-shaped and bulging, medially delimited by frontal impression, lacking distinctly visible furrows.

**Antenna.** Of standard shape, small, with complete torulus and small plates (Fig. 27C). Periantennal area not clearly delimited from supra-antennal sensillary field.

**Labrum.** Labral area distinct. Setae on dorsal field present.

**Mandible.** Anteromedially directed. Tooth pointed apically, not reaching area of mandibular bulge basally. Bulge set with sensilla.

**Maxilla.** Distinct, prominent, entirely dark. Vestige of palp distinct.

**Labium and hypopharynx.** Distinct, dark, inserted between and below maxillae. Praementum and postmentum clearly separated by furrow. Hypopharyngeal protuberance not present.

**Mouth opening.** Distinctly arcuate but not well visible, covered by ventral labral field.

##### Phylogenetic relationships.

According to Benda et al. (2019) part of a clade of a New World origin, with *Eupathocera* Pierce as sister group.

##### Diversity and distribution.

Fourteen described species, restricted to the New World.

##### Hosts.

Various genera of Odynerini (Vespidae: Eumeninae).

##### Comments.

The genus *Leionotoxenos* was described by Pierce (1909) based on his suggestion that a new genus of Strepsiptera should be established if it utilizes a different host genus. No diagnosis or description was presented. It was later synonymized with *Pseudoxenos* (Bohart 1937) and then with *Paraxenos* (Kinzelbach 1971b). We restore *Leionotoxenos* from synonymy and classify it as a valid genus, based on the molecular phylogeny (Benda et al. 2019, 2021) and morphological characters newly reported here. We classify the names *Monobiaphila* and *Montezumiaphila* as synonyms of *Leionotoxenos*.

### ﻿List of species

#### ﻿ Leionotoxenos arvensidis

Taxon classificationAnimaliaStrepsipteraXenidae

(Pierce, 1911) comb. nov.

7189FAEA-CDFA-5EBE-A098-6BE40E13ECFE

Pseudoxenos arvensidis Pierce, 1911: 499.

##### Hosts.

*Euodynerusannulatusarvensis* (Saussure, 1869) (as Odynerus (Leionotus) arvensis Saussure, 1869) (Pierce 1911), *Euodynerusannulatussulphureus* (Saussure, 1858) (Kinzelbach 1971b).

##### Distribution.

USA: Illinois (Pierce 1911).

#### ﻿ Leionotoxenos bishoppi

Taxon classificationAnimaliaStrepsipteraXenidae

(Pierce, 1909) comb. nov.

088CD561-8F8F-5E5F-9D8D-D0FF2C133017

Monobiaphila bishoppi Pierce, 1909: 139.
Pseudoxenos bishoppi (Pierce, 1909) (new combination by Bohart 1941).

##### Host.

*Monobiaquadridens* (Linnaeus, 1763) (Pierce 1909).

##### Distribution.

USA: Texas (Pierce 1909), Kansas, Pennsylvania (this study).

#### ﻿ Leionotoxenos foraminati

Taxon classificationAnimaliaStrepsipteraXenidae

(Pierce, 1911) comb. nov.

3902FB07-46E5-5C94-B59F-FF46CEBC7304

Pseudoxenos foraminati Pierce, 1911: 499.

##### Host.

*Euodynerusforaminatus* (Saussure, 1853) (as *Odynerusforaminatus* Saussure, 1853) (Pierce 1911).

##### Distribution.

USA: New Jersey (Pierce 1911).

#### ﻿ Leionotoxenos fundati

Taxon classificationAnimaliaStrepsipteraXenidae

(Pierce, 1911) comb. nov.

C8F8900F-622A-5A64-ADD9-D988686D29B9

Pseudoxenos fundati Pierce, 1911: 500.

##### Host.

*Stenodynerusproquinquus* (Saussure, 1870) (as Odynerus (Leionotus) fundatus Cresson, 1872) (Pierce 1911).

##### Distribution.

USA: Ilinois (Pierce 1911).

#### ﻿ Leionotoxenos hookeri

Taxon classificationAnimaliaStrepsipteraXenidae

Pierce, 1909, stat. res.

0C61AAA0-1CC9-59D6-BE95-B8BDF4AD52C0

Leionotoxenos hookeri Pierce, 1909: 139.
Pseudoxenos hookeri (Pierce, 1909) (new combination by Bohart 1937).

##### Hosts.

*Euodynerusannulatus* (Say, 1824) (as *Leionotusverus* (Cresson, 1872)) (Pierce 1909), *Euodynerusforaminatus* (Saussure, 1853) (Krombein 1967).

##### Distribution.

USA: Texas (Pierce 1909).

#### ﻿ Leionotoxenos huastecae

Taxon classificationAnimaliaStrepsipteraXenidae

(Székessy, 1965) comb. nov.

F7CAA2CC-D5A7-5772-8299-F4837CCAE56B

Pseudoxenos huastecae Székessy, 1965: 477.

##### Host.

*Montezumiacentralis* Zavattari, 1912 (as Montezumiahuastecavar.centralis Zavattari, 1912) (Székessy 1965).

##### Distribution.

Honduras (Székessy 1965).

#### ﻿ Leionotoxenos itatiaiae

Taxon classificationAnimaliaStrepsipteraXenidae

(Trois, 1984b) comb. nov.

31031F67-7B8F-5447-9EA4-CEE7A1D19B79

Pseudoxenos itatiaiae Trois, 1984b: 25.

##### Host.

*Eumenes* sp. (Trois 1984b).

##### Distribution.

Brazil, Rio de Janeiro (Trois 1984b).

##### Note.

Probably misidentification of host. *Eumenes* does not occur in South America.

#### ﻿ Leionotoxenos jonesi

Taxon classificationAnimaliaStrepsipteraXenidae

Pierce, 1909, stat. res.

684118F4-E443-51C1-BDEB-60BEDBAC0F07

Leionotoxenos jonesi Pierce, 1909: 138.
Pseudoxenos jonesi (Pierce, 1909) (new combination by Bohart 1937).

##### Host.

*Parancistrocerusvagus* (Saussure, 1857) (as *Leionotuscolon* (Cresson, 1872)) (Pierce 1909).

##### Distribution.

USA: Louisiana, Texas (Pierce 1909).

#### ﻿ Leionotoxenos louisianae

Taxon classificationAnimaliaStrepsipteraXenidae

Pierce, 1909, stat. res.

D00920F6-4B06-5555-9E94-92BDA06C489E

Leionotoxenos louisianae Pierce, 1909: 138.
Pseudoxenos louisianae (Pierce, 1909) (new combination by Bohart 1937).
Pseudoxenos histrionis Pierce, 1911: 500 (synonymized by Bohart 1941).
Pseudoxenos pedestridis Pierce, 1911: 500 (synonymized by Bohart 1941).

##### Hosts.

*Parancistrocerusvagus* (Saussure, 1857) (as *Leionotusvagans* Saussure, 1857); *Parancistrocerushistrio* (Lepeletier, 1841) (as Odynerus (Ancistrocerus) histrio Lepeletier, 1841); *Parancistroceruspedestris* (Saussure, 1855) (as Odynerus (Leionotus) pedestris Saussure, 1855) (Pierce 1909, 1911).

##### Distribution.

USA: Florida, Illinois, Louisiana, Nebraska (Pierce 1909, 1911).

#### ﻿ Leionotoxenos neomexicanus

Taxon classificationAnimaliaStrepsipteraXenidae

(Pierce, 1919) comb. nov.

8AB76D36-386C-56D4-92EF-9AF8756CA5DB

Pseudoxenos neomexicanus Pierce, 1919: 463.

##### Host.

*Stenodynerustoas* (Cresson, 1867) (as *Odynerustaos* Cresson, 1867) (Pierce 1919).

##### Distribution.

USA: New Mexico (Pierce 1919).

#### ﻿ Leionotoxenos prolificum

Taxon classificationAnimaliaStrepsipteraXenidae

(Teson & Remes Lenicov, 1979) comb. nov.

E345B410-159E-5E34-BE11-A94E00E8BFA6

Pseudoxenos prolificum Teson & Remes Lenicov, 1979: 115.

##### Hosts.

*Hypodynerusvespiformis* (Haliday, 1837), *Hypodyneruscoarctatus* (Saussure, 1852), *Monobiacingulata* Brèthes, 1903 (Teson and Remes Lenicov 1979).

##### Distribution.

Chile; Argentina: Salta (Teson and Remes Lenicov 1979).

#### ﻿ Leionotoxenos robertsoni

Taxon classificationAnimaliaStrepsipteraXenidae

(Pierce, 1911) comb. nov.

E44577C8-DE82-5980-9B61-E218B4DEBAD0

Pseudoxenos robertsoni Pierce, 1911: 501.

##### Host.

*Stenodynerushistrionalis* (Robertson, 1901) (as Odynerus (Ancistrocerus) histrionalis Robertson, 1901) (Pierce 1911).

##### Distribution.

USA: Illinois (Pierce 1911).

#### ﻿ Leionotoxenos tigridis

Taxon classificationAnimaliaStrepsipteraXenidae

(Pierce, 1911) comb. nov.

A168AA98-A9E2-5D2E-A088-0C5E4A4AEE07

Pseudoxenos tigridis Pierce, 1911: 501.

##### Host.

*Ancistrocerusadiabatus* (Saussure, 1853) (as Odynerus (Ancistrocerus) tigris Saussure, 1853) (Pierce 1911).

##### Distribution.

USA: Illinois (Pierce 1911).

#### ﻿ Leionotoxenos vigili

Taxon classificationAnimaliaStrepsipteraXenidae

(Brèthes, 1923) comb. nov.

FC0B952E-07E3-5856-8D92-CD1DA9524140

Montezumiaphila vigili Brèthes, 1923: 45.
Pseudoxenos vigili (Brèthes, 1923) (new combination by Kinzelbach 1971b).

##### Host.

*Montezumiabruchii* Brèthes, 1903 (as *Montezumiavigilii* Brèthes, 1910) (Brèthes 1923).

##### Distribution.

Argentina: Córdoba (Brèthes 1923); Venezuela (this study).

#### ﻿ Eupathocera

Taxon classificationAnimaliaStrepsipteraXenidae

Pierce, 1908, stat. res.

E6558DA6-90FC-5ACA-B5AA-9BF44A3461A0

Eupathocera Pierce, 1908: 79. Type species: Eupathoceralugubris Pierce, 1908, by original designation.
Pseudoxenos Saunders, 1872 (partim!) (synonymy proposed by Bohart 1937: 133).
Paraxenos Saunders, 1872 (partim!) (synonymy proposed by Kinzelbach 1971b: 162).
Homilops Pierce, 1908: 80 (syn. nov.). Type species: Xenoswestwoodii Templeton, 1838, by subsequent designation.
Sceliphronechthrus Pierce, 1909: 141 (syn. nov.). Type species: Sceliphronechthrusfasciati Pierce, 1909, by original designation.
Ophthalmochlus Pierce, 1909: 142 (syn. nov.). Type species: Ophthalmochlusduryi Pierce, 1909, by original designation.
Ophthalmochlus (Isodontiphila) Pierce, 1919: 465 (syn. nov.). Type species: Ophthalmochlusauripedis Pierce, 1911.

##### Diagnosis of female cephalothorax.

Differing from its sister genus *Leionotoxenos* by the shape of the periantennal area and the microstructure of the frontal area. Periantennal area expanded, sometimes raised, smooth (Fig. 29C). Distance between antennal area and supra-antennal sensillary field relatively large. Frontal region smooth or indistinctly wrinkled (Fig. 30B). Prosternum of most species of *Eupathocera* distinctly elevated above head medially and laterally, but apparently flat in *Leionotoxenos* (Fig. 29A). Rudiments of antennal torulus usually preserved (Fig. 29D). Sensilla restricted to clypeal lobe, not extended to ventral side of clypeal area. Mandible not protruding from capsule. In contrast to *Paragioxenos*, head and prothorax ventrally delimited by birth opening medially and by suture laterally.

##### Description of female cephalothorax.

**Figure 28.**
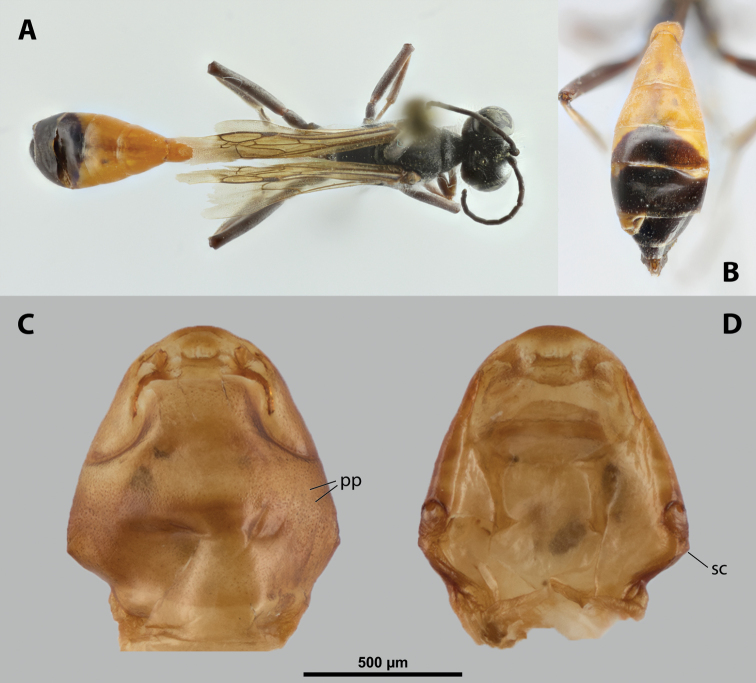
*Eupathoceraluctuosae* (Pierce), host, female, cephalothorax, photomicrographs **A***Ammophila* sp. stylopized by female of *E.luctuosae*, lateral view **B** detail of host abdomen with adult female inside **C** ventral side of cephalothorax **D** dorsal side of cephalothorax. Abbreviations: pp – pigmented papillae, sc – spiracular corner.

**Shape and coloration.** Compact, variable in shape, longer than wide to nearly as long as wide. Abdominal segment I sometimes protruding laterally, forming corner below spiracles (Fig. 28D). Very variable in size, length 1.02–2.47 mm, maximum width 0.88–2.5 mm. Anterior head margin evenly rounded or slightly protruding. Thorax slightly widening posteriorly. Coloration variable, with mostly dark or light brown pattern, but also patterns of multiple brown shades.

**Figure 29.**
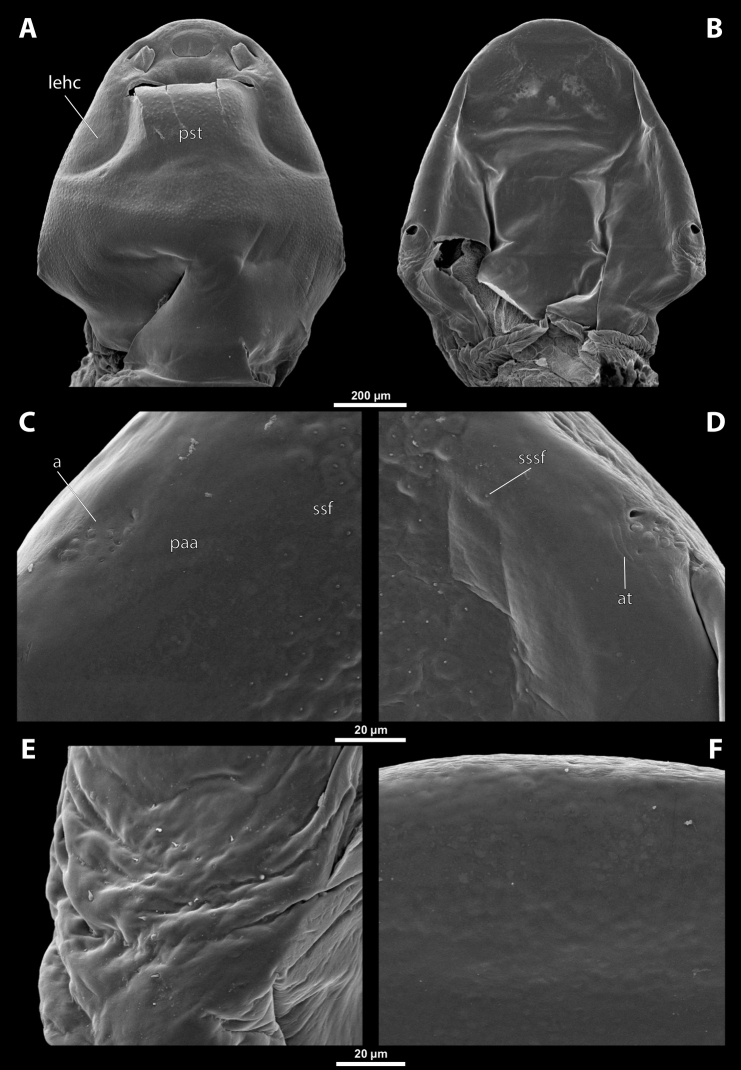
*Eupathoceraluctuosae* (Pierce), female, cephalothorax, SEM micrographs **A** ventral side **B** dorsal side **C** left vestigial antenna, dorsal side **D** right vestigial antenna, dorsal side **E** left lateral border of abdominal segment I below spiracle, dorsal side **F** detail of anterior border of cephalothorax, dorsal side. Abbreviations: a – vestigial antenna, at – antennal torulus, lehc – lateral extension of head capsule, paa – periantennal area, pst – prosternum, ssf – supra-antennal sensillary field, sssf – sensillum of supra-antennal sensillary field.

**Figure 30.**
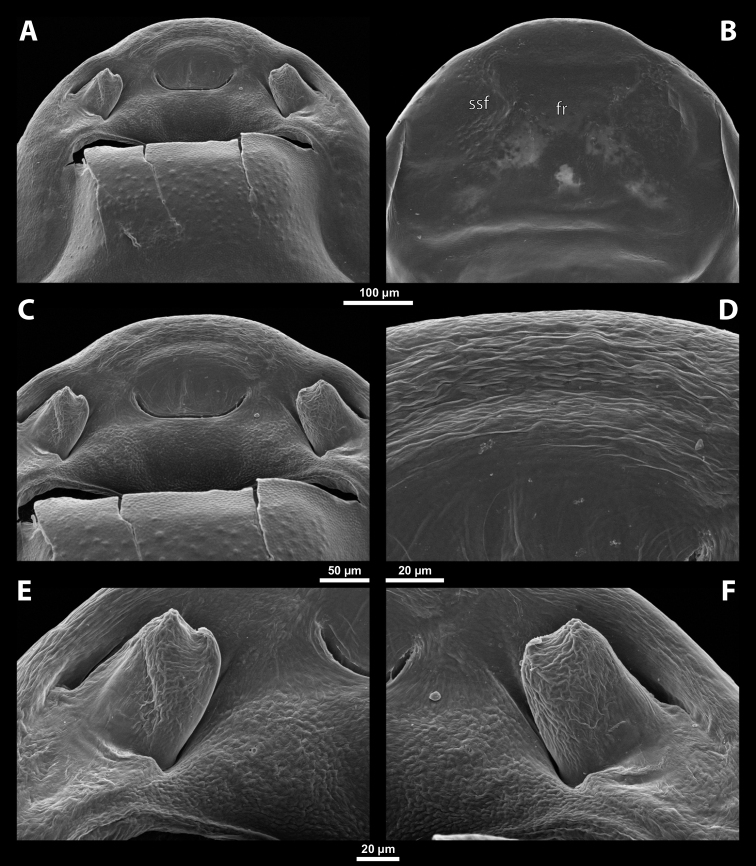
*Eupathoceraluctuosae* (Pierce), female, cephalothorax, SEM micrographs **A** anterior part of cephalothorax, ventral side **B** anterior part of cephalothorax, dorsal side **C** mouthparts, ventral side **D** detail of anterior border of cephalothorax, ventral side **E** right mandible and maxilla, ventral side **F** left mandible and maxilla, ventral side. Abbreviations: fr – frontal region, ssf – supra-antennal sensillary field.

**Head capsule.** Ca. ¼ ~ ⅖ as long as entire cephalothorax including lateral cephalic extensions. Coloration rather pale to dark or forming specific patterns. Clypeal area well defined or not well delimited from labral area, with indistinct or slightly protruding clypeal lobe. Surface varying from wrinkled, lamellar, with scarcely visible sensilla, to completely smooth with distinctly exposed sensilla. Number of clypeal sensilla 20–80 or even more. Border between clypeal and frontal region clearly recognizable or indistinct but still present. Frontal region smooth or indistinctly wrinkled (Fig. 30B). Segmental border between head and prothorax distinct or only faintly recognizable on dorsal side.

**Supra-antennal sensillary field.** Smooth or slightly wrinkled, with dispersed sensilla (Fig. 29D). Not distinctly delimited by furrow medially, but border marked by different surface structure of supra-antennal sensillary field and smooth frontal region (Fig. 30B).

**Antenna.** Preserved as more or less clearly defined area. Antennal torulus usually reduced, preserved as interrupted furrow (Fig. 29D). Periantennal area expanded, sometimes raised, smooth (Fig. 29C). Distance between antennal area and supra-antennal sensillary field relatively large.

**Labrum.** Ventral field wider than long, elliptic to nearly circular. Dorsal labral field slightly arcuate, at least 4× wider than long in midline. Setae on dorsal field conspicuous, ~ 10–22.

**Mandible.** Anteromedially directed at an angle of 30–55°, enclosed in mandibular capsule. Mandibular bulge more or less distinctly raised, with ~ 5 indistinct sensilla. Cuticle of mandible smooth with longitudinal grooves or sculptured. Mandibular tooth narrow or slightly widened, with or without spines.

**Maxilla.** Reduced and not distinctly protruding, not projecting beyond mandible anteriorly. Partially fused to labial area, both regions often not clearly separated. Cuticle wrinkled or reticulated, in some cases with smooth areas. Vestige of palp inconspicuous, forming small bulge, sometimes very indistinct, located anteriorly or medially on ventral side of maxilla. Submaxillary groove indistinctly produced posteriorly to maxillary base.

**Labium.** Labial area more or less distinctly recognizable between maxillae, flat, longer than wide in midline or as long as wide. Anteriorly delimited by mouth opening, posteriorly by birth opening. Cuticular surface smooth or slightly reticulated.

**Mouth opening.** More or less arcuate, sclerotized along margin.

**Thorax and abdominal segment I.** Pro-mesothoracic and meso-metathoracic borders variable, distinct or indistinct, usually indicated by mesal furrows, often combined with pigmented stripes. Border between metathorax and abdomen usually marked by change in cuticular surface structure or pigmentation. Cuticle of thoracic segments reticulate on ventral side, often with scattered small, pigmented papillae. Dorsal side of thorax smooth or slightly wrinkled. Prosternal extension undifferentiated, or anteriorly with specific color pattern. Prosternum distinctly elevated above head medially and laterally in most species (Fig. 29A). Shape of meso- and metathorax unmodified, transverse. Setae and cuticular spines present on lateral region of abdominal segment I (Fig. 29E).

**Spiracles.** Spiracles on posterior ~ ⅓ of cephalothorax slightly elevated, with anterolateral or anterodorsal orientation.

##### Diagnosis of male cephalotheca.

**Figure 31.**
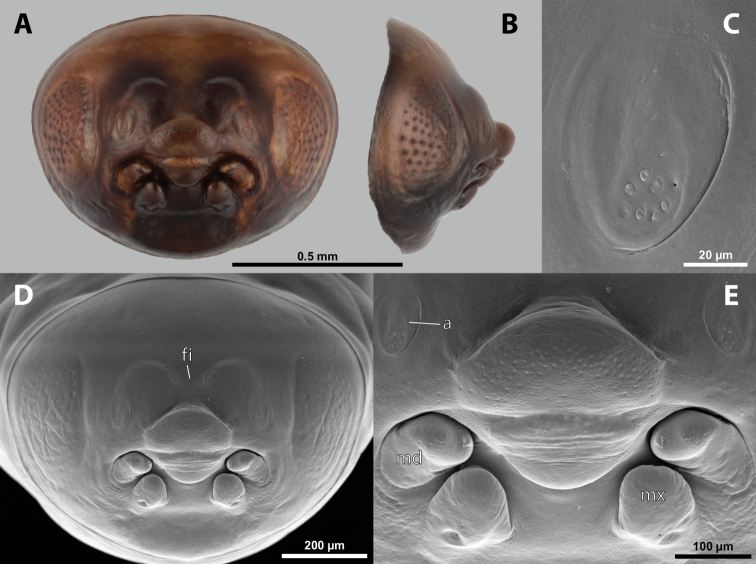
Eupathoceracf.inclusa (Oliveira & Kogan), male, cephalotheca, photomicrographs, SEM micrographs **A** frontal view **B** lateral view **C** vestigial antenna **D** frontal view **E** mouthparts. Abbreviations: a – vestigial antenna, fi – frontal impression, md – mandible, mx – vestige of maxilla.

Differing from other genera in the following characters. Diameter of genae between maxillary base and compound eye at least 2× as large as diameter of vestigial antenna. Paired furrow of supra-antennal sensillary field indistinct or absent. Cephalotheca usually of nearly circular shape (Fig. 31A). Antennal diameter ca. as long as width of mandible (Fig. 31E). Mandible directed anteromedially.

##### Description of male cephalotheca.

**Shape and coloration.** In frontal view rounded, nearly circular, in lateral view pointed anteriorly. Coloration with a pattern of dark and slightly paler shades.

**Cephalothecal capsule.** Compound eyes with dark individual ommatidia well visible on paler ocular background. Very conspicuous clypeal lobe straight in frontal view, prominent in lateral view, bulging. Sensilla mainly concentrated on clypeal lobe. Frontal impression indistinct. Occipital bulge absent. Diameter of genae between maxillary base and compound eye large, > 2× as large as diameter of vestigial antenna.

**Supra-antennal sensillary field.** Kidney-shaped and bulging, delimited medially by weakly developed frontal impression. Distinct furrows not visible.

**Vestigial antenna.** Of standard shape, small, sometimes with incomplete torulus, and with small plates or cavities (Fig. 31C). Periantennal area not clearly delimited from supra-antennal sensillary field.

**Labrum.** Labral area distinct, with setae on dorsal field.

**Mandible.** Anteromedially directed. Tooth pointed, not reaching area of mandibular bulge basally. Bulge with sensilla.

**Maxilla.** Distinct, prominent, completely dark. Vestige of palp distinct.

**Labium and hypopharynx.** Labium distinct between and below maxillae, dark. Praementum and postmentum indistinctly separated by furrow. Hypopharyngeal protuberance absent.

**Mouth opening.** Poorly visible, partially covered by ventral labral field, arcuate.

##### Phylogenetic relationships.

According to Benda et al. (2019) part of a clade of a New World origin, also containing *Leionotoxenos* Pierce.

##### Diversity and distribution.

Including 16 valid species, restricted to the New World.

##### Hosts.

Various wasps from three families, but mostly sphecids (Sphecidae: Sphecinae, Ammophilinae), rarely *Tachytes* (Crabronidae: Crabroninae) and *Zethus* (Vespidae: Zethinae).

##### Comments.

The genus *Eupathocera* was described by Pierce (1908) based on his concept that a new genus of Strepsiptera should be established if it utilizes a different host genus. The description of the male was too short and superficial. It was later synonymized with *Pseudoxenos* (Bohart 1937) and then with *Paraxenos* (Kinzelbach 1971b). We restore *Eupathocera* from synonymy and classify it as a valid genus, based on the molecular phylogeny (Benda et al. 2019, 2021) and morphological characters newly reported here. We classify the names *Ophthalmochlus*, Ophthalmochlus (Isodontiphila), *Homilops*, and *Sceliphronechthrus* as synonyms of *Eupathocera*. Based on morphological characters, species parasitising *Pachodynerus* (Vespidae) were assigned to *Eupathocera*.

### ﻿List of species

#### ﻿ Eupathocera argentina

Taxon classificationAnimaliaStrepsipteraXenidae

(Brèthes, 1923) comb. nov.

8C9983BB-0F15-5CCA-9875-4636C136C3FA

Ophthalmochlus (Homilops) argentinus Brèthes, 1923: 52.
Pseudoxenos argentinus (Brèthes, 1923) (new combination by Bohart 1937).
Paraxenos argentinus (Brèthes, 1923) (new combination by Kinzelbach 1971b).

##### Host.

*Prionyxthomae* (Fabricius, 1775) (as *Proterosphexplatensis* Brèthes, 1908) (Brèthes 1923).

##### Distribution.

Argentina: Buenos Aires (Brèthes 1923).

#### ﻿ Eupathocera auripedis

Taxon classificationAnimaliaStrepsipteraXenidae

(Pierce, 1911) comb. nov.

900B5B5B-02E8-593F-9BA1-8286B6A86398

Ophthalmochlus auripedis Pierce, 1911: 503.
Pseudoxenos auripedis (Pierce, 1911) (new combination by Bohart 1937).
Paraxenos auripedis (Pierce, 1911) (new combination by Kinzelbach 1971b).

##### Hosts.

*Isodontiaauripes* (Fernald, 1906) (Pierce 1911); *Isodontiamexicana* (Saussure, 1867) (Benda et al. 2021).

##### Distribution.

USA: Maryland (Pierce 1911).

#### ﻿ Eupathocera bucki

Taxon classificationAnimaliaStrepsipteraXenidae

(Trois, 1984a) comb. nov.

C121C253-382B-542E-9ACA-B5DF31BF75B6

Paraxenos bucki Trois, 1984a: 16.

##### Host.

*Ammophila* sp. (Trois 1984a).

##### Distribution.

Brazil (Trois 1984a).

#### ﻿ Eupathocera duryi

Taxon classificationAnimaliaStrepsipteraXenidae

(Pierce, 1909) comb. nov.

71510CDE-A38C-5433-A0E6-646A331BF422

Ophthalmochlus duryi Pierce, 1909: 142.
Ophthalmochlus duryi Pierce, 1908: nomen nudum.
Pseudoxenos duryi (Pierce, 1909) (new combination by Bohart 1937).
Paraxenos duryi (Pierce, 1909) (new combination by Kinzelbach 1971b).

##### Host.

*Prionyxatratus* (Lepeletier, 1845) (as *Priononyxatrata* Lepeletier, 1845) (Pierce 1909).

##### Distribution.

USA: Ohio (Pierce 1909).

#### ﻿ Eupathocera erynnidis

Taxon classificationAnimaliaStrepsipteraXenidae

(Pierce, 1911) comb. nov.

82BB8F2A-CECD-5DCA-A822-6DEEAEAFD3BE

Pseudoxenos erynnidis Pierce, 1911: 499.

##### Host.

*Pachodyneruserynnis* (Lepeletier, 1941) (as *Odyneruserynnys* Lepeletier, 1941) (Pierce 1911).

##### Distribution.

USA: Florida (Pierce 1911), Colorado (this study).

##### Note.

This species has an lineage with unclear phylogenetic position (Benda et al. 2021). It is provisionally assigned to *Eupathocera* based on morphological characters. A more comprehensive sampling and a detailed study are necessary for a reliable classification of this taxon.

#### ﻿ Eupathocera fasciati

Taxon classificationAnimaliaStrepsipteraXenidae

(Pierce, 1909) comb. nov.

767C0DEA-1225-5010-A75F-CBEDC0C0F1E8

Sceliphronechthrus fasciati Pierce, 1909: 141.
Pseudoxenos fasciati (Pierce, 1909) (new combination by Bohart 1937).
Paraxenos fasciati (Pierce, 1909) (new combination by Kinzelbach 1971b).

##### Host.

*Sceliphronfasciatum* (Lepeletier, 1845) (as Sceliphron (Pelopaeus) fasciatus Lepeletier, 1845) (Pierce 1909).

##### Distribution.

Dominican Republic: Santo Domingo (Pierce 1909).

#### ﻿ Eupathocera fuliginosi

Taxon classificationAnimaliaStrepsipteraXenidae

(Brèthes, 1923) comb. nov.

0A3F1C2B-3F5B-5594-91E1-27253E883870

Ophthalmochlus (Homilops) fuliginosi Brèthes, 1923: 49.
Pseudoxenos fuliginosi (Brèthes, 1923) (synonymy proposed by Bohart 1937).
Paraxenos fuliginosi (Brèthes, 1923) (synonymy proposed by Kinzelbach 1971b).

##### Hosts.

*Sphexservillei* Lepeletier, 1845 (as *Proterosphexfuliginosus* Dahlbom, 1843) (Brèthes 1923); *Sphexargentinus* Taschenberg, 1869 (Benda et al. 2021).

##### Distribution.

Argentina: Tucumán (Brèthes 1923).

#### ﻿ Eupathocera inclusa

Taxon classificationAnimaliaStrepsipteraXenidae

(Oliveira & Kogan, 1963) comb. nov.

27AFD85D-710C-5934-A44B-35B04AD2D0D4

Pseudoxenus inclusus Oliveira & Kogan, 1963: 351.
Paraxenos inclusus (Brèthes, 1923) (new combination by Kinzelbach 1971b).

##### Host.

*Ammophila* sp. (Oliveira and Kogan 1963).

##### Distribution.

Brazil: Espírito Santo (Oliveira and Kogan 1963).

#### ﻿ Eupathocera insularis

Taxon classificationAnimaliaStrepsipteraXenidae

(Kifune, 1983) comb. nov.

1E59D4B4-5DE5-5C6F-AB57-206514D946CC

Pseudoxenos insularis Kifune, 1983: 335.

##### Host.

*Pachodyneruscinerascens* (Fabricius, 1775) (Kifune 1983).

##### Distribution.

Virgin Islands (Kifune 1983).

##### Note.

As *Eupathoceraerynnidis* this species has an unclear phylogenetic position (Benda et al. 2021). It is also provisionally included in the genus *Eupathocera* Pierce, 1908, stat. res. based on morphological evidence.

#### ﻿ Eupathocera luctuosae

Taxon classificationAnimaliaStrepsipteraXenidae

Pierce, 1911, stat. res.

BC81A18C-64AB-5EFE-839D-2DE54D996F2A

Eupathocera luctuosae Pierce, 1911: 502.
Pseudoxenos luctuosae (Brèthes, 1923) (new combination by Bohart 1937).
Paraxenos luctuosae (Brèthes, 1923) (new combination by Kinzelbach 1971b).

##### Hosts.

*Podalonialuctuosa* (F. Smith, 1856) (as Sphex (Psammophila) luctuosa F. Smith, 1856) (Pierce 1911); *Podaloniaargentifrons* (Cresson, 1865); *Podaloniaviolaceipennis* (Lepeletier, 1845) (Kinzelbach 1971b).

##### Distribution.

USA: Idaho, Colorado (Pierce 1911).

#### ﻿ Eupathocera lugubris

Taxon classificationAnimaliaStrepsipteraXenidae

Pierce, 1909, stat. res.

C2787D22-3B72-5182-80B4-BF6F141FCEB3

Eupathocera lugubris Pierce, 1909: 143.
Eupathocera lugubris Pierce, 1908: nomen nudum
Paraxenos lugubris (Pierce, 1908) (new combination by Kinzelbach 1971b).
Eupathocera pruinosae Pierce, 1909 (synonymized by Bohart 1941).
Eupathocera pictipennidis Pierce, 1911 (synonymized by Bohart 1941).
Eupathocera vulgaridis Pierce, 1911 (synonymized by Bohart 1941).

##### Hosts.

*Ammophilaaberti* Haldeman, 1852 (as *Sphextransversus* Ferdanand, 1934); *Ammophilaarvensis* Lepeletier, 1845 (as *Sphexarvensis* (Dahlbom, 1843)); *Ammophilabreviceps* F. Smith, 1856 (= *Sphexbreviceps* (F. Smith, 1856)); *Ammophilaextremitata* Cresson, 1865; *Ammophilafernaldi* (Murray, 1938); *Ammophilagracilis* Lepeletier, 1845 (as Sphex (Ammophila) fragilis (F. Smith, 1856)); *Ammophilakennedyi* (Murray, 1938) (as Sphex (Ammophila) vulgaris (Cresson, 1865)); *Ammophilanasalis* Provancher, 1895 (as *Sphexcraspedotus* Fernald, 1934 and *S.nasalis* (Provancher, 1895)); *Ammophilapictipennis* Walsh, 1869 (as Sphex (Ammophila) pictipennis (Walsh, 1869)); *Ammophilapruinosa* Cresson, 1865 (as Sphex (Ammophila) pruinosa (Cresson, 1865)); *Ammophilaurnaria* Dahlbom, 1843 (as *Sphexurnarius* (Dahlbom, 1843)); *Eremnophilaaureonotata* (Cameron, 1888) (as *Sphexaureonotatus* (Cameron, 1888)) (Pierce 1909; Bohart 1941; Kathirithamby et al. 2012; Cook 2019).

##### Distribution.

USA: Ohio, Colorado, Illinois, Iowa (Pierce 1909; Bohart 1941; Cook 2019).

#### ﻿ Eupathocera mendozae

Taxon classificationAnimaliaStrepsipteraXenidae

(Brèthes, 1923) comb. nov.

D07299E9-8BCD-5F5E-86E7-B8243B4C0215

Ophthalmochlus (Homilops) mendozae Brèthes, 1923: 51.
Pseudoxenos mendozae (Brèthes, 1923) (new combination by Bohart 1937).
Paraxenos mendozae (Brèthes, 1923) (new combination by Kinzelbach 1971b).

##### Host.

*Prionyxneoxenus* (Kohl, 1890) (as *Priononyxneoxenus*, var. melanogaster Brèthes, 1910) (Brèthes 1923).

##### Distribution.

Argentina: Mendoza (Brèthes 1923).

#### ﻿ Eupathocera piercei

Taxon classificationAnimaliaStrepsipteraXenidae

(Brèthes, 1923) comb. nov.

AD153BA2-3ABB-5488-8C85-92F10C739E18

Ophthalmochlus (Homilops) piercei Brèthes, 1923: 50.
Pseudoxenos piercei (Brèthes, 1923) (new combination by Bohart 1937).
Paraxenos piercei (Brèthes, 1923) (new combination by Kinzelbach 1971b).

##### Host.

*Isodontiacostipennis* (Spinola, 1851) (Brèthes 1923).

##### Distribution.

Argentina: La Rioja (Brèthes 1923).

#### ﻿ Eupathocera striati

Taxon classificationAnimaliaStrepsipteraXenidae

(Brèthes, 1923) comb. nov.

EF128D46-BF72-5D2C-8B23-9AE90508006C

Ophthalmochlus (Homilops) Brèthes, 1923: 48.
Pseudoxenos striati (Brèthes, 1923) (new combination by Bohart 1937).
Paraxenos striati (Brèthes, 1923) (new combination by Kinzelbach 1971b).

##### Host.

*Prionyxfervens* (Linnaeus, 1758) (as *Priononyxstriatus* F. Smith, 1856) (Brèthes 1923).

##### Distribution.

Argentina: Córdoba (Brèthes 1923).

#### ﻿ Eupathocera taschenbergi

Taxon classificationAnimaliaStrepsipteraXenidae

(Brèthes, 1923) comb. nov.

F6A6BD53-49AB-5E4F-827C-6792A59E3C63

Ophthalmochlus (Homilops) taschenbergi Brèthes, 1923: 47.
Pseudoxenos taschenbergi (Brèthes, 1923) (new combination by Bohart 1937).
Paraxenos taschenbergi (Brèthes, 1923) (new combination by Kinzelbach 1971b).

##### Host.

*Prionyxpumilio* (Taschenberg, 1869) (as *Neosphexpumilio* (Taschenberg, 1869) (Brèthes 1923).

##### Distribution.

Argentina: Mendoza (Brèthes 1923).

#### ﻿ Eupathocera westwoodii

Taxon classificationAnimaliaStrepsipteraXenidae

(Templeton, 1841) comb. nov.

5427CA0B-3671-558E-8D7A-327D0C86B415

Xenos westwoodii Templeton, 1841: 53.
Pseudoxenos westwoodii (Templeton, 1841) (new combination by Bohart 1937).
Paraxenos westwoodii (Templeton, 1841) (new combination by Kinzelbach 1971b).
Paraxenos westwoodi (incorrect subsequent spelling): Kinzelbach (1971b).
Xenos smithii Heyden, 1867 (synonymized by Kinzelbach 1971b).
Homilops ashmeadi Pierce, 1909 (synonymized by Kinzelbach 1971b).
Pseudoxenos ashmeadi (Pierce, 1909) (new combination by Bohart 1937).
Homilops bishoppi Pierce, 1909 (synonymized by Kinzelbach 1971b).
Pseudoxenos bishoppi (Pierce, 1909) (new combination by Bohart 1937).

##### Hosts.

*Sphexichneumoneus* (Linnaeus, 1758) (as *Sphexaurocapillus* Templeton, 1841; *Sphexichneumoneusaurifluus* Perty, 1838; Proterosphex (Sphex) ichneumoneus Linnaeus, 1758); unknown name (Proterosphex (Sphex) pernanus Kohl) (Pierce 1909); *Sphexpensylvanicus* Linnaeus, 1763 (Miller et al. 2010); *Tachytes* sp. (this study).

##### Distribution.

Brazil: Rio de Janeiro (Templeton 1841); Dominican Republic: Santo Domingo; USA: Texas, Montana (Pierce 1909; Miller et al. 2010); Mexico (this study).

#### ﻿ Macroxenos

Taxon classificationAnimaliaStrepsipteraXenidae

Schultze, 1925, stat. res.

CE4A08BB-FB94-5E69-BC46-E97B9D430985

Macroxenos Schultze, 1925: 238. Type species: Macroxenospiercei Schultze, 1925, by original designation.
Pseudoxenos Saunders, 1872 (partim!) (synonymy proposed by Bohart 1937).

##### Diagnosis of female cephalothorax.

**Figure 32.**
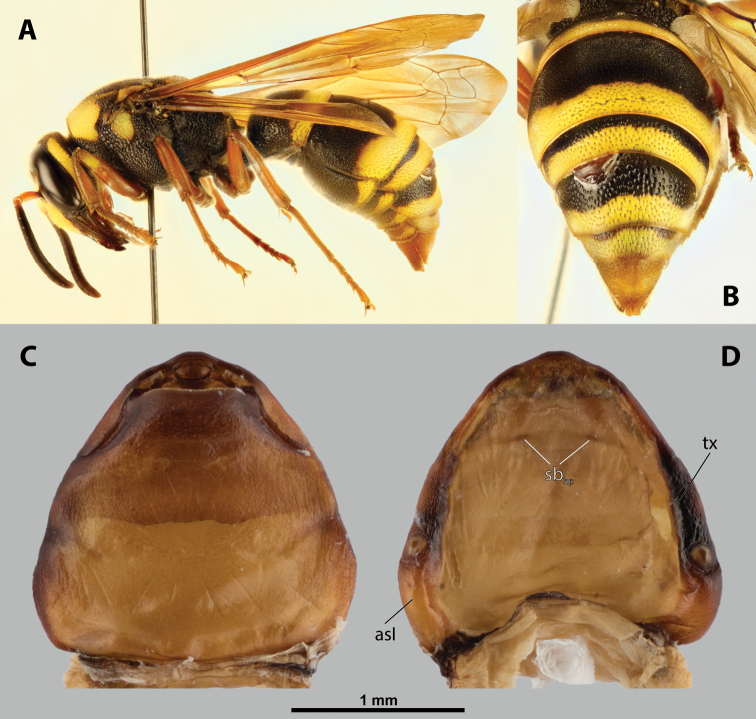
Macroxenoscf.piercei, host, female, cephalothorax, photomicrographs **A***Anterhynchiumflavomarginatum* stylopized by female of M.cf.piercei, lateral view **B** detail of host abdomen with adult female inside **C** ventral side of cephalothorax **D** dorsal side of cephalothorax. Abbreviations: asI – abdominal segment I, sbhp – segmental border between head and prothorax, tx – thorax.

Maxilla reduced, not distinctly prominent (Fig. 34E). Two distinct dark spots present mesally on border between head and prothorax (Fig. 32D). Thoracic segments conspicuously sclerotized laterally from dorsal side (Fig. 32D). Lateral parts of abdomen posterior to spiracles always pale (Fig. 32D). Clypeal region bulging, very distinctly separated from labral area (Fig. 34D). Mandible not protruding from capsule. In contrast to *Paragioxenos*, head and prothorax ventrally delimited by birth opening medially and by suture laterally.

##### Description of female cephalothorax.

**Shape and coloration.** Nearly as long as wide, or as long as or distinctly longer than wide. Very variable in size, length 0.8–1.82 mm, width 0.64–1.9 mm in midline. Anterior head margin evenly rounded or protruding. Thorax slightly or distinctly widening posteriorly. Cephalothorax with multiple brown shades forming distinct pattern.

**Head capsule.** Between ⅓ and > ½ × as long as entire cephalothorax including the lateral cephalic extensions. Coloration forming specific pattern with pale and dark shades. Clypeal region very distinctly delimited from labral area (Fig. 34D), arcuate, or protruding and forming clypeal lobe. Surface smooth or distinctly wrinkled. Sensilla mainly concentrated on clypeal lobe. Border between clypeal area and frontal region clearly indicated by change in cuticular surface. Cuticle of frontal area variable, distinctly wrinkled or covered with papillae. Border between head and prothorax usually distinct on dorsal side, delimited by transverse stripe of distinctive coloration and two distinct dark spots on mesal region (Fig. 32D).

**Supra-antennal sensillary field.** Smooth, with dispersed sensilla. Furrow between supra-antennal sensillary field and frontal region absent, or very indistinct and only indicated by change in cuticular sculpture (Fig. 34B).

**Antenna.** Preserved as poorly defined area, with several small, rounded plates, antennal sensilla, or cavity, in some cases all three combined. Periantennal area smooth or slightly wrinkled, sometimes indistinct.

**Labrum.** Ventral field wider than long, elliptic to nearly circular. Dorsal field arcuate, distinctly raised (Fig. 34D), sometimes very wide and narrow, ~ 5–8× wider than long in midline. Dorsal field with 14–41 (or more) setae or sensilla inserted in cavities.

**Mandible.** Anteromedially directed at angle of 30–35° and enclosed in mandibular capsule. Mandibular bulge distinctly raised, with several sensilla. Cuticle smooth or slightly sculptured, sometimes with longitudinal grooves (Fig. 34E). Tooth narrow or slightly widened, pointed apically or ventrally, more or less distinctly armed with spines.

**Maxilla.** Almost completely fused with labial area, or slightly raised (Fig. 34E), not projecting beyond mandible. Cuticle smooth or wrinkled. Vestige of palp present as cavity or poorly defined area; usually located medially on ventral side of maxilla (Fig. 34E). Submaxillary groove more or less distinctly produced anterolaterally to maxillary base.

**Labium.** Labial area between maxillae usually more or less distinct, delimited anteriorly by mouth opening and posteriorly by birth opening. Labial area wider than long in midline, flat or convex. Cuticular surface smooth or reticulated.

**Mouth opening.** Widely arcuate, sclerotized marginally.

**Figure 33.**
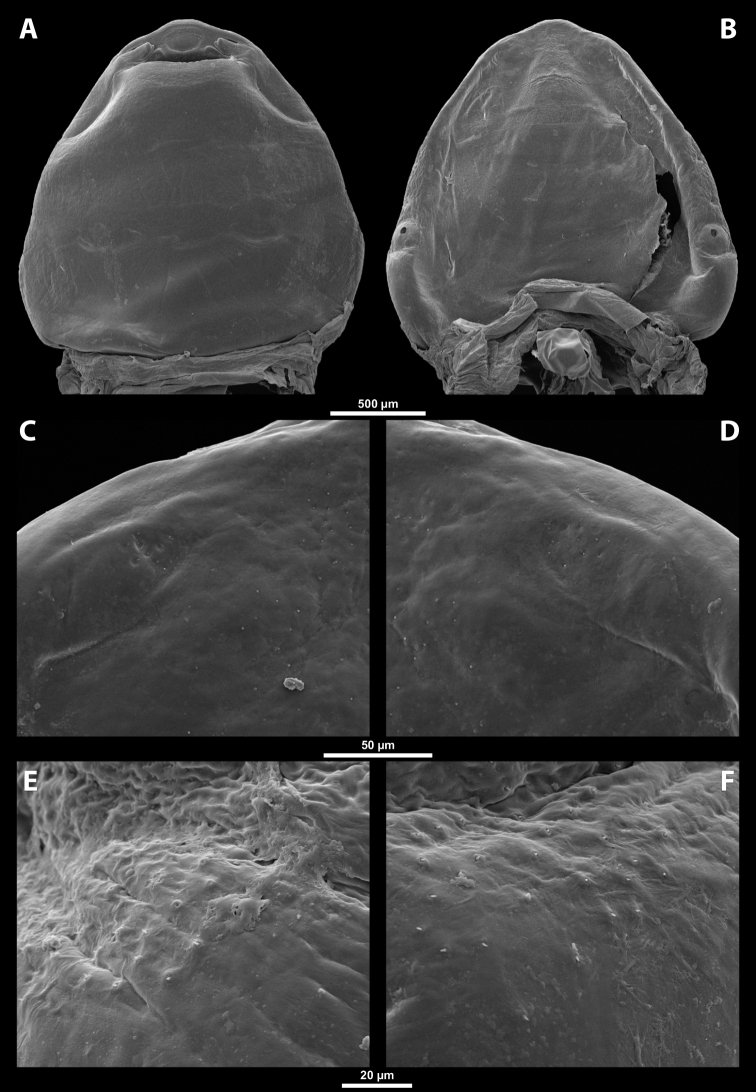
Macroxenoscf.piercei, female, cephalothorax, SEM micrographs **A** ventral side **B** dorsal side **C** left vestigial antenna, dorsal side **D** right vestigial antenna, dorsal side **E** left lateral border of abdominal segment I below spiracle, dorsal side **F** right lateral border of abdominal segment I below spiracle, dorsal side.

**Thorax and abdominal segment I.** Pro-mesothoracic and meso-metathoracic borders more or less distinct, usually separated by mesal furrows, rarely combined with pigmented stripes or spots on dorsal and ventral side. Border between metathorax and abdomen usually formed by ridge or indicated by change in cuticular sculpture. Cuticle of thoracic segments on ventral side reticulate, with scattered inconspicuous or more distinct pigmented papillae. Dorsal surface of thorax smooth or slightly reticulated. Prosternal extension undifferentiated or distinct, in some cases extremely elongated. Thoracic segments conspicuously sclerotized laterally from dorsal side (Fig. 32D). Shape of meso- and metathorax unmodified, transverse, or narrowed laterally in species with elongated head. Lateral parts of abdomen posterior to spiracles always pale (Fig. 32D). Setae present on lateral region of abdominal segment I (Fig. 33E, F).

**Figure 34.**
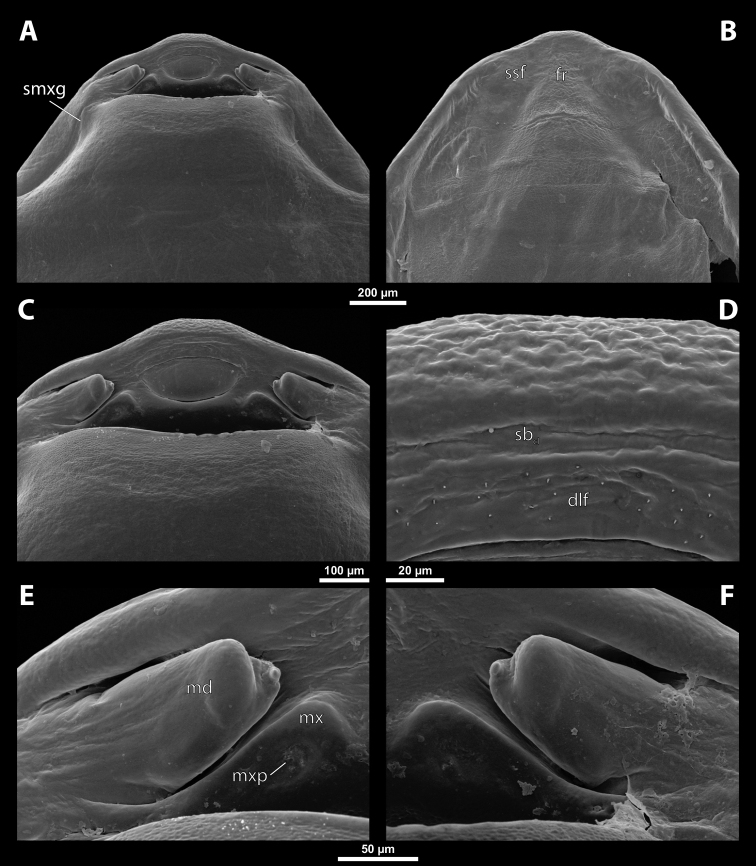
Macroxenoscf.piercei, female, cephalothorax, SEM micrographs **A** anterior part of cephalothorax, ventral side **B** anterior part of cephalothorax, dorsal side **C** mouthparts, ventral side **D** detail of anterior border of cephalothorax, ventral side **E** right mandible and maxilla, ventral side **F** left mandible and maxilla, ventral side. Abbreviations: dlf – dorsal field of labral area, fr – frontal region, md – mandible, mx – vestige of maxilla, mxp – vestige of maxillary palp, sbcl – segmental border between clypeus and labrum, smxg – submaxillary groove, ssf – supra-antennal sensillary field.

**Spiracles.** Spiracles on posterior ~ ⅓ of cephalothorax slightly elevated, with anterodorsal and anterolateral orientation.

##### Diagnosis of male cephalotheca.

Male cephalotheca unknown.

##### Phylogenetic relationships.

The phylogenetic position is unstable. Benda et al. (2019) revealed it as sister to a lineage including *Sphecixenos*, *Tuberoxenos*, and *Pseudoxenos* in our concept. In contrast, Benda et al. (2021) resolved its position as sister to a clade including *Sphecixenos*, *Tuberoxenos*, *Pseudoxenos*, *Deltoxenos*, and *Xenos*. In both cases, the support was very weak. Further phylogenomic investigations with robust data are needed to resolve the intergeneric relationships.

##### Diversity and distribution.

A lineage of Australasian origin, with dispersion into the Indomalayan region (Benda et al. 2019). The two currently known species are restricted to these two biogeographic regions.

##### Hosts.

Various genera of Odynerini (Vespidae: Eumeninae).

##### Comments.

The genus *Macroxenos* was described by Schultze (1925) but the descriptions of male and female was superficial. Later, Bohart (1937) synonymized it with *Pseudoxenos*. We classify this lineage as a separate genus, based on molecular phylogenies (Benda et al. 2019, 2021) and morphological characters newly reported here. However, this genus is quite complicated to diagnose because of a high morphological variability of species. More samples are still needed for a better characterization and recognition of this formerly overlooked group.

### ﻿List of species

#### ﻿ Macroxenos papuanus

Taxon classificationAnimaliaStrepsipteraXenidae

(Székessy, 1956) comb. nov.

D5ED8B90-55D8-5247-A242-8F790B5BDCD5

Pseudoxenos papuanus Székessy, 1956: 149.

##### Host.

*Allodynerusfloricola* (Saussure, 1852) (as *Odynerusfloricola* Saussure) (Székessy 1956).

##### Distribution.

New Guinea (Székessy 1956).

##### Note.

The occurrence of *Allodynerus* in New Guinea is unlikely. Host identity thus requires a confirmation. Although only *Macroxenos* is known from the Australasian region as parasitic lineage of Odynerini wasps, we decided to assign this species to this genus preliminarily, pending a more detailed study in the future.

#### ﻿ Macroxenos piercei

Taxon classificationAnimaliaStrepsipteraXenidae

Schultze, 1925, stat. res.

9A3A227A-D4BA-5529-BD66-A01A587A5381

Macroxenos piercei Schultze, 1925: 238.
Pseudoxenos piercei (Schultze, 1925) (new combination by Bohart 1937).
Pseudoxenos schultzei Kifune & Maeta, 1965: 7 (synonymized by Kinzalbach 1971a).

##### Host.

*Rhynchiumatrum* Saussure, 1852 (Schultze 1925); *Rhynchiumatrissimum* Vecht, 1968 (Kifune and Tano 1991).

##### Distribution.

Philippines: Luzon (Schultze 1925), Mindanao (Kifune and Tano 1991).

##### Note.

Kifune and Maeta (1965) proposed a new replacement name for *Macroxenospiercei* Schultze, 1925, a secondary homonym of *Ophthalmochluspiercei* Brèthes, 1923 (now *Eupathocerapiercei* (Brèthes, 1923), comb. nov.) when both were placed in the same genus *Pseudoxenos*. *Macroxenospiercei* is reinstated here as a valid name following the Article 59.4 of ICZN (1999).

#### ﻿ Sphecixenos gen. nov.

Taxon classificationAnimaliaStrepsipteraXenidae

148FFB8C-B648-5760-AF07-41AE56EE44B1

http://zoobank.org/B5D80275-0542-40D3-B4F4-DB229A6DDDDD

##### Type species.

*Paraxenosorientalis* Kifune, 1985, here designated.

##### Diagnosis of female cephalothorax.

**Figure 35.**
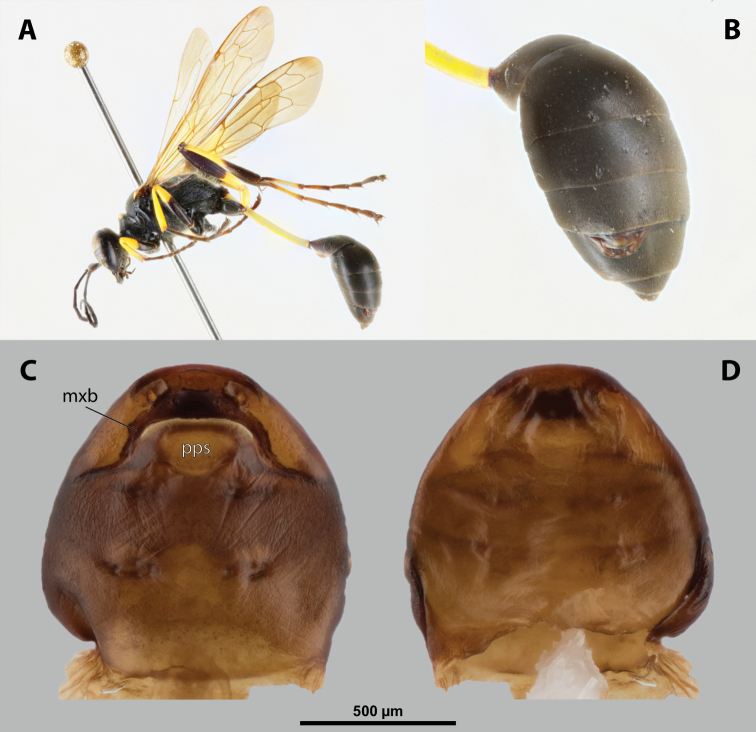
*Sphecixenosorientalis*, host, female, cephalothorax, photomicrographs **A***Sceliphronmadraspatanum* stylopized by female of *S.orientalis*, lateral view **B** detail of host abdomen with adult female inside **C** ventral side of cephalothorax **D** dorsal side of cephalothorax. Abbreviations: mxb – maxillary base, pps – prosternal pale spot.

Differing from all other genera of Xenidae by very distinct prosternal features: prosternal extension anteriorly with very conspicuous, extensive pale spot, sometimes associated with cuticular impression (Figs 35C, 37A). A feature linked with the maxillae is shared with *Paraxenos* or *Tuberoxenos*: submaxillary groove distinctly produced posterolaterally to maxillary base (Fig. 37A), extending along cephalic border distally and then connected to border between head and prothorax. In contrast to *Paragioxenos*, head and prothorax ventrally delimited by birth opening medially and by suture laterally.

##### Description of female cephalothorax.

**Shape and coloration.** Compact, ca. as long as wide, or slightly longer. Size variable, length 0.96–1.64 mm, maximum width 0.9–1.8 mm. Anterior head margin rounded, not protruding. Thorax slightly widening posteriorly. Abdominal segment I sometimes protruding laterally, forming rounded corner below spiracles. Coloration never completely pale, comprising multiple brown shades forming distinct patterns.

**Head capsule.** ~ ⅓ ~ ⅖ as long as entire cephalothorax including lateral cephalic extensions. Combination of pale and dark brown shades resulting in specific color pattern. Clypeal region well delimited from labral area, arcuate, without or with slightly protruding clypeal lobe. Surface smooth or slightly wrinkled. Sensilla (> 30) better visible in dorsal view than ventrally, concentrated mainly on anterior clypeal area. Border between clypeal region and frontal area indistinctly recognizable. Frontal area smooth or slightly reticulated. Dorsal border between head and prothorax indicated by interrupted suture or distinctive coloration, or scarcely recognizable.

**Supra-antennal sensillary field.** Smooth or slightly wrinkled, with evenly dispersed sensilla, not delimited or indistinctly delimited by furrow medially (Fig. 37B).

**Figure 36.**
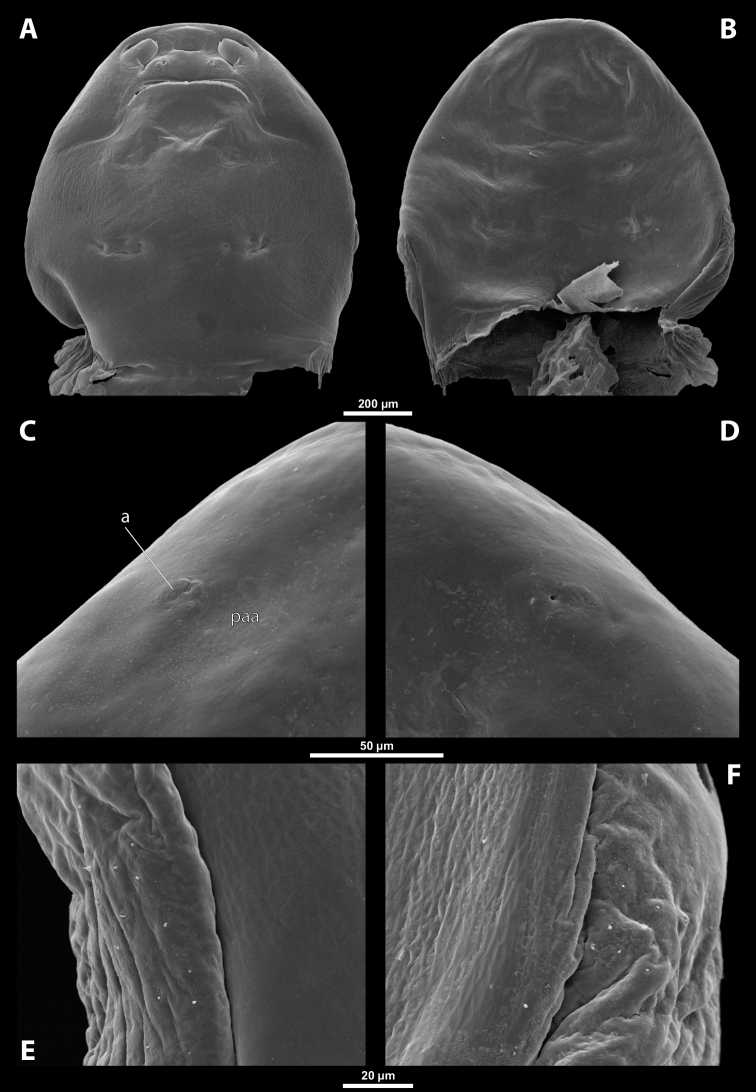
*Sphecixenosorientalis*, female, cephalothorax, SEM micrographs **A** ventral side **B** dorsal side **C** left vestigial antenna, dorsal side **D** right vestigial antenna, dorsal side **E** left lateral border of abdominal segment I below spiracle, dorsal side **F** right lateral border of abdominal segment I below spiracle, dorsal side. Abbreviations: a – vestigial antenna, paa – periantennal area.

**Antenna.** Preserved as poorly defined area with several small, rounded plates, cavity, or sensilla. Periantennal area smooth (Fig. 36C).

**Labrum.** Ventral field wider than long, elliptic. Dorsal field slightly arcuate, 3–4× wider than long in midline. Dorsal field with several inconspicuous setae, usually blunt, not pointed.

**Mandible.** Mandibles anteromedially directed at angle of 35–55°, enclosed in mandibular capsule. Mandibular bulge rounded or pointed, with several sensilla. Cuticle smooth, with longitudinal grooves. Tooth narrow, armed with spines.

**Figure 37.**
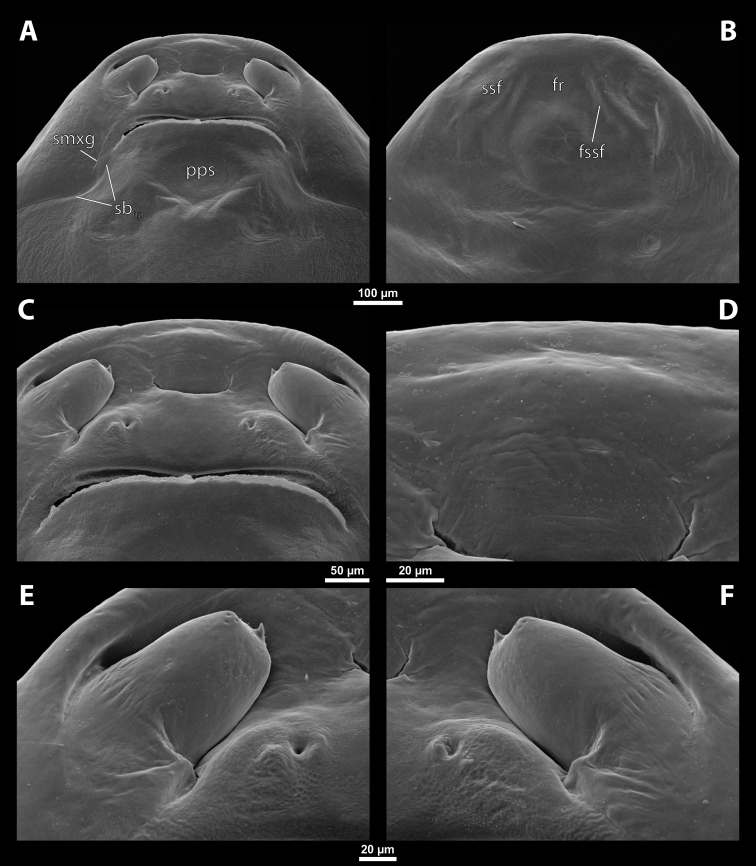
*Sphecixenosorientalis*, female, cephalothorax, SEM micrographs **A** anterior part of cephalothorax, ventral side **B** anterior part of cephalothorax, dorsal side **C** mouthparts, ventral side **D** detail of anterior border of cephalothorax, ventral side **E** right mandible and maxilla, ventral side **F** left mandible and maxilla, ventral side. Abbreviations: fr – frontal region, fssf – furrow of supra-antennal sensillary field, pps – prosternal pale spot, sbhp – segmental border between head and prothorax, smxg – submaxillary groove, ssf – supra-antennal sensillary field.

**Maxilla.** Variable in shape, in some cases reduced and fused to labium, otherwise well-developed, separated from labial area, anteriorly directed, prominent but not projecting beyond mandible. Cuticle finely reticulated. Vestige of palp present as cavity with accessory plates or reduced. Submaxillary groove distinctly produced posterolaterally to maxillary base extending along cephalic border (Fig. 37A).

**Labium.** Labial area between maxillae flat but distinct, delimited anteriorly by mouth opening and posteriorly by birth opening. Wider than long in midline or as long as wide. Cuticular surface smooth or reticulated.

**Mouth opening.** Distinctly arcuate to nearly straight, sclerotized marginally.

**Thorax and abdominal segment I.** Pro-mesothoracic and meso-metathoracic borders relatively distinct, indicated by mesal furrows combined with stripes of specific coloration. Border between metathorax and abdomen usually indicated by change in cuticular surface structure or pigmentation. Cuticle of thoracic segments on ventral side reticulate with scattered small and pigmented papillae. Cuticle of dorsal side of thorax indistinctly reticulated. Prosternal extension differentiated anteriorly, with very conspicuous extensive pale spot, sometimes associated with cuticular impression (Figs 35C, 37A). Shape of meso- and metathorax unmodified, transverse. Setae on lateral region of abdominal segment I (Fig. 36E, F) present, or cuticular surface distinctly sculptured.

**Spiracles.** Spiracles on posterior third of cephalothorax slightly elevated, with lateral or anterolateral orientation.

##### Diagnosis of male cephalotheca.

**Figure 38.**
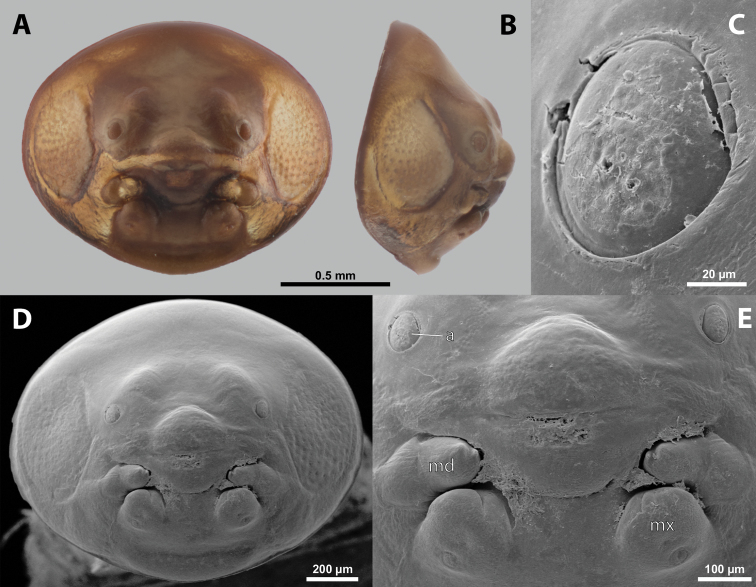
Sphecixenoscf.gigas, male, cephalotheca, photomicrographs, SEM micrographs **A** frontal view **B** lateral view **C** vestigial antenna **D** frontal view **E** mouthparts. Abbreviations: a – vestigial antenna, md – mandible, mx – vestige of maxilla.

Differing from other genera by large diameter of genae between maxillary base and compound eye, at least 2× as large as diameter of vestigial antenna. Distinct paired furrow of supra-antennal sensillary field absent. Cephalotheca nearly circular in frontal view (Fig. 38A). Diameter of vestigial antennae smaller than width of medially directed mandible (Fig. 38E).

##### Description of male cephalotheca.

**Shape and coloration.** In frontal view rounded, nearly circular, in lateral view rounded or slightly pointed anteriorly. With pattern of multiple shades of brown.

**Cephalothecal capsule.** Compound eyes with dark individual ommatidia well visible on paler ocular background. Clypeal lobe straight in frontal view, slightly protruding in lateral view. Sensilla mainly concentrated on medial clypeal region. Frontal impression indistinct. Occipital bulge absent. Diameter of genae between maxillary base and compound eye large, > 2× as large as diameter of vestigial antenna.

**Supra-antennal sensillary field.** Kidney-shaped and bulging, distinctly developed. Lacking distinct furrows medially.

**Antenna.** Of standard shape but very small, with small plates or cavities and complete torulus (Fig. 38C). Periantennal area not clearly delimited from supra-antennal sensillary field.

**Labrum.** Labral area distinct.

**Mandible.** Rather medially directed than anteromedially. Mandibular tooth pointed, not reaching area of mandibular bulge basally.

**Maxilla.** Distinct, prominent, with entirely dark coloration. Vestige of palp distinct.

**Labium and hypopharynx.** Dark labium distinct between and below maxillae. Praementum and postmentum separated by furrow. Hypopharyngeal protuberance not present.

**Mouth opening.** Clearly visible, not covered by ventral labral field, slightly arcuate.

##### Phylogenetic relationships.

According to Benda et al. (2019, 2021) sister to a monophyletic lineage containing *Pseudoxenos* and *Tuberoxenos* gen. nov.

##### Diversity and distribution.

This genus represents a lineage of Afrotropical origin which dispersed to Australia (Benda et al. 2019). It currently comprises 12 species, distributed in the Old World (mainly Afrotropical and Oriental regions) and Australian region.

##### Hosts.

*Sphex*, *Isodontia* (Sphecidae: Sphecinae), *Sceliphron* (Sphecidae: Sceliphrinae), and *Chlorion* (Sphecidae: Chloriontinae).

##### Etymology.

The name is derived from the family Sphecidae, the only known host family of this genus. The ending -*xenos* is used in several generic names, mainly in the family Xenidae. It is from a Greek substantive meaning enemy or stranger. Gender masculine.

##### Comments.

All described species of *Sphecixenos* gen. nov. were previously placed in *Paraxenos* based on parasitising digger wasps (Kinzelbach 1971b). Despite this concept, this group is morphologically well defined. We classify it as a separate genus, based on the molecular phylogeny (Benda et al. 2019, 2021) and morphological characters newly reported here.

### ﻿List of species

#### ﻿ Sphecixenos abbotti

Taxon classificationAnimaliaStrepsipteraXenidae

(Pierce, 1909) comb. nov.

38381182-5A07-52C0-AAE9-F1613B6F3461

Homilops abbotti Pierce, 1909: 147.
Pseudoxenos abbotti (Pierce, 1909) (new combination by Bohart 1937).
Paraxenos abbotti (Pierce, 1909) (new combination by Kinzelbach 1971b).

##### Host.

*Sphex* sp. (as *Proterosphex* sp.) (Pierce 1909).

##### Distribution.

Thailand: Trang (Pierce 1909).

#### ﻿ Sphecixenos astrolabensis

Taxon classificationAnimaliaStrepsipteraXenidae

(Székessy, 1956) comb. nov.

45314E4F-3084-532B-A617-875428D59FE3

Pseudoxenos astrolabensis Székessy, 1956: 144.
Paraxenos astrolabensis (Székessy, 1956) (new combination by Kinzelbach 1971b).

##### Host.

*Sphexcognatus* F. Smith, 1856 (as *Sphexformosus* F. Smith, 1856) (Székessy 1956).

##### Distribution.

New Guinea: New Britain (Székessy 1956).

#### ﻿ Sphecixenos dorae

Taxon classificationAnimaliaStrepsipteraXenidae

(Luna de Carvalho, 1956) comb. nov.

482A9A49-40D2-5E54-89E1-7BDD1B64225E

Pseudoxenos dorae Luna de Carvalho, 1956: 41.
Paraxenos dorae (Luna de Carvalho, 1956) (new combination by Kinzelbach 1971b).

##### Hosts.

*Chlorion* sp. (Luna de Carvalho 1956); *Sphexnigrohirtus* Kohl, 1895 (Kinzelbach 1971b).

##### Distribution.

Angola (Luna de Carvalho 1956).

#### ﻿ Sphecixenos erimae

Taxon classificationAnimaliaStrepsipteraXenidae

(Székessy, 1956) comb. nov.

35555114-3BD5-58D8-AC0A-33F696BED1B7

Pseudoxenos erimae Székessy, 1956: 146.
Paraxenos erimae (Saunders, 1872) (new combination by Kinzelbach 1971b).

##### Host.

*Sphexfumicatus* Christ, 1791 (as *Sphexmetallicus* Taschenberg, 1869) (Székessy 1956).

##### Distribution.

New Guinea (Székessy 1956).

#### ﻿ Sphecixenos esakii

Taxon classificationAnimaliaStrepsipteraXenidae

(Hirashima & Kifune, 1962) comb. nov.

05AEDF50-C02C-591C-87DA-848F5AA311CF

Pseudoxenos esakii Hirashima & Kifune, 1962: 175.
Paraxenos esakii (Hirashima & Kifune, 1962) (new combination by Kinzelbach 1971b).

##### Hosts.

*Isodontiamaidli* (Yasumatsu, 1938) (Kifune and Tano 1985); *Isodontianigella* (F. Smith, 1856) (as *Sphexnigellus* F. Smith, 1856) (Hirashima and Kifune 1962).

##### Distribution.

Japan (Hirashima and Kifune 1962).

#### ﻿ Sphecixenos gigas

Taxon classificationAnimaliaStrepsipteraXenidae

(Pasteels, 1950) comb. nov.

08452F56-8450-5732-82E0-96564B57CFC3

Pseudoxenos gigas Pasteels, 1950: 290.
Paraxenos gigas (Pasteels, 1950) (new combination by Kinzelbach 1971b).

##### Hosts.

*Sphexlanatus* Mocsáry, 1883; *Sphexargentatus* Fabricius, 1787 (as *Sphexumbrosus* Christ, 1791); *Sphexfumicatus* Christ, 1791 (as *Sphexmetallicus* Taschenberg, 1869); *Sphexschoutedeni* Kohl, 1913 (as Isodontia (Proterosphex) schoutedeni Kohl, 1913); *Isodontiastanleyi* (Kohl, 1890) (as *Sphexstanleyi* Kohl, 1890) (Pasteels 1950; Kinzelbach 1971b).

##### Distribution.

Democratic Republic of Congo (Pasteels 1950).

#### ﻿ Sphecixenos kurosawai

Taxon classificationAnimaliaStrepsipteraXenidae

(Kifune, 1984) comb. nov.

1C3987B5-3140-5054-B4C2-05FB77B257EE

Paraxenos kurosawai Kifune, 1984: 87.

##### Host.

*Sphexmadasummae* Vecht, 1973 (Kifune 1984).

##### Distribution.

Philippines: Palawan (Kifune 1984).

#### ﻿ Sphecixenos laetus

Taxon classificationAnimaliaStrepsipteraXenidae

(Ogloblin, 1926) comb. nov.

46789D60-A3D0-5F67-83C6-A10FA27A52C1

Sceliphronechthrus laetum Ogloblin, 1926: 133.
Pseudoxenos laetum (Saunders, 1872) (new combination by Bohart, 1937).
Paraxenos laetum (Saunders, 1872) (new combination by Kinzelbach 1971b).

##### Host.

*Sceliphronlaetum* (Smith, 1856).

##### Distribution.

New Guinea; Australia: Queensland (Ogloblin 1926).

##### Note.

According to the article 34.2.1 of ICZN (1999), the ending of species name was adjusted to the grammatical gender of the new genus.

#### ﻿ Sphecixenos orientalis

Taxon classificationAnimaliaStrepsipteraXenidae

(Kifune, 1985) comb. nov.

53551F19-5D37-58A0-A574-9CF211DAE351

Paraxenos orientalis Kifune in Kifune & Yamane, 1985: 52.

##### Host.

*Sceliphronmadraspatanumformosanum* Vecht, 1968 (Kifune and Yamane 1985).

##### Distribution.

Japan: Iriomote and Ishigaki islands (Kifune and Yamane 1985); Laos; Thailand (this study).

#### ﻿ Sphecixenos reticulatus

Taxon classificationAnimaliaStrepsipteraXenidae

(Luna de Carvalho, 1972) comb. nov.

98CFBAE7-DF56-5322-BA77-9251460D4984

Paraxenos reticulatus Luna de Carvalho, 1972: 136.

##### Host.

*Sphextomentosus* Fabricius, 1787 (as *Sphextuberculatum* F. Smith, 1873) (Luna de Carvalho 1972).

##### Distribution.

Angola: Dundo (Luna de Carvalho 1972).

#### ﻿ Sphecixenos simplex

Taxon classificationAnimaliaStrepsipteraXenidae

(Székessy, 1956) comb. nov.

6F660C7F-AB4C-5FD9-A68A-36947941C8CE

Pseudoxenos simplex Székessy, 1956: 145.
Paraxenos simplex (Székessy, 1956) (new combination by Kinzelbach 1971b).

##### Host.

*Isodontiapraslinia* (Guérin-Méneville, 1831) (as *Sphexsimplex* Kohl, 1898) (Székessy 1956).

##### Distribution.

New Guinea (Székessy 1956).

#### ﻿ Sphecixenos vanderiisti

Taxon classificationAnimaliaStrepsipteraXenidae

(Pasteels, 1952) comb. nov.

F277EAFF-526A-56B5-8755-95203515D186

Pseudoxenos vanderiisti Pasteels, 1952: 252.
Paraxenos vanderiisti (Pasteels, 1952) (new combination by Kinzelbach 1971b).

##### Host.

*Isodontiapelopoeiformis* (Dahlbom, 1845) (as Chlorion (Isodontia) pelopaeiformis, Gerstaecker) (Pasteels 1952).

##### Distribution.

Democratic Republic of Congo (Pasteels 1952).

##### Note.

Pasteels (1952) probably misspelled the host name and the author of its description. Kinzelbach (1971b) probably overlooked these mistakes. We adjust it in accordance with Cook (2019).

#### ﻿ Pseudoxenos

Taxon classificationAnimaliaStrepsipteraXenidae

Saunders, 1872

AF90B145-A30D-532B-AB55-7499256C1D5B

Pseudoxenos Saunders, 1872: 44. Type species: Pseudoxenosschaumii Saunders, 1872, by original designation.

##### Diagnosis of female cephalothorax.

**Figure 39.**
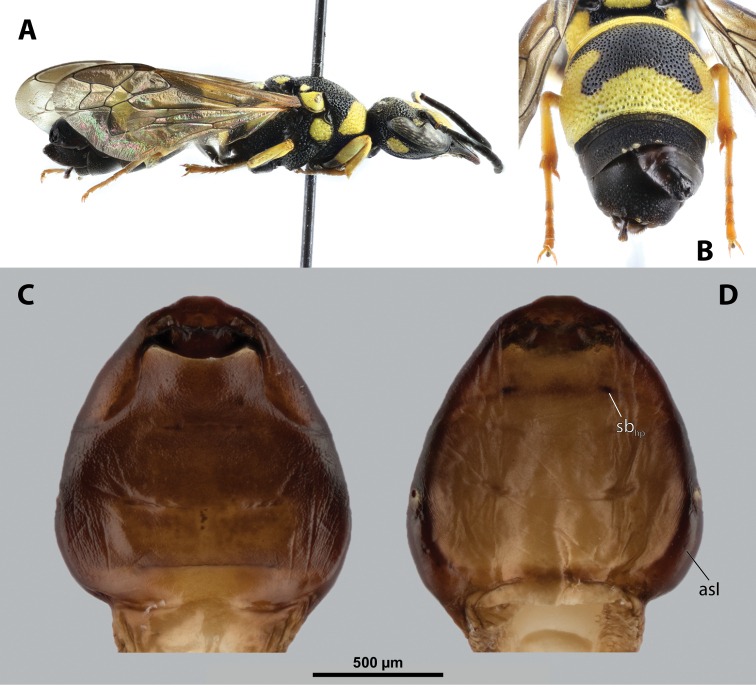
*Pseudoxenos* sp., host, male, female, cephalothorax, photomicrographs **A***Paradontodynerus* sp. stylopized by male of *Pseudoxenos* sp., lateral view **B** detail of host abdomen with male puparium inside **C** ventral side of female cephalothorax **D** dorsal side of female cephalothorax. Abbreviations: asI – abdominal segment I, sbhp – segmental border between head and prothorax.

Differs from *Tuberoxenos* by flat dorsal field of labrum (Fig. 41C) and more flattened cephalothorax, with more or less even shape (Fig. 39C), appearing flattened-elliptical in cross section. Distinguished from *Deltoxenos* by dorsal labral field laterally as long as along midline (Fig. 41C, D), and meso-metathoracic segmental border not constricted laterally. In contrast to *Macroxenos* lateral parts of abdomen posterior to spiracles with dark coloration (Fig. 39D). Mandible nested in mandibular capsule. In contrast to *Paragioxenos*, head and prothorax ventrally delimited by the birth opening in middle region and laterally by a suture.

##### Description of female cephalothorax.

**Shape and coloration.** Compact, longer than wide, elliptic in cross-section. Meso-metathoracic segmental border not constricted laterally. Size fairly constant, length 1.08–1.44 mm, maximum width 1.02–1.4 mm. Anterior head margin rounded or protruding. Thorax slightly widening posteriorly. Coloration with multiple brown shades forming pattern.

**Figure 40.**
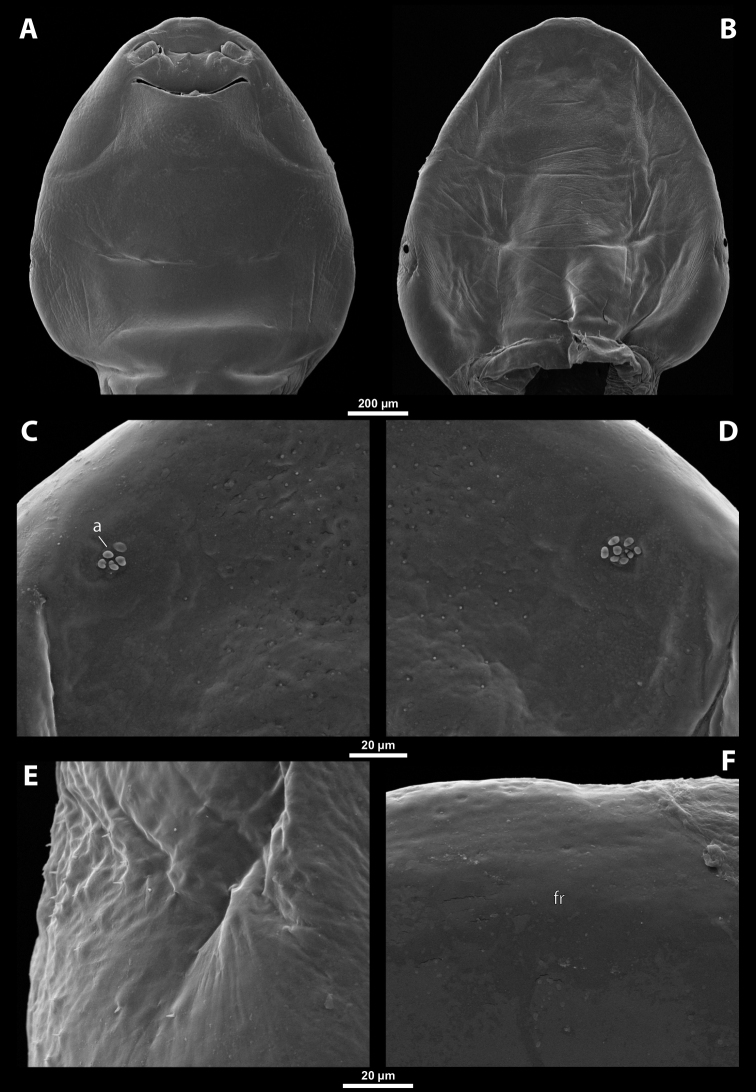
*Pseudoxenos* sp., female, cephalothorax, SEM micrographs **A** ventral side **B** dorsal side **C** left vestigial antenna, dorsal side **D** right vestigial antenna, dorsal side **E** left lateral border of abdominal segment I below spiracle, dorsal side **F** detail of anterior border of cephalothorax, dorsal side. Abbreviations: a – vestigial antenna, fr – frontal region.

**Figure 41.**
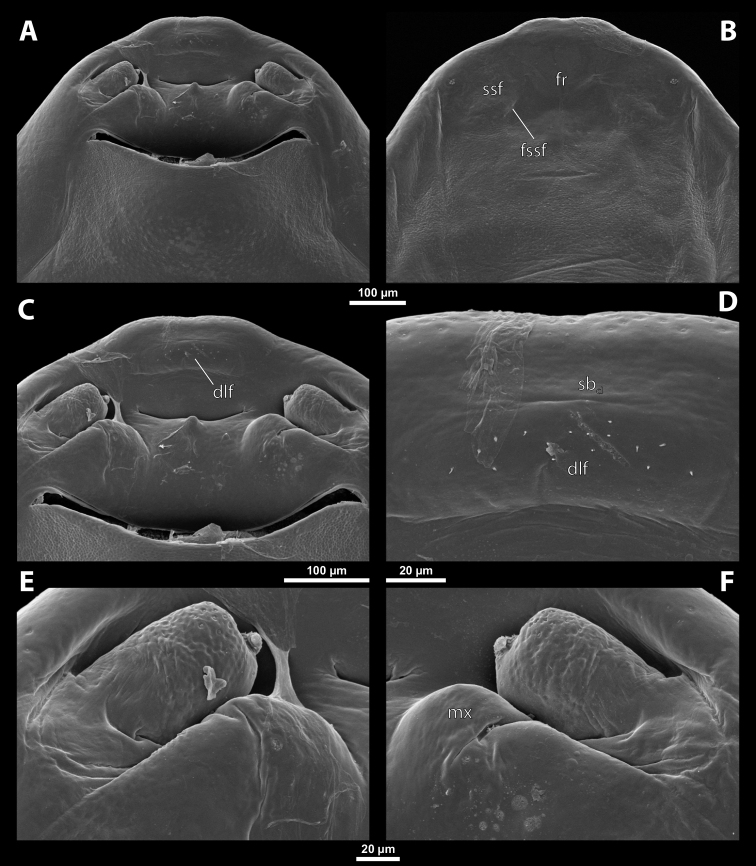
*Pseudoxenos* sp., female, cephalothorax, SEM micrographs **A** anterior part of cephalothorax, ventral side **B** anterior part of cephalothorax, dorsal side **C** mouthparts, ventral side **D** detail of anterior border of cephalothorax, ventral side **E** right mandible and maxilla, ventral side **F** left mandible and maxilla, ventral side. Abbreviations: dlf – dorsal field of labral area, fr – frontal region, fssf – furrow of supra-antennal sensillary field, mx – vestige of maxilla, sbhp – segmental border between head and prothorax, ssf – supra-antennal sensillary field.

**Head capsule.** Ca. ⅖ as long as entire cephalothorax including lateral extensions. Coloration mostly dark brown, often with specific patterns. Clypeal region delimited from labral area (Fig. 41D), arcuate, or protruding and forming clypeal lobe. Surface smooth or slightly wrinkled. Approximately 35–56 sensilla mainly concentrated anteriorly but dispersed over entire clypeal area. Border between clypeal area and frontal region hardly distinct but still recognizable. Frontal surface smooth (Fig. 40F). Segmental border between head and prothorax clearly recognizable or indistinct on dorsal side, often indicated by dark brown stripes, and in some cases with two distinct dark spots on mesal region (Fig. 39D).

**Supra-antennal sensillary field.** Smooth or slightly wrinkled, with dispersed sensilla. Furrow forming border on medial side more or less distinct (Fig. 41B).

**Antenna.** Preserved as poorly defined area, sometimes raised, usually with several small, rounded plates, rarely with additional sensilla or cavity (Fig. 40C). Periantennal area smooth.

**Labrum.** Ventral field distinctly wider than long, elliptic. Dorsal field nearly straight, slightly arcuate, at least 4–5× wider than long in midline, flat and smooth, with 15–21 clearly visible setae inserted in cavities (Fig. 41C, D). Dorsal field laterally as long as medially, in some cases almost merging with head capsule.

**Mandible.** Mandibles anteromedially directed at an angle of 35–45° and nested in mandibular capsule. Mandibular bulge not or slightly raised, bears several sensilla. Cuticle of mandible sculptured to nearly smooth. Mandibular tooth narrow, pointed, straight or hook-shaped, armed with spines.

**Maxilla.** Separated from labial area, slightly or distinctly protruding, prominent portion directed anteriorly or anterolaterally, maxilla slightly overlapping with mandible proximally (Fig. 41F), but not projecting beyond it anteriorly. Cuticle usually smooth, rarely wrinkled. Vestige of palp very distinct, with more or less distinct plates or cavity, located medially on ventral side of maxilla. Submaxillary groove more or less distinctly produced posterolaterally to maxillary base.

**Labium.** Labial area between maxillae flat but distinct, relatively large, delimited anteriorly by mouth opening and posteriorly by birth opening. As long as wide or longer than wide. Cuticular surface in most cases largely smooth and shiny, or faintly and uniformly sculptured.

**Mouth opening.** Mouth opening arcuate, nearly straight, or bi-arcuate, sclerotized marginally.

**Thorax and abdominal segment I.** Pro-mesothoracic and meso-metathoracic borders more or less distinct, separated by mesal furrows. Border between metathorax and abdomen formed by ridge. Cuticle of thoracic segments on ventral side reticulate with scattered small and pigmented papillae. Cuticle of dorsal side of thorax smooth or slightly wrinkled. Prosternal extension undifferentiated, anterior margin evenly arched. Meso- and metathorax transverse. Lateral parts of abdomen posterior to spiracle dark (Fig. 39D). Setae present on lateral region of abdominal segment I.

**Spiracles.** Spiracles on posterior ⅓ of cephalothorax slightly elevated, with anterolateral or lateral orientation.

##### Diagnosis of male cephalotheca.

**Figure 42.**
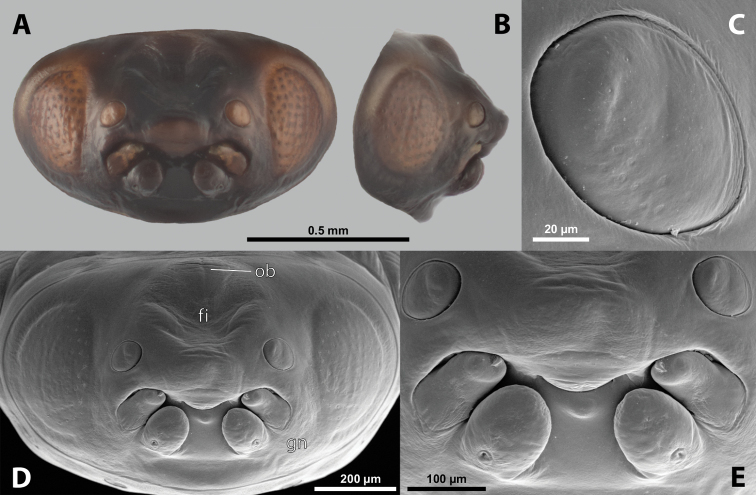
*Pseudoxenos* sp., male, cephalotheca, photomicrographs, SEM micrographs **A** frontal view **B** lateral view **C** vestigial antenna **D** frontal view **E** mouthparts. Abbreviations: fi – frontal impression, ob – occipital bulge.

Diameter of genae between maxillary base and compound eye ~ 1.5× as large as diameter of vestigial antenna. Occipital bulge present (Fig. 42D). Frontal region very distinctly deformed by frontal impression (Fig. 42D). Distinct paired furrows of supra-antennal sensillary field absent.

##### Description of male cephalotheca.

**Shape and coloration.** In frontal view rounded laterally, flattened, elliptical, in lateral view pointed anteriorly. Coloration with pattern of pale and dark shades.

**Cephalothecal capsule.** Compound eyes with darker individual ommatidia well visible on pale ocular background. Clypeal lobe straight or slightly arcuate in frontal view, prominent in lateral view. Sensilla mainly concentrated medially. Frontal impression distinctly present (Fig. 42D). Occipital bulge present (Fig. 42D). Diameter of genae between maxillary base and compound eye small, ~ 1.5× diameter of vestigial antenna.

**Supra-antennal sensillary field.** Kidney-shaped and bulging, delimited medially by frontal impression, without distinct furrows.

**Antenna.** Vestiges large, with complete torulus. Periantennal area not clearly delimited from supra-antennal sensillary field. Small plates or sensilla present (Fig. 27C).

**Labrum.** Labral area distinct, with setae on dorsal field.

**Mandible.** Anteromedially directed. Mandibular bulge with sensilla, separated from pointed tooth.

**Maxilla.** Distinct, prominent. Coloration completely dark. Vestige of palp distinct.

**Labium and hypopharynx.** Labium distinct between and below maxillae, dark. Praementum and postmentum distinctly separated by furrow. Hypopharyngeal protuberance present.

**Mouth opening.** Well visible, not covered by ventral labral field, slightly arcuate.

##### Phylogenetic relationships.

Deeply nested within Xenidae (Benda et al. 2019, 2021), part of a clade of an Old Word origin, with *Tuberoxenos* gen. nov. as sister group.

##### Diversity and distribution.

A group of Palearctic origin (Benda et al. 2019), comprising seven currently valid species restricted to this region.

##### Hosts.

Various genera of Odynerini (Vespidae: Eumeninae).

##### Comments.

*Pseudoxenos* was described by Saunders (1872) but only a superficial description of the male was provided. Bohart (1937) synonymized many names previously designed (*Eupathocera*, *Ophthalmochlus*, *Homilops*, *Leionotoxenos Sceliphronecthrus*, *Macroxenos*) with *Pseudoxenos*. Although later Kinzelbach (1971b) used *Pseudoxenos* for all xenids parasitising solitary Vespidae worldwide, the genus corresponds to a Palearctic clade utilizing Odynerini according to the molecular phylogeny of Benda et al. (2019, 2021). We classify this lineage as a separate genus, based on these molecular phylogenic works and morphological characters newly reported here.

### ﻿List of species

Only hosts from original descriptions are included. As the phylogeny of this genus is not clarified we do not present any other host species from later studies. The actual extent of morphological variation within and between species in Europe has not been assessed yet (Cook 2019). A more comprehensive sampling and a detailed taxonomic revision are necessary for a clarification of interspecific relationships and individual species concepts.

#### ﻿ Pseudoxenos andradei

Taxon classificationAnimaliaStrepsipteraXenidae

Luna de Carvalho, 1953

7720DBBA-6E27-5B53-9EA3-D9A275948BEC

Pseudoxenos andradei Luna de Carvalho, 1953: 3.
Pseudoxenos heydenii (Saunders, 1852) (partim!) (synonymy proposed by Kinzelbach 1978).

##### Host.

*Ancistrocerustriphaleratus* (Saussure, 1855) (Luna de Carvalho 1953).

##### Distribution.

Portugal: Vale do Gaio (Luna de Carvalho 1953).

#### ﻿ Pseudoxenos atlanticus

Taxon classificationAnimaliaStrepsipteraXenidae

Luna de Carvalho, 1969

1C82F8F6-895B-5AEE-A8E3-F91DC911E1BF

Pseudoxenos atlanticus Luna de Carvalho, 1969: 9.
Pseudoxenos heydenii (Saunders, 1852) (partim!) (synonymy proposed by Kinzelbach 1978).

##### Host.

*Odynerus* sp. (Luna de Carvalho 1969).

##### Distribution.

Portugal: Madeira isl., Funchal (Luna de Carvalho 1969).

#### ﻿ Pseudoxenos corcyricus

Taxon classificationAnimaliaStrepsipteraXenidae

(Saunders, 1872)

0B5320EE-B0E4-5361-931D-57A88000097A

Paraxenos corcyricus Saunders, 1872: 46.
Pseudoxenos corcyricus (Saunders, 1872) (new combination by Pierce 1909).
Pseudoxenos heydenii (Saunders, 1852) (partim!) (synonymy proposed by Kinzelbach 1978).

##### Host.

*Odynerusspinipes* (Linaeus, 1758) (Saunders 1872).

##### Distribution.

Greece: Corfu (Saunders 1872).

#### ﻿ Pseudoxenos heydenii

Taxon classificationAnimaliaStrepsipteraXenidae

(Saunders, 1852)

F2951028-6DD5-53F7-AB1F-E0B17A990071

Xenos heydenii Saunders, 1852: 141.
Pseudoxenos heydenii (Saunders, 1852) (new combination by Saunders 1872).
Pseudoxenos heydeni (incorrect subsequent spelling): Kinzelbach (1971b).
Pseudoxenos heydeni (incorrect subsequent spelling): Kinzelbach (1978).

##### Hosts.

*Antepiponadeflenda* (Saunders, 1853) (as *Ancistrocerusdeflendus*, Saunders, 1853).

##### Distribution.

Greece: Preveza, Epirus reg., Ambracian Gulf (Saunders 1852).

#### ﻿ Pseudoxenos klugii

Taxon classificationAnimaliaStrepsipteraXenidae

(Saunders, 1852)

C779F2AD-5D61-5D20-AD0D-1782DFC94B3B

Xenos klugii Saunders, 1852: 142.
Pseudoxenos klugii (Saunders, 1852) (new combination by Saunders 1872).
Pseudoxenos klugi (incorrect subsequent spelling): Kinzelbach (1971b).
Pseudoxenos heydenii (Saunders, 1852) (partim!) (synonymy proposed by Kinzelbach 1978).

##### Host.

*Gymnomeruslaevipes* (Shuckard, 1837) (as *Odynerusrubicola* Dufour, 1839) (Saunders 1852).

##### Distribution.

Greece: Preveza (Saunders 1852).

#### ﻿ Pseudoxenos seyrigi

Taxon classificationAnimaliaStrepsipteraXenidae

Monod, 1925

159814CC-E0AC-53DF-81D3-06D129E115F9

Pseudoxenos seyrigi Monod, 1925: 230.
Pseudoxenos heydenii (Saunders, 1852) (partim!) (synonymy proposed by Kinzelbach 1978).

##### Host.

*Euodynerusvariegatus* (Fabricius, 1793) (as *Odyneruscrenatus* Lepeletier, 1841) (Monod 1925).

##### Distribution.

Spain: Sierra Morena (Monod 1925).

#### ﻿ Pseudoxenos schaumii

Taxon classificationAnimaliaStrepsipteraXenidae

Saunders, 1872

78677CE0-D1EE-5FB1-959A-8E907BDD1FFC

Pseudoxenos schaumii Saunders, 1872: 44.
Pseudoxenos schaumi (incorrect subsequent spelling): Kinzelbach (1971b).
Pseudoxenos heydenii (Saunders, 1852) (partim!) (synonymy proposed by Kinzelbach 1978).

##### Host.

*Ancistrocerusparietum* (Linnaeus, 1758) (as *Odynerusparietum* Linnaeus, 1758) (Saunders 1872).

##### Distribution.

Greece: Corfu (Saunders 1872).

#### ﻿ Tuberoxenos gen. nov.

Taxon classificationAnimaliaStrepsipteraXenidae

15356098-BBD5-5C09-9917-50F497D92C96

http://zoobank.org/99152C5A-B0FE-47A3-85B7-2A3F5ED548DA

##### Type species.

*Xenossphecidarum* Siebold, 1839, here designated.

##### Diagnosis of female cephalothorax.

**Figure 43.**
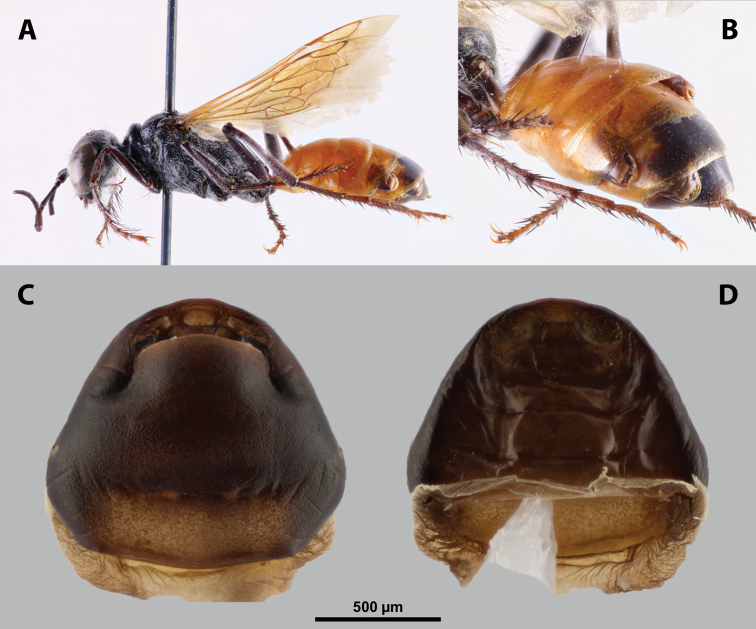
*Tuberoxenossphecidarum*, host, male, female, cephalothorax, photomicrographs **A***Podaloniatydei* stylopized by females of *T.sphecidarum*, lateral view **B** detail of host abdomen with three adult females inside **C** ventral side of female cephalothorax **D** dorsal side of female cephalothorax.

Distinguished from *Pseudoxenos* by conspicuously convex, round cephalothorax (Fig. 43C), and distinctly raised, anteriorly protruding dorsal labral field (Fig. 45D). Differring from other genera by the following combination of characters. Maxilla well-developed and clearly separated from labial area, prominent and directed anteriorly (Fig. 45E). Mandibular tooth narrow or slightly widened. Prosternal extension undifferentiated, evenly arched but in some cases protruding and overlapping with maxillolabial area and posterior part of mandibles. Differing from *Nipponoxenos* by mandible nested in capsule. In contrast to *Paragioxenos*, head and prothorax ventrally delimited by birth opening medially and by suture laterally.

##### Description of female cephalothorax.

**Shape and coloration.** Compact, ca. as long as wide or longer than wide. In ventral view appearing conspicuously convex, rotund (Fig. 43C), high-elliptic in cross-section. Species rather constant in size, length 1.06–1.34 mm, maximum width 0.94–1.4 mm. Anterior head margin evenly rounded or very slightly protruding. Thorax slightly or distinctly widening posteriorly. Coloration with multiple brown shades forming distinct pattern, mostly dark.

**Head capsule.** Ca. ⅓ – ⅖ as long as entire cephalothorax including lateral cephalic extension. Coloration of head dominantly pale or brown, forming specific color pattern. Clypeal region well delimited from labral area, arcuate, or very slightly protruding and forming clypeal lobe. Surface smooth or slightly wrinkled. Ca. 50–95 sensilla regularly dispersed on clypeal area. Border between clypeal area and frontal region clearly recognizable or indistinct. Frontal region smooth or slightly wrinkled. Segmental border between head and prothorax quite distinct on dorsal side, indicated by furrow, change in cuticular sculpture or coloration.

**Supra-antennal sensillary field.** Slightly wrinkled or reticulated, delimited by more or less distinct furrow on medial side (Fig. 45B).

**Figure 44.**
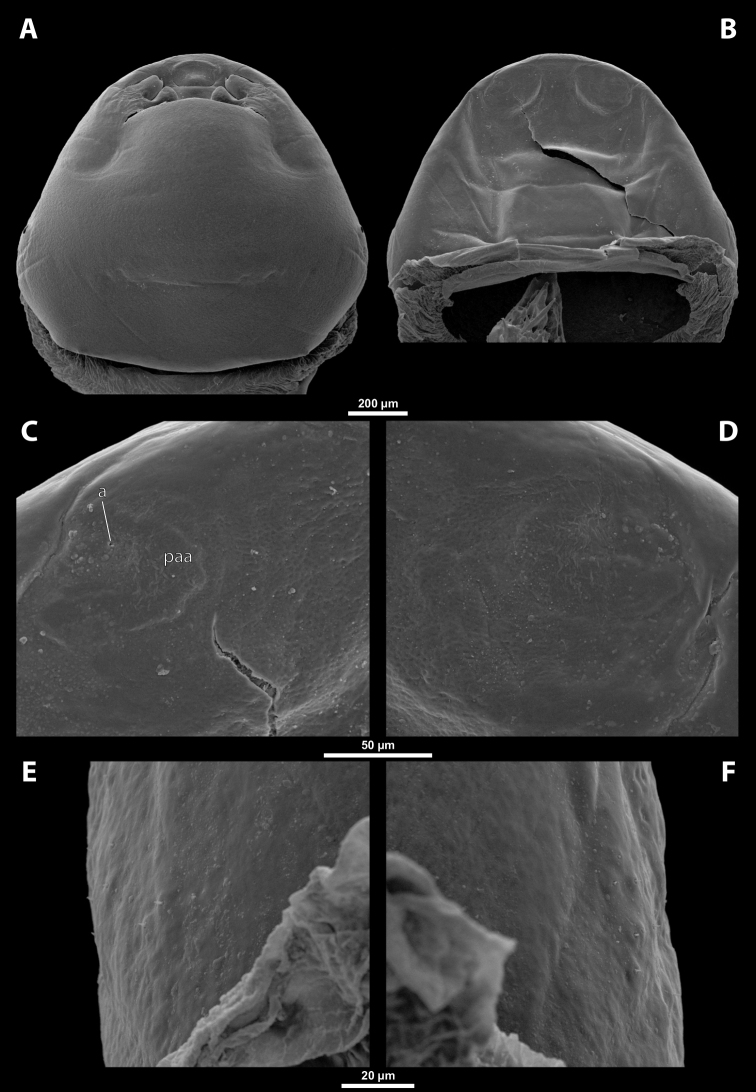
*Tuberoxenossphecidarum*, female, cephalothorax, SEM micrographs **A** ventral side **B** dorsal side **C** left vestigial antenna, dorsal side **D** right vestigial antenna, dorsal side **E** left lateral border of abdominal segment I below spiracle, dorsal side **F** right lateral border of abdominal segment I below spiracle, dorsal side. Abbreviations: a – vestigial antenna, paa – periantennal area.

**Antenna.** Preserved as poorly defined area, in some cases indistinct (Fig. 44C). With cavities, several small, rounded plates, or sensilla, the latter combined in some cases. Periantennal area smooth or slightly wrinkled (Fig. 44C).

**Figure 45.**
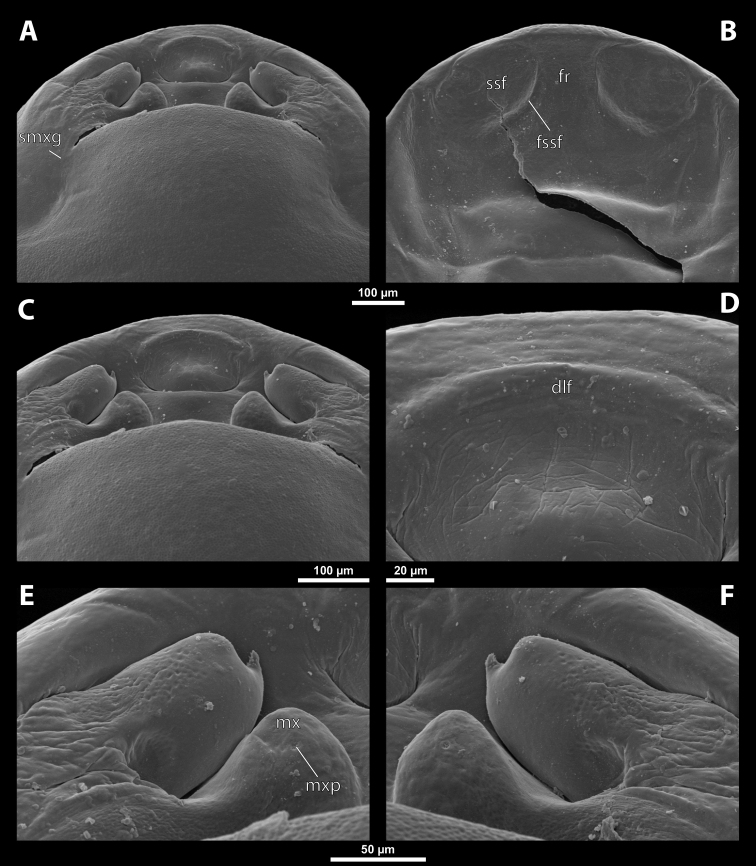
*Tuberoxenossphecidarum*, female, cephalothorax, SEM micrographs **A** anterior part of cephalothorax, ventral side **B** anterior part of cephalothorax, dorsal side **C** mouthparts, ventral side **D** detail of anterior border of cephalothorax, ventral side **E** right mandible and maxilla, ventral side **F** left mandible and maxilla, ventral side. Abbreviations: dlf – dorsal field of labral area, fr – frontal region, fssf – furrow of supra-antennal sensillary field, mx – vestige of maxilla, mxp – vestige of maxillary palp, smxg – submaxillary groove, ssf – supra-antennal sensillary field.

**Labrum.** Ventral field at least slightly wider than long, elliptical or semicircular. Dorsal field widely arcuate, ~ 5× wider than long in midline, distinctly raised (Fig. 45D). Dorsal field with ~ 17–28 pointed or blunt setae on its surface.

**Mandible.** Anteromedially directed at angle of 20–40°, enclosed in mandibular capsule. Mandibular bulge slightly or distinctly raised, with several sensilla. Cuticle smooth, slightly sculptured or reticulated. Longitudinal grooves on articular area present. Tooth narrow, pointed, more or less armed with spines.

**Maxilla.** Well developed and clearly separated from labial area, prominent and anteriorly directed. Protruding maxillary part usually slightly overlapping with proximal portion of mandible (Fig. 45E), but not projecting beyond mandible anteriorly. Cuticle smooth or very slightly wrinkled. Vestige of palp inconspicuous, preserved as small bulge with indistinct plates, located anteromedially on ventral side of maxilla (Fig. 45E). Maxillary base distinctly produced anterolaterally as submaxillary groove.

**Labium.** Labial area distinct between maxillae, delimited anteriorly by mouth opening and posteriorly by birth opening. Labial area wider than long in midline, flat or slightly convex. Cuticular surface smooth or slightly reticulated.

**Mouth opening.** Arcuate, nearly straight, or bi-arcuate, sclerotized marginally.

**Thorax and abdominal segment I.** Pro-mesothoracic and meso-metathoracic borders distinct, usually separated by mesal furrows, often combined with color stripes or spots on dorsal and ventral sides. Border between metathorax and abdomen usually indicated by change of cuticular sculpture and very indistinct ridge. Cuticle of thoracic segments on ventral side reticulate with scattered small and pigmented papillae. Dorsal side of thorax smooth or slightly reticulated. Prosternal extension undifferentiated, prosternal margin evenly arched but in some cases protruding and overlapping with maxillolabial area and posterior part of mandibles. Meso- and metathorax of standard transverse shape. Setae present on lateral region of abdominal segment I (Fig. 44 E, F).

**Spiracles.** Spiracles on posterior half or third of cephalothorax slightly elevated, with lateral or anterolateral orientation.

##### Diagnosis of male cephalotheca.

**Figure 46.**
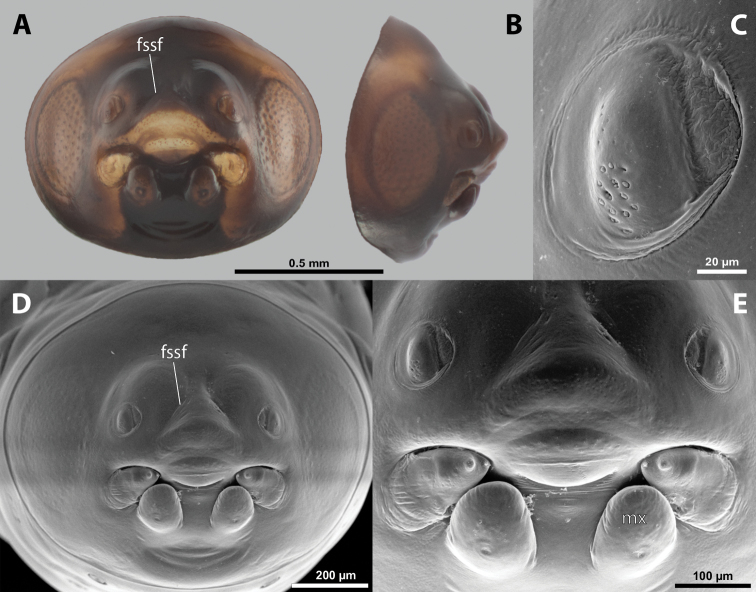
*Tuberoxenossphecidarum*, male, cephalotheca, photomicrographs, SEM micrographs **A** frontal view **B** lateral view **C** vestigial antenna **D** frontal view **E** mouthparts. Abbreviations: fssf – furrow of supra-antennal sensillary field, mx – vestige of maxilla.

Differing from other genera by the following combination of characters. Diameter of genae between maxillary base and compound eye ~ 1.5× larger than diameter of vestigial antenna. Occipital bulge absent and frontal impression indistinct or missing. Distinct paired furrows of supra-antennal sensillary field present (Fig. 46A, D). Cephalotheca always appearing rotund.

##### Description of male cephalotheca.

**Shape and coloration.** In frontal view rounded, almost circular (Fig. 46A), in lateral view pointed anteriorly. Coloration forming pattern of pale and dark shades.

**Cephalothecal capsule.** Compound eyes with darker individual ommatidia well visible on pale ocular background. Conspicuous clypeal lobe arcuate in frontal view, prominent in lateral view. Sensilla dispersed over entire clypeal area. Paired furrows of supra-antennal sensillary field distinctly presented but impression lacking on frontal region. Occipital bulge absent. Diameter of genae between maxillary base and compound eye small, ~ 1.5× larger than diameter of vestigial antenna.

**Supra-antennal sensillary field.** Kidney-shaped and bulging, medially delimited by distinct furrow (Fig. 46A, D).

**Antenna.** Large, with complete torulus. Periantennal area not clearly delimited from supra-antennal sensillary field. Small plates, cavities and sensilla present (Fig. 46C).

**Labrum.** Labral area distinct. Setae on dorsal field present.

**Mandible.** Anteromedially directed. Tooth pointed, not reaching area of mandibular bulge basally. Bulge with sensilla.

**Maxilla.** Distinct, prominent. Coloration completely dark or brighter around distinct vestige of maxillary palp.

**Labium and hypopharynx.** Labium distinct between and below maxillae, dark. Praementum and postmentum separated by furrow. Hypopharyngeal protuberance indistinct or absent.

**Mouth opening.** Well visible, not covered by ventral labral field, slightly arcuate.

##### Phylogenetic relationships.

Deeply nested within Xenidae (Benda et al. 2019, 2021), part of a clade of an Old Word origin, with *Pseudoxenos* Saunders as sister group.

##### Diversity and distribution.

A lineage of Afrotropical-Palearctic origin, comprising 5 currently valid species, restricted to these regions. It is an example of connectivity between both biogeographic regions (Benda et al. 2019).

##### Hosts.

*Ammophila* and *Podalonia* spp. (Sphecidae: Ammophilinae), rarely *Prionyx* spp. (Sphecidae: Sphecinae).

##### Etymology.

From the Latin substantive *tuber*, meaning a swelling. The name refers to conspicuous swellings on the host abdomen caused by protruded xenid specimens under tergites or sternites. Gender masculine.

##### Comments.

All described species of *Tuberoxenos* gen. nov. were previously placed in *Paraxenos* based on parasitising Sphecidae (Kinzelbach 1971b). Despite this concept, this group is morphologically well defined. We classify it as a separate genus, based on molecular phylogenies (Benda et al. 2019, 2021) and morphological characters newly reported in this paper.

### ﻿List of species

#### ﻿ Tuberoxenos altozambeziensis

Taxon classificationAnimaliaStrepsipteraXenidae

(Luna de Carvalho, 1959) comb. nov.

895338ED-D3E8-5748-938B-B3AAA5BF02D9

Pseudoxenos altozambeziensis Luna de Carvalho, 1959: 136.
Paraxenos altozambeziensis (Luna de Carvalho, 1959) (new combination by Kinzelbach 1971b).

##### Hosts.

*Ammophila* sp. (Luna de Carvalho 1959); *Ammophilarubripes* Spinola, 1839 (Benda et al. 2021).

##### Distribution.

Angola (Luna de Carvalho 1959); Tanzania (Benda et al. 2021).

#### ﻿ Tuberoxenos sinuatus

Taxon classificationAnimaliaStrepsipteraXenidae

(Pasteels, 1956) comb. nov.

1ACF0C04-FA5E-5EAE-B745-3BF6F3108747

Pseudoxenos sinuatus Pasteels, 1956: 115.
Paraxenos sinuatus (Pasteels, 1956) (new combination by Kinzelbach 1971b).

##### Hosts.

*Ammophilapunctaticeps* (Arnold, 1920); *Podaloniatydei* (Le Guillou, 1841) (as *Ammophilatydei* Le Guillou, 1841) (Pasteels 1956; Kinzelbach 1971b); *Ammophilaargyrocephala* Arnold, 1951 (Benda et al. 2021).

##### Distribution.

Democratic Republic of Congo (Pasteels 1956); Tanzania (Benda et al. 2021).

#### ﻿ Tuberoxenos sphecidarum

Taxon classificationAnimaliaStrepsipteraXenidae

(Siebold, 1839) comb. nov.

91CDC942-6D57-58BB-A836-47FA9EE58C10

Xenos sphecidarum Siebold, 1839: 72.
Eupathocera sphecidarum (Dufour, 1837) (new combination by Pierce, 1908, incorrectly assigned authorship).
Paraxenos sieboldii Saunders, 1872 (synonymized by Pierce, 1909).
Paraxenos sieboldii (Dufour, 1837) (new combination by Pierce 1919, incorrectly assigned authorship).
Pseudoxenos sphecidarum (Dufour, 1837) (new combination by Bohart 1937, incorrectly assigned authorship).
Paraxenos sphecidarum (Dufour, 1837) (new combination by Kinzelbach 1971b, incorrectly assigned authorship).

##### Hosts.

*Ammophilaapicalis* Guérin-Méneville, 1835 (as *Ammophilaapicalis* Brullé, 1839); *A.campestris* Latreille, 1809; *A.heydeni* Dahlbom, 1845 (as *Ammophilaheydeni* Dahlberg?); *A.holosericea* (Fabricius, 1793); *A.nasuta* Lepeletier, 1845; *A.pubescens* Curtis, 1836; *A.sabulosa* (Linnaeus, 1758); *Podaloniaaffinis* (Kirby, 1798) (as *Ammophilaaffinis* Kirby, 1798); *P.dispar* (Taschenberg, 1869) (as *Ammophiladispar* Taschenberg, 1869); *P.ebenina* (Spinola, 1839) (as *Ammophilaebenina* Spinola, 1839); *P.hirsuta* (Scopoli, 1763) (as *Ammophilahirsuta* Scopoli); *P.nigrohirta* (Kohl, 1888) (as *Ammophilanigrohirta* Kohl, 1888); *P.tydei* (Le Guillou, 1841) (as *Ammophilatydei* Le Guillou, 1841); *Eremocharesdives* (Brullé, 1833) (as *Ammophiladives* Brullé, 1833); *Prionyxkirbii* (Vander Linden, 1827) (as *Sphexalbisectus* Lep. & Serv., 1828); *P.viduatus* (Christ, 1791) (as *Sphexviduatus* Christ, 1791); *P.niveatus* (Dufour, 1854) (as *Sphexniveatus* Dufour, 1854) (Kinzelbach 1978); *Ammophiladupla* Kohl, 1901; *Podaloniachalybea* (Kohl, 1906); *Podaloniaflavida* (Kohl, 1901) (Benda et al. 2021).

##### Distribution.

Poland: Gdańsk (Siebold 1839); Palearctic (Kinzelbach 1978).

##### Note.

Benda et al. (2021) proposed at least four distinctive *T.sphecidarum* lineages possibly representing separate species. More comprehensive sampling and detailed study are necessary.

#### ﻿ Tuberoxenos teres

Taxon classificationAnimaliaStrepsipteraXenidae

(Pasteels, 1950) comb. nov.

5BD31B69-CE76-5390-AE44-84A23C5E28AE

Pseudoxenos teres Pasteels, 1950: 289.
Paraxenos teres (Pasteels, 1950) (new combination by Kinzelbach 1971b).

##### Hosts.

*Ammophilabeniniensis* (Palisot de Beauvois, 1806) (as *Sphexbeniniensis* Palisot de Beauvois, 1806); *Ammophilabeniniensistomentosa* (Arnold, 1920) (as *Sphexbeniniensistomentosus* Arnold, 1920) (Kinzelbach 1971); *Ammophilaferrugineipes* Lepeletier, 1845 (as *Sphexbonaespeiferrugineipes* Lepeletier, 1845) (Kinzelbach 1971b, Pasteels 1950).

##### Distribution.

Democratic Republic of Congo (Pasteels 1950).

#### ﻿ Tuberoxenos tibetanus

Taxon classificationAnimaliaStrepsipteraXenidae

(Yang, 1981) comb. nov.

E8395BE8-4EAF-522C-AD56-A6F18987C74E

Paraxenos tibetanus Yang, 1981: 572.

##### Hosts.

*Ammophila* sp.

##### Distribution.

China.

##### Note.

The article from Yang (1981) could not be found despite of great effort and the citation is not available.

#### ﻿ Deltoxenos gen. nov.

Taxon classificationAnimaliaStrepsipteraXenidae

6AE7326E-1961-5C31-B8C8-8BD8E4EE9085

http://zoobank.org/78A7DB5E-AA8B-4DCE-9F60-2001D2B218CB

##### Type species.

*Pseudoxenosbidentatus* Pasteels, 1950, here designated.

##### Diagnosis of female cephalothorax.

**Figure 47.**
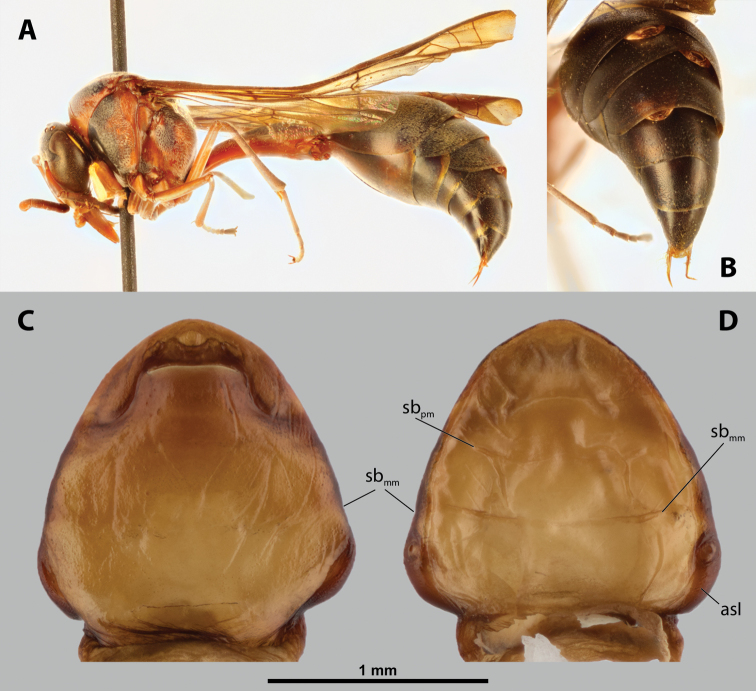
*Deltoxenosbidentatus*, host, female, cephalothorax, photomicrographs **A**Afreumenescf.aethiopicus stylopized by female of *D.bidentatus*, lateral view **B** detail of host abdomen with adult female **C** ventral side of cephalothorax **D** dorsal side of cephalothorax. Abbreviations: asI – abdominal segment I, sbmm – segmental border between mesothorax and metathorax, sbpm – segmental border between prothorax and mesothorax.

Maxilla not prominent, only slightly raised or nearly fused to labial area. Meso-metathoracic segmental border slightly or distinctly constricted laterally (Fig. 47C, D), especially in species with elongated cephalothorax. Pro-mesothoracic segmental border rarely constricted. Dorsal labral field slightly or distinctly arcuate, raised or flat, in the latter case narrower laterally than medially (Fig. 3A). Lateral parts of abdomen posterior to spiracles not pale (Figs 1B, 47D). Mandible not protruding from capsule. In contrast to *Paragioxenos*, head and prothorax ventrally delimited by birth opening medially and by suture laterally.

##### Description of female cephalothorax.

**Shape and coloration.** Very variable, ca. as long as wide, slightly wider than long, or distinctly longer than wide. Meso-metathoracic segmental border slightly or distinctly constricted laterally (Fig. 47C, D), especially in species with elongated cephalothorax. Pro-mesothoracic segmental border rarely constricted. Extremely variable in size, length 0.9–2.83 mm, maximum width 0.74–2.43 mm. Anterior head margin evenly rounded or protruding. Thorax slightly or distinctly widening posteriorly, sometimes nearly parallel-sided. Cephalothorax with conspicuous color pattern. Coloration comprising multiple brown and orange shades forming distinct pattern.

**Head capsule.** Ca. ¼ ~ ½ as long as entire cephalothorax including lateral cephalic extension. Coloration of head forming specific color pattern with pale and dark combined. Clypeal area well delimited from labral area, arcuate, or protruding and forming clypeal lobe. Surface smooth or slightly wrinkled. Sensilla (24 to 45 or more) regularly distributed on clypeal area or mainly concentrated on clypeal lobe. Border between clypeal region and frontal area not clearly distinguishable but border still recognizable. Cuticle of frontal region very variable, from distinctly wrinkled, slightly wrinkled to nearly smooth, or covered with distinct papillae. Border between head and prothorax well visible or faintly recognizable on dorsal side, often indicated by colored transverse stripe (Fig. 1B).

**Supra-antennal sensillary field.** Smooth, wrinkled or reticulated, with dispersed sensilla. Not delimited or indistinctly delimited by furrow on medial side, but border of field still distinctly visible (Figs 4A, 49B).

**Figure 48.**
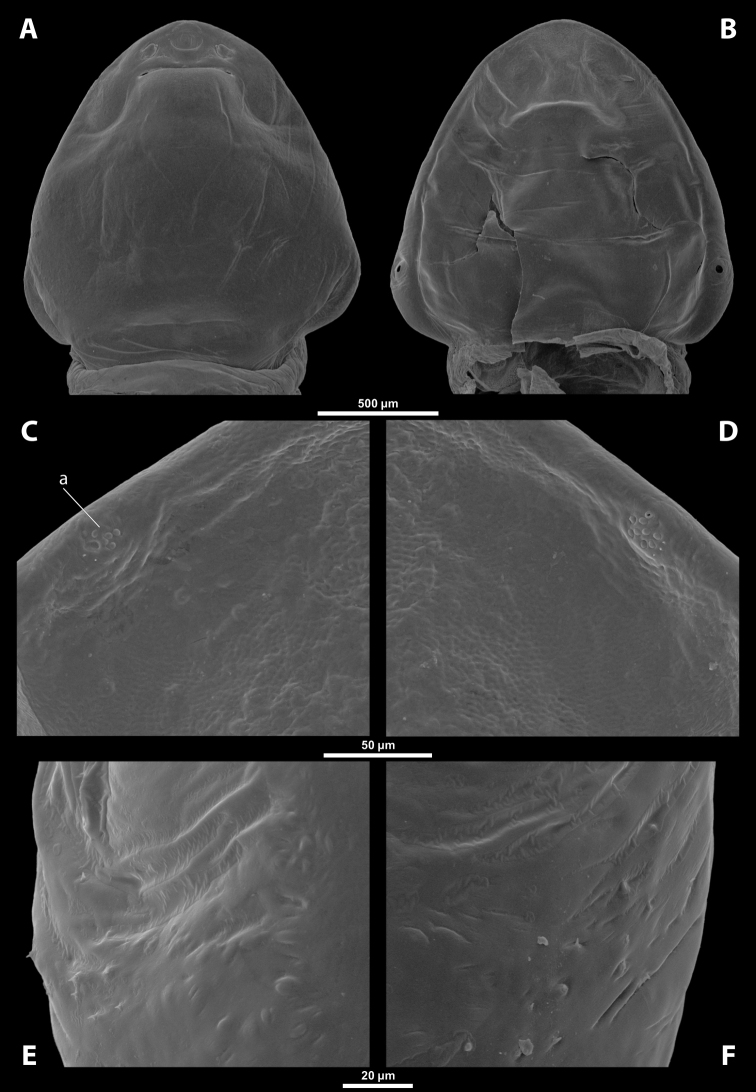
*Deltoxenosbidentatus*, female, cephalothorax, SEM micrographs **A** ventral side **B** dorsal side **C** left vestigial antenna, dorsal side **D** right vestigial antenna, dorsal side **E** left lateral border of abdominal segment I below spiracle, dorsal side **F** right lateral border of abdominal segment I below spiracle, dorsal side. Abbreviations: a – vestigial antenna.

**Antenna.** Preserved as poorly defined area, with several small, rounded plates, antennal sensilla, or cavity, often combined (Figs 4B, 48C). Periantennal area smooth or wrinkled.

**Labrum.** Ventral field wider than long, elliptic to nearly circular. Dorsal field slightly or distinctly arcuate, raised, or flat and laterally narrower than medially (Fig. 3A). Ca. 4–6× wider than long in midline. Dorsal field with ~ 10–25 setae or sensilla inserted in cavities.

**Figure 49.**
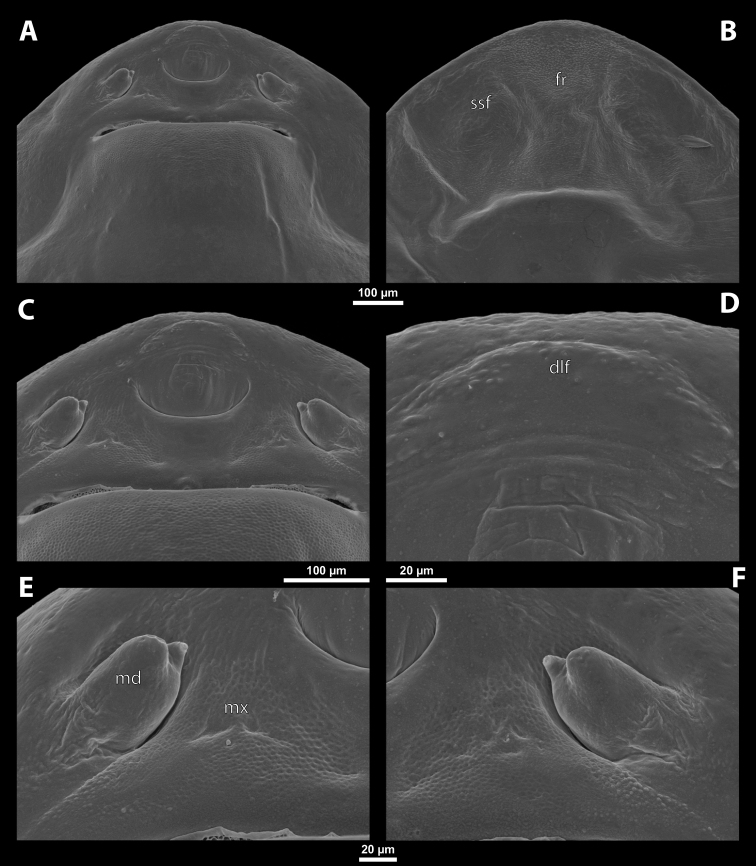
*Deltoxenosbidentatus*, female, cephalothorax, SEM micrographs **A** anterior part of cephalothorax, ventral side **B** anterior part of cephalothorax, dorsal side **C** mouthparts, ventral side **D** detail of anterior border of cephalothorax, ventral side **E** right mandible and maxilla, ventral side **F** left mandible and maxilla, ventral side. Abbreviations: dlf – dorsal labral field of labral area, fr – frontal region, md – mandible, mx – vestige of maxilla, ssf – supra-antennal sensillary field.

**Mandible.** Mandibles anteromedially directed at an angle of 25–65° and nested in mandibular capsule. Mandibular bulge distinctly raised, with several sensilla. Cuticle of mandible completely smooth to partially sculptured (Fig. 49E). Mandibular tooth narrow or slightly widened, pointed apically or ventrally, armed with spines.

**Maxilla.** Very variable in shape, distinctly reduced and almost fused with labial area, or slightly raised but not distinctly prominent (Figs 3B, 49E). Cuticle smooth or wrinkled. Apical maxillary region not or slightly projecting beyond mandibular apex. Basal portion firmly connected with labium and not overlapping with mandible, or in some cases elevated and overlapping with mandible very slightly. Vestige of palp inconspicuous, forming cavity or poorly defined area with indistinct plate. Usually located medially on ventral side of maxilla (Fig. 3B). Maxillary base more or less distinctly produced anterolaterally as submaxillary groove.

**Labium.** Labial area usually distinct between maxillae, delimited anteriorly by mouth opening and posteriorly by birth opening. Flat, longer than wide or wider than long. Cuticular surface smooth or reticulated.

**Mouth opening.** Widely arcuate to nearly straight or bisinuate, sclerotized along margin.

**Thorax and abdominal segment I.** Pro-mesothoracic and meso-metathoracic borders more or less distinct, usually separated by mesal furrows, combined with pigmented stripes or spots on dorsal and ventral side (Figs 1A, B, 47C, D). Border between metathorax and abdomen usually formed by ridge or indicated by change of cuticular sculpture (Fig. 1A). Cuticle of thoracic segments on ventral side reticulate with scattered inconspicuous or more distinct pigmented papillae. Dorsal side of thorax smooth or slightly reticulated. Prosternal extension undifferentiated, evenly arched. Meso- and metathorax usually transverse or elongated in some cases. Lateral parts of abdomen posterior to spiracles dark (Figs 1B, 47D). Setae and cuticular spines present on lateral region of abdominal segment I (Fig. 48E, F).

**Spiracles.** Located on posterior third of cephalothorax, slightly elevated with anterodorsal and anterolateral orientation.

##### Diagnosis of male cephalotheca.

**Figure 50.**
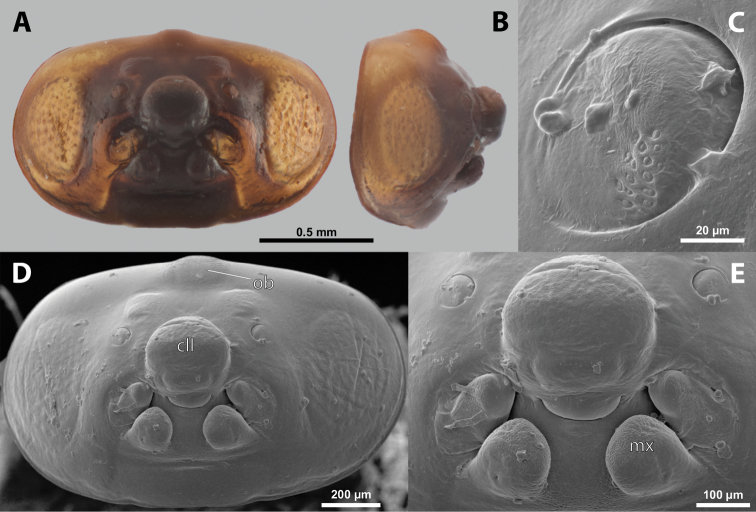
*Deltoxenosrueppelli*, male, cephalotheca, photomicrographs, SEM micrographs **A** frontal view **B** lateral view **C** vestigial antenna **D** frontal view **E** mouthparts. Abbreviations: cll – clypeal lobe, mx – vestige of maxilla, ob – occipital bulge.

Differing from other genera by the following combination of characters. Diameter of genae between maxillary base and compound eye at least 2× as large as diameter of vestigial antenna. Distinct paired furrow of supra-antennal sensillary field absent. Cephalotheca always elliptic (Figs 5A, 50A). Frontal fissure hardly distinct of nearly absent (Figs 6A, 50D). Maxilla not distinctly elongated, at most 1.5× longer than basally wide (Fig. 50E). Occipital bulge well developed (Figs 6A, 50D). Coloration forming pattern of pale and dark shades (Figs 5A, 50A).

##### Description of male cephalotheca.

**Shape and coloration.** In frontal view rounded laterally, elliptic, in lateral view pointed anteriorly. Coloration forming pattern of pale and dark shades.

**Cephalothecal capsule.** Compound eyes with darker individual ommatidia well visible on pale ocular background. Clypeal lobe straight or slightly arcuate in frontal view, prominent in lateral view, in some cases bulging (Figs 6B, 50D). Sensilla mainly concentrated on clypeal lobe. Frontal impression more or less distinct (Figs 5A, 6A). Occipital bulge distinct (Figs 5A, 50D). Diameter of genae between maxillary base and compound eye smaller, > 2× larger than diameter of vestigial antenna.

**Supra-antennal sensillary field.** Kidney-shaped and bulging, medially delimited by more or less distinct frontal impression, lacking furrows.

**Antenna.** Of standard shape, with recognizable complete torulus. Periantennal area not clearly delimited from supra-antennal sensillary field. Small plates, cavities or sensilla present.

**Labrum.** Labral area distinct, with setae on dorsal field.

**Mandible.** Anteromedially directed. Mandibular bulge with sensilla, separated from pointed tooth.

**Maxilla.** Distinct, prominent, completely dark. Vestige of palp distinct.

**Labium and hypopharynx.** Labium distinct between and below maxillae, dark. Praementum and postmentum separated by furrow. Hypopharyngeal protuberance present or not.

**Mouth opening.** Well visible, not covered by ventral labral field, distinctly arcuate.

##### Phylogenetic relationships.

Deeply nested within Xenidae, with *Xenos* as sister group (Benda et al. 2019; Straka and Benda unpubl. results).

##### Diversity and distribution.

A lineage of Afrotropical origin with later expansion to the Palearctic and Indomalayan regions (Benda et al. 2019). Present distribution of 7 species comprising the Old World and Australasian region.

##### Hosts.

Various genera of Eumenini and Odynerini (Vespidae: Eumeninae).

##### Etymology.

Name derived from the generic name *Delta* Saussure, one of the most common host genera. Gender masculine.

##### Comments.

All described species of *Deltoxenos* gen. nov. were previously placed in *Pseudoxenos* based on parasitism in solitary wasps (Kinzelbach 1971b). Despite this concept, this group is morphologically well defined. Although this group was not recognized in Kinzelbach’s concept, we classify it as a separate genus based on molecular phylogenies (Benda et al. 2019, 2021) and morphological characters newly reported here.

### ﻿List of species

#### ﻿ Deltoxenos bequaerti

Taxon classificationAnimaliaStrepsipteraXenidae

(Luna de Carvalho, 1956) comb. nov.

4440A5DA-EFA4-5F2F-8331-E12B1C2D7AE8

Pseudoxenos bequaerti Luna de Carvalho, 1956: 40.

##### Host.

*Antepiponatropicalis* (Saussure, 1853) (as *Rygchiumtropicale* Saussure, 1853) (Luna de Carvalho 1956).

##### Distribution.

Angola: Dundo (Luna de Carvalho 1956).

#### ﻿ Deltoxenos bidentatus

Taxon classificationAnimaliaStrepsipteraXenidae

(Pasteels, 1950) comb. nov.

17E399C4-780F-59FF-85E3-2C9C3B905DB3

Pseudoxenos bidentatus Pasteels, 1950: 288.

##### Hosts.

*Afreumenesmelanosoma* (Saussure, 1852) (as *Eumenesmelanosomadecipiens* Kirby, 1896); *Deltatropicale* (Saussure, 1852) (Benda et al. 2021); Afreumenescf.aethiopicus (Saussure, 1852) (this study).

##### Distribution.

Democratic Republic of Congo; Liberia (Pasteels 1950; Luna de Carvalho 1978a); Central African Republic (Benda et al. 2021); Malawi (this study).

#### ﻿ Deltoxenos hirokoae

Taxon classificationAnimaliaStrepsipteraXenidae

(Kifune & Yamane, 1992) comb. nov.

A214E14E-FFBF-5941-B40A-C76027D37238

Pseudoxenos hirokoae Kifune & Yamane, 1992: 343.

##### Host.

*Stenodynerusrufomaculatus* Sk. Yamane & Gusenleitner, 1982.

##### Distribution.

Japan: Amami Oshima (Kifune and Yamane 1992).

##### Note.

No DNA sequences from Xenidae parasitizing *Stenodynerus* Saussure in East Asia have been available. Strepsipterans parasitizing *Stenodynerus* in Japan are preliminarily included in *Deltoxenos* gen. nov. here based on their morphology, which, however, should be supported by future molecular phylogenetic analyses.

#### ﻿ Deltoxenos iwatai

Taxon classificationAnimaliaStrepsipteraXenidae

(Esaki, 1931) comb. nov.

1CD2B4BB-B695-5900-878D-3A1251EDF9C7

Pseudoxenos iwatai Esaki, 1931: 63.

##### Host.

*Oreumenesdecoratus* (Smith, 1852) (as *Eumenesjaponica* Saussure, 1858) (Esaki 1931).

##### Distribution.

Japan (Esaki 1931).

#### ﻿ Deltoxenos lusitanicus

Taxon classificationAnimaliaStrepsipteraXenidae

(Luna de Carvalho, 1960) comb. nov.

1F4D5C75-CBF0-5CEC-B083-AD99CC6E20FB

Pseudoxenos lusitanicus Luna de Carvalho, 1960: 2.

##### Host.

*Ancistrocerusrenimacula* Lepeletier, 1841 (as *Ancistrocerusrecinula* Lepeletier, 1841) (Kinzelbach 1971).

##### Distribution.

Portugal (Luna de Carvalho 1960); Palearctic (Benda et al. 2021).

##### Note.

This species corresponds to a lineage widely distributed from Portugal to Mongolia (Benda et al. 2021). Although its phylogenetic position is still unclear, it is provisionally included into *Deltoxenos* gen. nov. here based on morphology.

#### ﻿ Deltoxenos minor

Taxon classificationAnimaliaStrepsipteraXenidae

(Kifune & Maeta, 1978) comb. nov.

2D9BBE4E-5AE0-5264-9E5B-AB1454D52CBB

Pseudoxenos minor Kifune & Maeta, 1978: 416.

##### Host.

*Stenodynerusfrauenfeldi* (Saussure, 1867).

##### Distribution.

Japan: Nagano Pref., Fukuoka Pref. (Kifune and Maeta 1978).

##### Note.

See the comment under *D.hirokoae*.

#### ﻿ Deltoxenos rueppelli

Taxon classificationAnimaliaStrepsipteraXenidae

(Kinzelbach, 1971a) comb. nov.

8C710230-0C05-5AEF-AE61-D842E9F8E921

Pseudoxenos rueppelli Kinzelbach 1971a: 272.

##### Hosts.

*Deltafenestrale* (Saussure, 1852) (as *Deltafenestralis* Saussure, 1852), *Deltaemarginatum* (Linnaeus, 1758) (as *Eumenestinctor* Christ, 1791 = *E.maxillosus* (De Geer, 1783)); *Deltacaffrum* (Linnaeus, 1767) (Benda et al. 2021).

##### Distribution.

Ethiopia (Kinzelbach 1971a); Tanzania (Benda et al. 2021); Kenya; Namibia; Yemen (this study).

#### ﻿ Xenos

Taxon classificationAnimaliaStrepsipteraXenidae

Rossi, 1794

8EB21A8E-4CA2-5DBF-986F-F4296103FD72

Xenos Rossi, 1794: 114. Type species: Xenosvesparum (Rossi, 1793), by monotypy.
Acroschismus Pierce, 1908: 79 (synonymized by Bohart 1941). Type species: Acroschismushubbardi Pierce, 1908.
Schistosiphon Pierce, 1908: 80 (synonymized by Bohart 1941). Type species: Xenospeckii Kirby, 1813.
Vespaexenos Pierce, 1909: 133 (synonymized by Bohart 1941). Type species: Vespaexenoscrabronis Pierce, 1909.
Belonogastechthrus Pierce, 1911: 498 (synonymized by Bohart 1941). Type species: BelonogastechthruszavattariiPierce 1911.
Clypoxenos Brèthes, 1923: 46 (synonymized by Bohart 1941). Type species: Clypoxenosamericanus Brèthes, 1923.

##### Diagnosis of female cephalothorax.

Differing from other genera by the combination of following characters. Clypeal sensilla distinct, position on clypeal lobe extended onto ventral side, often present near clypeo-labral border (Fig. 53D). Maxilla variable in shape, almost fused with labial area, or raised from it, but not distinctly prominent anteriorly (Fig. 53E, F). Reduced forms of maxilla often indistinctly separated from labial area. Cuticle of maxilla in some cases strongly sclerotized like in *Brasixenos*, but border between clypeus and labrum always distinct (Fig. 53D). Prosternal extension not differentiated. Mandible not protruding from capsule. In contrast to *Paragioxenos*, head and prothorax ventrally delimited by birth opening medially and by suture laterally.

##### Description of female cephalothorax.

**Figure 51.**
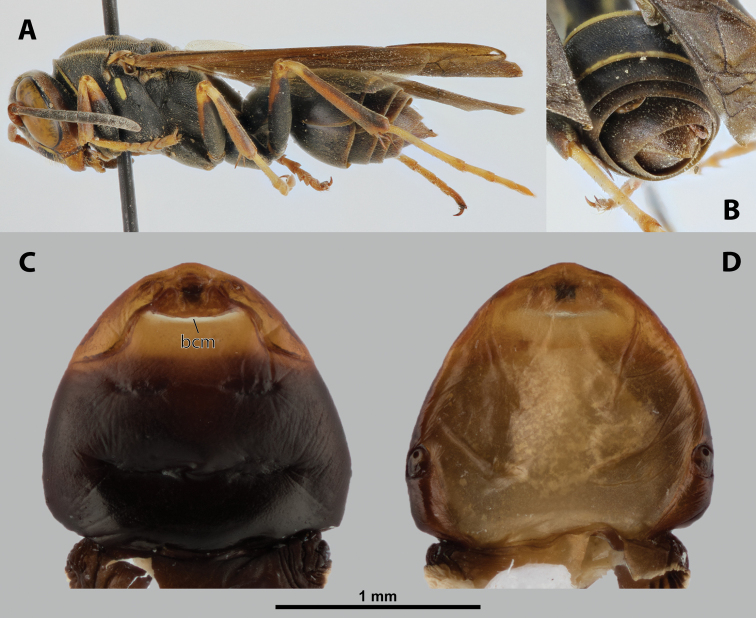
*Xenospeckii*, host, female, cephalothorax, photomicrographs **A***Polistesfuscatus* stylopized by two females of *X.peckii*, lateral view **B** detail of host abdomen with two adult females inside **C** ventral side of cephalothorax **D** dorsal side of cephalothorax. Abbreviations: bcm – brood canal membrane.

**Shape and coloration.** Extremely variable, ca. as long as wide, slightly wider than long, or distinctly longer than wide. Meso-metathoracic segmental border in some cases distinctly constricted laterally. Extremely variable in size, length 0.8–2.7 mm, maximum width 0.84–2.43 mm. Anterior head margin evenly rounded, protruding, or strongly protruding. Thorax slightly or distinctly widening posteriorly. Cephalothorax uniformly pale or colorful. Coloration with multiple brown (nearly black) and orange shades forming distinct pattern, often with pale anterior part and dark posterior area (Fig. 51C).

**Figure 52.**
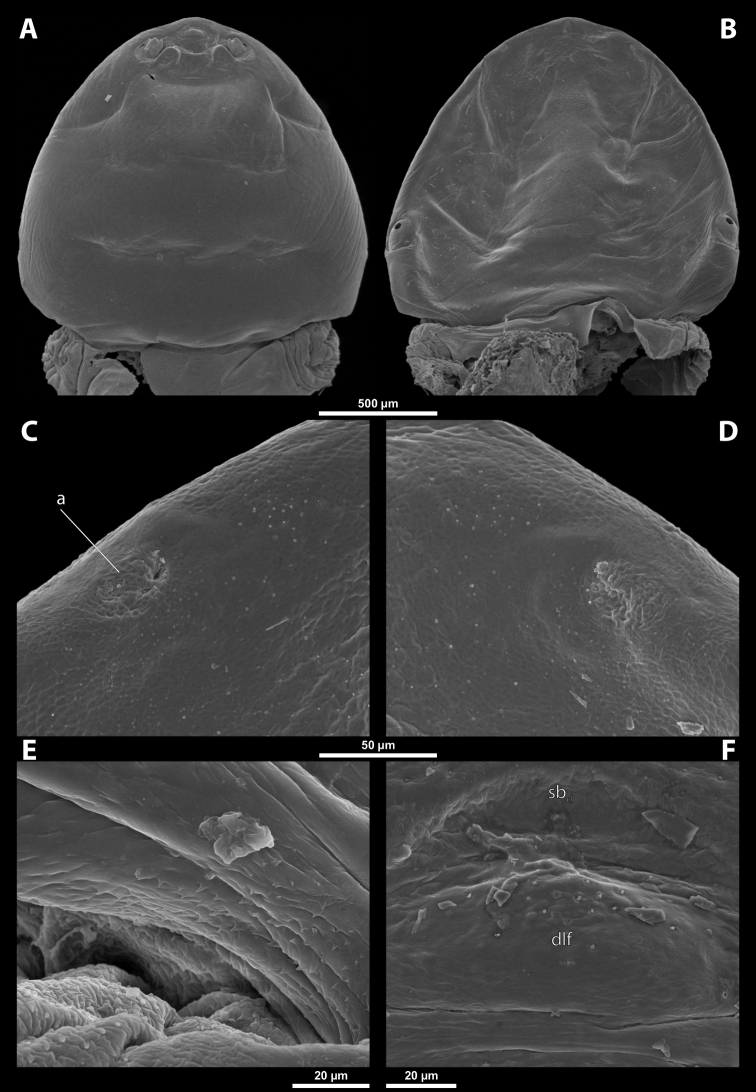
*Xenospeckii*, female, cephalothorax, SEM micrographs **A** ventral side **B** dorsal side **C** left vestigial antenna, dorsal side **D** right vestigial antenna, dorsal side **E** left lateral border of abdominal segment I below spiracle, dorsal side **F** detail of labral area, dorsal side. Abbreviations: a – vestigial antenna, dlf – dorsal labral field of labral area, sbcl – segmental border between clypeus and labrum.

**Figure 53.**
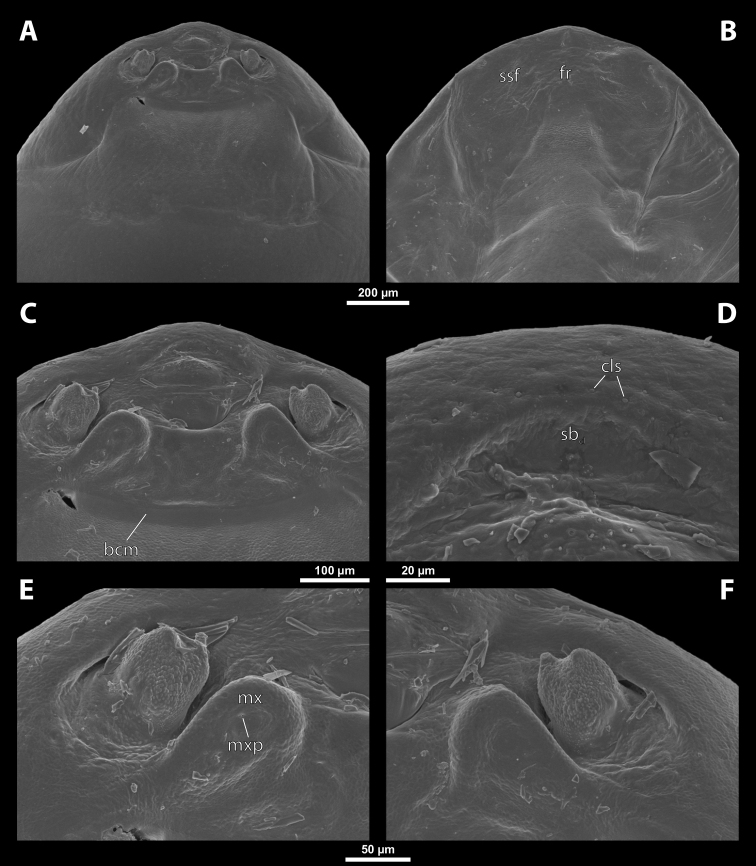
*Xenospeckii*, female, cephalothorax, SEM micrographs **A** anterior part of cephalothorax, ventral side **B** anterior part of cephalothorax, dorsal side **C** mouthparts, ventral side **D** detail of anterior border of cephalothorax, ventral side **E** right mandible and maxilla, ventral side **F** left mandible and maxilla, ventral side. Abbreviations: bcm – brood canal membrane, cls – clypeal sensillum, fr – frontal region, mx – vestige of maxilla, mxp – vestige of maxillary palp, sbcl – segmental border between clypeus and labrum, ssf – supra-antennal sensillary field.

**Head capsule.** Ca. ⅓ ~ ½ as long as entire cephalothorax including lateral cephalic extension. Coloration forming specific pattern with pale and dark combined. Clypeal region well delimited from labral area, border between clypeus and labrum often distinct (Figs 52F, 53D). Clypeal area variable in shape, apical margin arcuate, nearly flat, or protruding, forming distinct clypeal lobe. Cuticle smooth or slightly wrinkled. Numerous distinct sensilla present on clypeal surface, between 20 and 60 (or more), mainly concentrated anteriorly, rarely also scattered laterally, on clypeal lobe extending to ventral side, often near indistinct clypeo-labral border (Fig. 53D). Cuticle of frontal region slightly wrinkled. Segmental border between head and prothorax often indistinct to almost absent, at most indicated by change of color or transverse colored stripe.

**Supra-antennal sensillary field.** Slightly wrinkled with dispersed sensilla. Not delimited or indistinctly delimited by furrow medially, but border usually still recognizable (Fig. 53B).

**Antenna.** Preserved as poorly defined area, usually with several small, rounded plates, antennal sensilla, or cavity (Fig. 52C), in some cases combined, but antennal vestige in some cases only visible as strongly sculptured cuticle, without any plates or sensilla. Periantennal area wrinkled or reticulated.

**Labrum.** Ventral field variable, semicircular to nearly circular, elliptic, or subtriangular. Dorsal field slightly arcuate to straight, raised, or flat, ~ 4–5× wider than long in midline (Fig. 52F). Dorsal field laterally as long as medially, or laterally narrowed, with ~ 10–20 setae or sensilla inserted in cavities.

**Mandible.** Anteromedially directed at angle of 30–75° and enclosed in mandibular capsule, exceptionally slightly protruding. Mandibular bulge more or less distinctly raised, with several sensilla. Cuticle of mandible completely or partially sculptured. Tooth narrow or wider, pointed apically, more or less distinctly armed with spines.

**Maxilla.** Variable in shape, nearly fused with labial area and scarcely distinguishable from it, or raised but not distinctly prominent anteriorly (Fig. 53E, F). Cuticle smooth, wrinkled or reticulated, in some cases strongly sclerotized. Maxillary apex not projecting beyond mandible anteriorly but in some cases elevated maxillary base very slightly overlapping base of mandible. Vestige of palp inconspicuous, very poorly defined, often forming cavity or completely missing. If recognizable usually located medially or slightly apically on ventral side of maxilla (Fig. 53E). Maxillary base usually indistinctly produced anterolaterally as a submaxillary groove.

**Labium.** Labial area more or less recognizable between maxillae, delimited anteriorly by mouth opening and posteriorly by birth opening. Flat, slightly wider than long, as long as wide, or longer than wide. Cuticular surface smooth or reticulated.

**Mouth opening.** Widely arcuate to nearly straight or bisinuate, in some cases V-shaped, sclerotized along margin.

**Thorax and abdominal segment I.** Pro-mesothoracic and meso-metathoracic borders more or less distinct, usually indicated by mesal furrows, combined with pigmented stripes or spots on dorsal side. Border between metathorax and abdomen usually formed by ridge or indicated by change of cuticular sculpture. Cuticle of thoracic segments on ventral side reticulate with scattered small or larger pigmented papillae. Dorsal side of thorax smooth or slightly reticulated. Prosternal extension undifferentiated, evenly arched. Meso- and metathorax of standard transverse shape, in few cases constricted laterally. Setae and cuticular spines present on lateral region of abdominal segment I (Fig. 52E).

**Spiracles.** Spiracles on posterior third of cephalothorax slightly elevated, with anterodorsal and anterolateral orientation.

##### Diagnosis of male cephalotheca.

**Figure 54.**
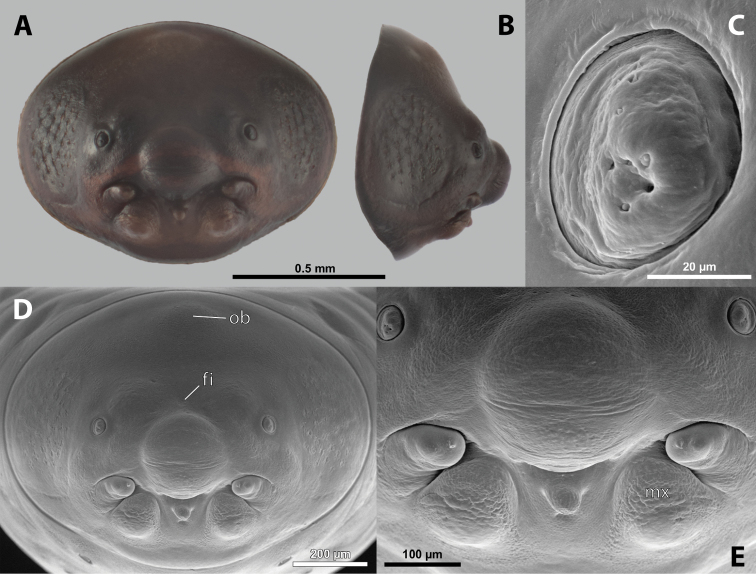
*Xenospeckii*, male, cephalotheca, photomicrographs, SEM micrographs **A** frontal view **B** lateral view **C** vestigial antenna **D** frontal view **E** mouthparts. Abbreviations: fi –frontal impression, mx – vestige of maxilla, ob – occipital bulge.

Differing from other genera by the following combination of characters. Diameter of genae between maxillary base and compound eye ~ 2–3× larger than diameter of vestigial antenna. Paired furrow of supra-antennal sensillary field slightly distinct or indistinct. Cephalotheca usually elliptic (Fig. 54A). Frontal fissure indistinct or almost absent (Fig. 54D). Maxilla not distinctly elongated, at most 1.5× longer than basally wide (Fig. 54E). Occipital bulge strongly reduced or missing (Fig. 54D). Cephalotheca mostly dark (Fig. 54A).

##### Description of male cephalotheca.

**Shape and coloration.** In frontal view rounded, elliptic, in lateral view slightly pointed anteriorly or rounded. Coloration with pattern of pale and dark shades but dark color dominant.

**Cephalothecal capsule.** Compound eyes completely dark or lighter, with dark individual cornea lenses visible. Clypeal lobe straight or slightly arcuate in frontal view, not or slightly prominent in lateral view. Sensilla mainly concentrated on clypeal lobe. Frontal impression inconspicuous or distinct (Fig. 54D). Occipital bulge indistinct (Fig. 54D) or absent. Diameter of genae between maxillary base and compound eye ~ 2–3× larger than diameter of vestigial antenna.

**Supra-antennal sensillary field.** Kidney-shaped and bulging, without furrows, delimited medially by more or less distinct frontal impression.

**Antenna.** Of standard shape, with small plates, cavities or sensilla, and complete torulus (Fig. 54C). Periantennal area not clearly delimited from supra-antennal sensillary field.

**Labrum.** Labral area distinct, with setae on dorsal field.

**Mandible.** Anteromedially directed. Mandibular bulge with sensilla, separated from pointed tooth.

**Maxilla.** Distinct, prominent, dark. Vestige of palp distinct.

**Labium and hypopharynx.** Dark labium distinctly visible between and below maxillae. Praementum and postmentum separated by indistinct transverse furrow. Hypopharyngeal protuberance present.

**Mouth opening.** Well visible, not covered by ventral labral field, slightly or distinctly arcuate.

##### Phylogenetic relationships.

Deeply nested within Xenidae, representing the largest radiation (Benda et al. 2021), sister to *Deltoxenos* gen. nov. (Benda et al. 2019; Straka and Benda unpubl. results)

##### Diversity and distribution.

The geographic origin is unclear, probably the New World or Afrotropical region (Benda et al. 2019). The present distribution of 33 described species comprising the Old and New World.

##### Hosts.

Several tribes of social Vespidae (Vespini, Polistini, Mischocyttarini, and Ropalidiini).

##### Comments.

The first species of Strepsiptera, *Xenosvesparum*, was superficially described by Rossi (1793), who assigned it to the genus *Ichneumon* in Hymenoptera. The genus *Xenos* was introduced later by Rossi (1794). Pierce (1908, 1909, 1911) described several genera (*Acroschismus*, *Belonogastrechthrus*, *Schistosiphon*, *Vespaexenos*) based on his hypothesis of host specialization. These were later synonymized with *Xenos* by Bohart (1941), and also the genus *Clypoxenos* described by Brèthes (1923). Kinzelbach (1971b) maintained this concept and extended it to *Brasixenos*, and considered representatives of *Xenos* as parasites of social wasps. Benda et al. (2019, 2021) revealed xenids parasitizing social Vespidae as a polyphyletic group. We classify *Xenos* as a valid genus based on the monophyly revealed by molecular phylogenies (Benda et al. 2019, 2021) and based on morphological characters newly reported here.

### ﻿List of species

#### ﻿ Xenos afer

Taxon classificationAnimaliaStrepsipteraXenidae

Pasteels, 1950

F084E123-E0E5-52C2-91F8-DFE1094102E8

Xenos afer Pasteels, 1950: 284.

##### Hosts.

*Polistesmarginalis* (Fabricius, 1775); *P.tristis* Meade-Waldo, 1911 (as *Polistessmithitristis* Meade-Waldo, 1911); *P.africanus* Palisot de Beuvois, 1818 (as *P.marginalis* v. *africanus* Palisot de Beuvois, 1818) (Pasteels 1950; Luna de Carvalho 1956).

##### Distribution.

Democratic Republic of Congo (Pasteels 1950); Angola (Luna de Carvalho 1956); Central African Republic; Ethiopia; Zanzibar (Benda et al. 2021).

#### ﻿ Xenos americanus

Taxon classificationAnimaliaStrepsipteraXenidae

(Brèthes, 1923)

8CD0DC36-FE19-57E0-AB21-62004DFFC0C9

Clypoxenos americanus Brèthes, 1923: 46.
Xenos americanus (Brèthes, 1923) (new combination by Bohart 1941).

##### Host.

*Mischocyttarusflavicans* (Fabricius, 1804) (as *Clypeopolybiaduckei* Brèthes, 1923) (Brèthes 1923).

##### Distribution.

Bolivia (Brèthes 1923).

#### ﻿ Xenos argentinus

Taxon classificationAnimaliaStrepsipteraXenidae

Brèthes, 1923

0BC3C763-439A-5552-BA11-FD84DD48F1F0

Xenos argentinus Brèthes, 1923: 43.

##### Hosts.

*Polistescavapyta* Saussure, 1853 (Brèthes 1923); *Polistesbuyssoni* Brethes, 1903 (this study).

##### Distribution.

Argentina: San Luis (Brèthes 1923), Cachi (this study).

#### ﻿ Xenos boharti

Taxon classificationAnimaliaStrepsipteraXenidae

Hofmann, 1965

B2F04548-70DE-5662-A564-6F275C564EFB

Xenos boharti Hofmann, 1965: 35.

##### Host.

*Polistesperuvianus* Bequard, 1934 (Hofmann 1965).

##### Distribution.

Chile: Tarapacá (Hofmann 1965).

#### ﻿ Xenos bohlsi

Taxon classificationAnimaliaStrepsipteraXenidae

Hoffmann, 1914

F5D3F2B7-DA11-5F7C-AB62-71EDBA6FE1B0

Xenos bohlsi Hoffmann, 1914: 100.

##### Host.

*Polistescanadensiscanadensis* (Linnaeus, 1758) (Hoffmann 1914).

##### Distribution.

Argentina; Brazil; Paraguay (Hoffmann 1914; Oliveira and Kogan 1962; Kinzelbach 1971b).

#### ﻿ Xenos bonairensis

Taxon classificationAnimaliaStrepsipteraXenidae

Brèthes, 1923

B24F3D32-27A2-5B13-B27B-99A57AF391A6

Xenos bonairensis Brèthes, 1923: 44.

##### Host.

*Polistesversicolor* (Olivier, 1792) (Brèthes 1923).

##### Distribution.

Argentina: Buenos Aires (Brèthes 1923); Brazil (Luna de Carvalho 1978b).

#### ﻿ Xenos circularis

Taxon classificationAnimaliaStrepsipteraXenidae

Kifune & Maeta, 1985

8B5B6AAE-9B4E-5EE5-80B9-07ECFFB068F1

Xenos circularis Kifune & Maeta, 1985: 430.

##### Host.

*Polistesrothneyigressitti* Vecht, 1968 (Kifune and Maeta 1985).

##### Distribution.

Taiwan (Kifune and Maeta 1985).

#### ﻿ Xenos colombiensis

Taxon classificationAnimaliaStrepsipteraXenidae

Cook, Mayorga-Ch & Sarmiento, 2020

94999E5E-A650-5F71-B255-F50AD2043ED1

Xenos colombiensis Cook, Mayorga-Ch & Sarmiento, 2020: 332.

##### Host.

*Polistesmyersi* Bequaert, 1934 (Cook et al. 2020).

##### Distribution.

Colombia (Cook et al. 2020).

#### ﻿ Xenos dianshuiwengi

Taxon classificationAnimaliaStrepsipteraXenidae

Yang, 1999

C1A8AB15-F7DD-5A2E-A109-D2F31EEB573F

Xenos dianshuiwengi Yang, 1999: 186.

##### Host.

*Vespa* sp. (Yang 1999).

##### Distribution.

China: Fujian (Yang 1999).

#### ﻿ Xenos formosanus

Taxon classificationAnimaliaStrepsipteraXenidae

Kifune & Maeta, 1985

3B2FB58B-E93B-5964-8A3A-EFC91A6F0CC6

Xenos formosanus Kifune & Maeta, 1985: 426.

##### Host.

*Vespavelutinaflavitarsus* Sonan, 1939 (Kifune and Maeta 1985).

##### Distribution.

Taiwan (Kifune and Maeta 1985).

#### ﻿ Xenos hamiltoni

Taxon classificationAnimaliaStrepsipteraXenidae

Kathirithamby & Hughes, 2006

600B218E-932C-5965-B425-1ABF785AD2DD

Xenos hamiltoni Kathirithamby & Hughes, 2006: 37.

##### Host.

*Polistescarnifex* (Fabricius, 1775) (Kathirithamby and Hughes 2006).

##### Distribution.

Mexico: Veracruz (Kathirithamby and Hughes 2006).

#### ﻿ Xenos hebraei

Taxon classificationAnimaliaStrepsipteraXenidae

Kinzelbach, 1978

09964A08-B200-57D0-98B8-9D02E654AE40

Xenos hebraei Kinzelbach, 1978: 69.

##### Hosts.

*Polistesolivaceus* (De Geer, 1773) (as *Polisteshebraeus* Fabricius, 1787) (Kinzelbach 1978); *Polisteswattii* Cameron, 1900 (this study).

##### Distribution.

Iraq; India (Kinzelbach 1978); Oman (this study).

#### ﻿ Xenos hospitus

Taxon classificationAnimaliaStrepsipteraXenidae

Oliveira & Kogan, 1962

1674B0E0-F197-577E-9A03-518E2FFE63B4

Xenos hospitus Oliveira & Kogan, 1962: 7.

##### Host.

*Polistesversicolor* (Olivier, 1791) (as *Polistesversicolorvulgaris* Bequaert, 1934) (Oliveira and Kogan 1962).

##### Distribution.

Brazil: Santa Catarina (Oliveira and Kogan 1962); Ecuador (this study).

#### ﻿ Xenos hunteri

Taxon classificationAnimaliaStrepsipteraXenidae

(Pierce, 1909)

658482C4-32AA-5105-BF95-5A0CD499559F

Acroschismus hunteri Pierce, 1909: 130.
Xenos hunteri (Pierce, 1909) (new combination by Bohart 1941).

##### Host.

*Polistes* sp., near *P.minor* Palisot de Beauvois, 1818 (Pierce 1909).

##### Distribution.

USA: Texas (Pierce 1909).

#### ﻿ Xenos indespectus

Taxon classificationAnimaliaStrepsipteraXenidae

Oliveira & Kogan, 1962

BB4342FB-9D13-51D2-8BD4-A6FD5171EEAA

Xenos indespectus Oliveira & Kogan, 1962: 10.

##### Host.

*Polistes* sp. (Oliveira and Kogan 1962).

##### Distribution.

Brazil: São Paulo (Oliveira and Kogan 1962).

#### ﻿ Xenos iviei

Taxon classificationAnimaliaStrepsipteraXenidae

Kifune, 1983

DE03FE6C-C7EF-5277-B039-3FCAF6B72912

Xenos iviei Kifune, 1983: 330.

##### Host.

*Polistescrinitus* (Felton, 1764) (Kifune 1983).

##### Distribution.

Virgin Islands (Kifune 1983).

#### ﻿ Xenos kifunei

Taxon classificationAnimaliaStrepsipteraXenidae

Cook & Mathison, 1997

3174D66F-6C3D-56CC-98E7-4AAD4D02125A

Xenos kifunei Cook & Mathison, 1997: 246.

##### Host.

*Polistescomanchusnavajoe* Cresson, 1868 (Cook and Mathison 1997).

##### Distribution.

USA: Arizona (Cook and Mathison 1997; Garza and Cook 2021).

#### ﻿ Xenos moutoni

Taxon classificationAnimaliaStrepsipteraXenidae

Buysson, 1903

400BCD9C-1D37-58C2-8D14-0A1FBF80ECFC

Xenos moutoni Buysson, 1903: 175.
Vespaexenos moutoni (Buysson, 1903) (new combination by Pierce 1909).
Vespaexenos crabronis Pierce, 1909 (synonymized by Bohart 1941).
Vespaexenos buyssoni Pierce, 1909 (synonymized by Bohart 1941).
Vespaexenos matsumarai Szekessy, 1965 (synonymized by Kinzelbach 1971b).

##### Hosts.

*Vespaanalisnigrans* Buysson, 1903 (as *Vespanigrans* Buysson, 1903); *Vespacrabro* Linnaeus, 1758; *Vespaducalis* Smith, 1852; *Vespadybowskii* André, 1884; *Vespamandarinia* Smith, 1852; *Vespamandarinamagnifica* Smith, 1852 (as *Vespamagnifica* Smith, 1852); *Vespasimillima* Smith, 1868 (Buysson 1903; Nakase and Kato 2013).

##### Distribution.

China: Anhui, Yunnan; Taiwan; Japan; Laos (Buysson 1903; Nakase and Kato 2013).

#### ﻿ Xenos niger

Taxon classificationAnimaliaStrepsipteraXenidae

Pasteels, 1950

DAA50C38-DC6D-5A24-AA9A-84ACBDA0D503

Xenos niger Pasteels, 1950: 287.

##### Host.

*Polistestenellus* Buysson, 1905 (Pasteels 1950).

##### Distribution.

Democratic Republic of Congo (Pasteels 1950).

#### ﻿ Xenos nigrescens

Taxon classificationAnimaliaStrepsipteraXenidae

Brues, 1903

B13F1FCE-EB3E-5579-BDE3-358903C18F97

Xenos nigrescens Brues, 1903: 247.

##### Host.

*Polistescarolina* (Linneaus, 1767) (as *Polistesrubiginosus* Lepeletier, 1836) (Brues 1903; Cook 2019).

##### Distribution.

USA: Texas (Brues 1903), Georgia (Garza and Cook 2021).

##### Notes.

*Polistescarolina* (Linneaus, 1767) was listed as a host by Cook (2019), because it was a former synonym of *Polistesrubiginosus* Lepeletier, 1836, which does not occur in the USA. Kinzelbach (1971b) incorrectly stated Argentina as a location.

#### ﻿ Xenos oxyodontes

Taxon classificationAnimaliaStrepsipteraXenidae

Nakase & Kato, 2013

E8A00027-ADCC-5C71-A12C-8AA4E59E0232

Xenos oxyodontes Nakase & Kato, 2013: 333.

##### Hosts.

*Vespaanalis* Fabricius, 1775, *Vespasimillima* Smith, 1868 (Nakase and Kato 2013).

##### Distribution.

Japan; South Korea (Nakase and Kato 2013).

#### ﻿ Xenos pallidus

Taxon classificationAnimaliaStrepsipteraXenidae

Brues, 1903

04DC15C2-2154-5945-A0C0-DF16EF3789E0

Xenos pallidus Brues, 1903: 246.
Acroschismus hubbardi Pierce, 1908 (synonymized by Bohart 1941).
Acroschismus pallidus texensis Pierce, 1909 (synonymized by Bohart 1941).

##### Hosts.

*Polistesannularis* (Linnaeus, 1763); *Polistescrinitus* (Felton, 1764) (as Polistes (americanus) crinitus (Felton, 1764)); *Polistescarnifex* (Fabricius, 1775), *Polistesbellicosus* Cresson, 1872 (Brues 1903; Cook 2019, misspelt as *P.vellicosus*).

##### Distribution.

USA: Texas, Florida; Mexico (Brues 1903; Dunkle 1979).

#### ﻿ Xenos peckii

Taxon classificationAnimaliaStrepsipteraXenidae

Kirby, 1813

2280A9E1-D899-55EC-B6FA-8027765242CA

Xenos peckii Kirby, 1813: 116.
Xenos wheeleri Pierce, 1908 (synonymized by Bohart 1941).
Acroschismus bruesi Pierce, 1909 (synonymized by Bohart 1941).
Acroschismus pecosensis Pierce, 1909 (synonymized by Bohart 1941).
Acroschismus bowditchi Pierce, 1909 (synonymized by Bohart 1941).
Acroschismus texani Pierce, 1909 (synonymized by Bohart 1941).
Acroschismus maximus Pierce, 1909 (synonymized by Bohart 1941).
Xenos auriferi Pierce, 1911 (synonymized by Bohart 1941).
Xenos californicus Pierce, 1919 (synonymized by Bohart 1941).
Xenos pecki (incorrect subsequent spelling): Kinzelbach (1971b).

##### Hosts.

*Polistesapachus* Saussure, 1857 (as *Polistestexanus* Cresson, 1872); *Polistesaurifer* Saussure, 1853; *Polistescarolina* (Linnaeus, 1767) (as *Polistesrubiginosus* Lepeletier, 1836); *Polistesfuscatus* (Fabricius, 1793); *Polistesmetricus* Say, 1831 (Kirby 1813; Pierce 1908, 1909).

##### Distribution.

USA: Massachusetts (Kirby 1813; Pierce 1909), Connecticut, Michigan, Ohio, Texas, California (Kirby 1813; Pierce 1908, 1909, 1919), New Jersey, New York, Colorado, Wyoming (Garza and Cook 2021).

#### ﻿ Xenos peruensis

Taxon classificationAnimaliaStrepsipteraXenidae

Kifune, 1979

938B8B92-5D37-56D9-B088-A0CAAEC2696B

Xenos peruensis Kifune, 1979: 408.

##### Host.

*Polisteslanio* (Fabricius, 1775) (Kifune 1979).

##### Distribution.

Peru (Kifune 1979).

#### ﻿ Xenos provesparum

Taxon classificationAnimaliaStrepsipteraXenidae

Kifune, 1986

0B26BE9F-B3EE-5439-8468-8F2C830F7F1F

Xenos provesparum Kifune, 1986: 84.

##### Hosts.

*Provespaanomala* (Saussure, 1854); *Provespanocturna* Vecht, 1935 (Kifune 1986).

##### Distribution.

Indonesia: Sumatra, Padang (Kifune 1986); Thailand (Kifune and Yamane 1998).

#### ﻿ Xenos ropalidiae

Taxon classificationAnimaliaStrepsipteraXenidae

(Kinzelbach, 1975) comb. nov.

6499DCBA-D2B9-58B1-B4F6-648411494596

Pseudoxenos ropalidiae Kinzelbach, 1975: 69.

##### Hosts.

*Ropalidiacincta* (Lepeletier, 1836); *Ropalidiafulvopruinosa* (Cameron, 1906); *Ropalidiamarginata* (Lepeletier, 1836) (as *Ropalidiaferruginea* F.); *Ropalidianobilis* (Gerstäcker, 1857); *Ropalidiavariegata* (Smith, 1852) (Kinzelbach 1975; Cook 2019); *Ropalidiamalayana* (Cameron 1903) (Benda et al. 2021).

##### Distribution.

Democratic Republic of Congo; India; Indonesia: Java; Papua New Guinea; Philippines (Kinzelbach 1975; Cook 2019); Laos; Nepal; Malaysia (Benda et al. 2021).

##### Note.

Benda et al. (2021) proposed three lineages possibly representing separate species. More comprehensive sampling and detailed study are necessary.

#### ﻿ Xenos rostratus

Taxon classificationAnimaliaStrepsipteraXenidae

Trois, 1984b

E34D1F4A-6051-5E1D-A30C-6C3B6DC0BD4A

Xenos rostratus Trois, 1984b: 24.

##### Hosts.

*Polistesbillardieriruficornis* Saussure, 1853 (as *Polistesruficornisruficornis* Saussure, 1853); *Polistesbillardieribiglumoides* Ducke, 1904 (as *Polistesruficornisbiglumoides* Ducke, 1904) (Trois 1984b).

##### Distribution.

Brazil, Sao Paulo; Paraguay, Villarcia; Peru, Ayacucho (Trois 1984b); Argentina (Benda et al. 2021).

#### ﻿ Xenos rubiginosi

Taxon classificationAnimaliaStrepsipteraXenidae

(Pierce, 1909)

CA9B9468-6304-5E48-827F-002DD4D41995

Acroschismus rubiginosi Pierce, 1909: 132.
Xenos rubiginosi (Pierce, 1909) (new combination by Bohart 1941).

##### Host.

*Polistescarolina* (Linnaeus, 1767) (as *Polistesrubiginosus* Lepeletier) (Pierce 1909).

##### Distribution.

USA: Louisiana (Pierce 1909).

#### ﻿ Xenos stuckenbergi

Taxon classificationAnimaliaStrepsipteraXenidae

Pasteels, 1956

2F0B1099-2378-5EDF-B30F-040871CEC70B

Xenos stuckenbergi Pasteels, 1956: 441.

##### Host.

*Polistesmarginalis* (Fabricius, 1775) (Pasteels 1956).

##### Distribution.

RSA: Natal (Pasteels 1956).

#### ﻿ Xenos vesparum

Taxon classificationAnimaliaStrepsipteraXenidae

(Rossi, 1793)

F6A44F9A-095C-5F5C-B6AC-3ADD655491C0

Ichneumon vesparum Rossi, 1793: 49.
Xenos vesparum (Rossi, 1793) (new combination by Rossi 1794).
Xenos rossii Kirby, 1813 (synonymized by Saunders 1872).
Xenos jurinei Saunders, 1872 (synonymized by Kinzelbach 1971b).
Xenos minor Kinzelbach, 1971a, syn. nov.

##### Hosts.

*Polistesalbellus* Giordani Soika, 1976; *Polistesassocius* (Kohl, 1898); *Polistesbiglumis* (Linnaeus, 1758); *Polistesdominula* (Christ, 1791) (as *Vespagallica* Linnaeus and *Polistesgallicus* Linnaeus); *Polistesgallicus* (Linnaeus, 1767) (as *Polistesfoederatus* Kohl, 1898); *Polistesnimpha* (Christ, 1791); *Polistessulcifer* (Zimmerman, 1930); *Polistessemenowi* (Morawitz, 1889); *Vespulavulgaris* (Linnaeus,1758); *Ropalidia* sp. (Kinzelbach 1971a, 1978; Benda et al. 2021).

##### Distribution.

Italy (Rossi 1793, 1794); Palearctic (Kinzelbach 1978; Benda et al. 2021); India (Benda et al. 2021).

##### Note.

*Xenosminor* is synonymized under *X.vesparum* based on the results of a recent molecular phylogeny of Benda et al. (2021). Specimens morphologically corresponding to *Xenosminor* were nested within the lineage of *Xenosvesparum*. The former taxonomy was probably misled by the large phenotypic variability of *Xenosvesparum*, corresponding to different host taxa (smaller specimens of *X.vesparum* are associated with smaller individuals of *Polistes* spp.).

#### ﻿ Xenos yamaneorum

Taxon classificationAnimaliaStrepsipteraXenidae

Kifune & Maeta, 1985

97781200-EDED-5CF9-BC2C-573DE36C5BE8

Xenos yamaneorum Kifune & Maeta, 1985: 430.

##### Host.

*Polistesgigas* Kirby, 1826 (Kifune and Maeta 1985).

##### Distribution.

Taiwan (Kifune and Maeta 1985).

#### ﻿ Xenos yangi

Taxon classificationAnimaliaStrepsipteraXenidae

Dong, Liu & Li, 2022

DFD2454D-E72D-565E-B5E6-20A3DB7F6C22

﻿Xenosyangi Dong, Liu & Li, 2022: 15.

##### Hosts.

*Vespavelutina* Lepeletier, 1836 and *Vespabicolor* Fabricius, 1787 (Dong et al. 2022).

##### Distribution.

China: Yunnan (Dong et al. 2022).

#### ﻿ Xenos zavattarii

Taxon classificationAnimaliaStrepsipteraXenidae

(Pierce, 1911)

96E9213A-E263-5E5F-8507-72E96C912D05

Belonogastechthrus zavattarii Pierce, 1911: 498.
Xenos zavattarii (Pierce, 1911) (new combination by Bohart 1941).

##### Hosts.

*Belonogasterlateritia* Gerstaecker, 1857 (as *Belonogasterelegans* Gerstaecker, 1857); *Belonogasterjuncea* (Fabricius, 1781); (Pierce 1911; Kinzelbach 1978).

##### Distribution.

Uganda: Butiti (Pierce 1911); Angola; Democratic Republic of Congo; Liberia; Libya: Tripolis (Pasteels 1950; Luna de Carvalho 1956; Kinzelbach 1978); Central African Republic; Ethiopia; Yemen: Socotra (Benda et al. 2021).

##### Note.

Benda et al. (2021) reported two lineages that could be considered as separate species.

### ﻿Key to genera of Xenidae based on the female cephalothorax

**Table:** 

1	Head and prothorax on ventral side completely separated by birth opening (Fig. 8A). Dorsal labral field elliptic, ~ 2× wider than medially long, distinctly protuberant (dlf, Fig. 8A)	***Paragioxenos* Ogloblin (Australia; *Paragia* spp.)**
–	Head and prothorax on ventral side separated by birth opening medially and by suture laterally (Fig. 1A). Dorsal labral field at least 3× wider than long in midline (dlf, Fig. 3A)	**2**
2	Maxillae strongly sclerotized, partially fused with labial area, not prominent, appearing connected proximally along birth opening (Fig. 10A, 20C). Cephalothorax mostly lightly colored	**3**
–	Sclerotization of maxillae different. Maxillae partly fused with labium or prominent. Cephalothorax variously colored	**5**
3	Mandible distinctly protruding from mandibular capsule, reaching or slightly projecting beyond anterior edge of head (md, Fig. 10A). Anterior part of maxilla pointed (mx, Fig. 10A)	***Nipponoxenos* Kifune & Maeta (East Asia; *Vespula* spp.)**
–	Mandible not protruding from mandibular capsule, anterior part of maxilla rounded (mx, Fig. 20C)	**4**
4	Border between clypeus and labrum always distinct (sbcl, Fig. 47D)	***Xenos* Rossi, in part (Old and New World; Vespini, Polistini, Mischocyttarini, Ropalidiini)**
–	Clypeal region not clearly delimited from labral area, more or less fused (Fig. 22D)	***Brasixenos* Kogan & Oliveira (New World; Epiponini)**
5	Prosternal extension anteriorly with conspicuous extensive pale spot, sometimes associated with cuticular impression (pps, Figs 35C, 37A). Maxillary base continued anterolaterally as a distinct submaxillary groove (smxg, Fig. 37A)	***Sphecixenos* gen. nov. (Old World and Australia; Sphecidae)**
–	Prosternal extension different. Submaxillary groove distinct (smxg, Fig. 18A) or indistinct (smxg, Fig. 53A)	**6**
6	Maxillae prominent (Figs 14E, 41E)	**7**
–	Maxillae not prominent, partially or completely fused with head capsule, rarely slightly raised	**10**
7	Mandibular tooth very wide basally, reaching area of mandibular bulge. Tooth base ventrally covered with small depressions continuous with several rows of spines (md, mdt, Fig. 14E)	***Tachytixenos* Pierce (Old World; *Tachytes* spp.)**
–	Mandibular tooth narrow or only slightly widened	**8**
8	Vestige of antenna preserved as cavity, additional rounded plates rarely present (a, Fig. 17D)	***Paraxenos* Saunders, in part (Old World and Australia; *Bembix* spp., *Stizus* spp.)**
–	Vestige of antenna different	**9**
9	Cephalothorax conspicuously convex, round (Fig. 43C), highly elliptic in cross-section. Dorsal labral field raised, protruding anteriorly (dlf, Fig. 45D)	***Tuberoxenos* gen. nov. (Afrotropic + Palearctic; Sphecidae)**
–	Cephalothorax more flattened, not or indistinctly bulging (Fig. 36C), more flattened in cross-section. Dorsal labral field flat (dlf, Fig. 38D)	***Pseudoxenos* Saunders, in part (Palearctic; Odynerini)**
10	Vestige of antenna preserved as cavity, additional rounded plates rarely present	***Paraxenos* Saunders, in part (Old World and Australia; *Bembecinus* spp.)**
–	Vestige of antenna different	**11**
11	Two distinct dark spots present mesally on border between head and prothorax (sbhp, Fig. 32D). Thoracic segments conspicuously sclerotized laterally from dorsal side (tx, Fig. 32D). Lateral parts of abdomen posterior to spiracles pale (asI, Fig. 32D). Clypeal area very distinctly delimited from labral area (sbcl, Fig. 34D)	***Macroxenos* Schultze (Australasian and Indomalayan regions; Odynerini)**
–	Combination of characters different	**12**
12	Sensilla on clypeal lobe extended to ventral side, often present close to clypeolabral border (cls, Fig. 53D)	***Xenos* Rossi, in part (Old and New World; Vespini, Polistini, Mischocyttarini, Ropalidiini)**
–	Position of sensilla different	**13**
13	Rudiments of antennal torulus only rarely preserved. Distributed in the Old World or Australia	**14**
–	Rudiments of antennal torulus usually present (at, Figs 25C, 29D). Distributed in the New World distribution	**15**
14	Meso-metathoracic segmental border constricted laterally (sbmm, Fig. 47C, D). Dorsal labral field raised (dlf, Fig. 49D), when flat, then narrower laterally than medially (dlf, Fig. 3A)	***Deltoxenos* gen. nov. (Old World + Australia; Eumeninae)**
–	Meso-metathoracic segmental border not constricted laterally (Fig. 39C, D). Dorsal labral field flat, laterally as long as medially (dlf, Fig. 41C, D)	***Pseudoxenos* Saunders, in part (Palearctic; Eumeninae)**
15	Frontal region conspicuously covered with frontal papillae (frp, Fig. 25F). Periantennal area small, indistinct, suppressed by supra-antennal sensillary field (paa, Fig. 25C). Prosternum connected to head on same plane, but elevated anteriorly (pst, lehc, Fig. 25A)	***Leionotoxenos* Pierce (New World; Odynerini)**
–	Frontal region smooth or very slightly wrinkled, without papillae (fr, Fig. 30B). Periantennal area expanded, sometimes raised, smooth. Distance between antennal area and supra-antennal sensillary field relatively large (paa, Fig. 29C). Prosternum more elevated above head along entire cephalo-prothoracic border (pst, lehc, Fig. 29A)	***Eupathocera* Pierce (New World; Sphecidae, Crabronidae, Zethinae, *Pachodynerus* spp.)**

### ﻿Key to genera of Xenidae based on the cephalotheca of the male puparium

Cephalothecae of *Paragioxenos* and *Macroxenos* unknown.

**Table:** 

1	Maxilla scarcely recognizable, fused with cephalotheca (mx, Fig. 23E). Vestige of palp distinct in optical microscope but hardly visible in SEM micrographs (mxp, Fig. 23A, D)	***Brasixenos* Kogan & Oliveira (New Word; Epiponini)**
–	Maxilla distinct, prominent (e.g., Fig. 6A, B)	**2**
2	Diameter of genae between maxillary base and compound eye relatively small, ca. as large as diameter of vestigial antenna (gn, Fig. 11A). Vestigial antenna very large (a, Fig. 11A). Cephalotheca always pale, only clypeus and genae dark	***Nipponoxenos* Kifune & Maeta (East Asia; *Vespula* spp.)**
–	Diameter of genae between maxillary base and compound eye distinctly larger than diameter of vestigial antenna (gn, Fig. 5A). Vestigial antenna smaller, cephalotheca usually darker	**3**
3	Diameter of genae between maxillary base and compound eye ~ 1.5× larger than diameter of vestigial antenna (gn, Fig. 42D)	**4**
–	Diameter of genae between maxillary base and compound eye at least 2× larger than diameter of vestigial antenna (gn, e.g., Fig. 15D)	**5**
4	Occipital bulge present (ob, Fig. 42D). Frontal region distinctly deformed by frontal impression (fi, Fig. 42D). Paired furrows of supra-antennal sensillary field absent. Cephalotheca elliptic	***Pseudoxenos* Saunders (Palearctic; Odynerini)**
–	Occipital bulge absent. Frontal impression absent. Paired furrows of supra-antennal sensillary field present (fssf, Fig. 46A, D). Cephalotheca nearly circular in frontal view	***Tuberoxenos* gen. nov., in part (Afrotropic and Palearctic regions; Sphecidae)**
5	Paired furrow of supra-antennal sensillary field present (fssf, Figs 15A, D, 19A, D)	**6**
–	Paired furrow of supra-antennal sensillary field absent (Figs 5A, 6A)	**8**
6	Mandibular tooth wide basally, reaching mandibular bulge (mdt, Fig. 15E). Tooth base with small depressions continuous with several rows of spines (mdts, Fig. 14E)	***Tachytixenos* Pierce (Old World; *Tachytes* spp.)**
–	Mandibular tooth narrow or slightly widened (mdt, Fig. 19E)	**7**
7	Cephalotheca elliptic in frontal view (Fig. 19A)	***Paraxenos* Saunders (Old World and Australia; *Bembix* spp., *Stizus* spp.)**
–	Cephalotheca nearly circular in frontal view (Fig. 46A)	***Tuberoxenos* gen. nov., in part (Afrotropic and Palearctic regions; Sphecidae)**
8	Cephalotheca nearly circular in frontal view (Figs 38A, 46A)	**9**
–	Cephalotheca elliptic in frontal view (e.g., Figs 50A, 54A)	**10**
9	Vestigial antenna with diameter subequal to width of mandible (a, md, Fig. 31E). Mandible directed anteromedially	***Eupathocera* Pierce (New World; Sphecidae, Crabronidae, Zethinae, *Pachodynerus*)**
–	Vestigial antenna with diameter smaller than width of mandible (a, md, Fig. 38E). Mandible directed almost medially	***Sphecixenos* gen. nov. (Old World + Australia; Sphecidae)**
10	Frontal fissure very distinct (fi, Fig. 27D). Maxilla prominent, at least 1.5× longer than wide at base (mx, Fig. 27E)	***Leionotoxenos* Pierce (New World; Odynerini)**
–	Frontal fissure quite indistinct or nearly absent (fi, Fig. 6A, 54D). Maxilla not distinctly elongated, at most 1.5× longer than basally wide (mx, Figs 50E, 54E)	**11**
11	Occipital bulge present, well- developed (ob, Figs 6A, 50D). Cephalotheca with a pattern of pale and dark shades (Figs 5A, 50A)	***Deltoxenos* gen. nov. (Old World + Australia; Eumeninae)**
–	Occipital bulge strongly reduced or missing (ob, Fig. 54D). Cephalotheca mostly dark (Fig. 54A)	***Xenos* Rossi (Old and New World; Vespini, Polistini, Mischocyttarini, Ropalidiini)**

## ﻿Discussion

The results of this study are mainly compared with external characters of the cephalothorax of females of *Xenosvesparum* (Richter et al. 2017) and *Stylopsovinae* (Löwe et al. 2016). Characters of the cephalotheca of the male puparium are compared with Kinzelbach (1971b). The morphology of adult males is also potentially valuable for the taxonomy of Xenidae. However, it was not considered here, as only few well-preserved specimens were available. Likewise, the morphology of the first instars can be useful for taxonomy, especially the well-developed pattern of setae (Pohl 2002; Straka et al. 2014). However, these features were not included in this study due to limited material.

### ﻿Cephalothorax of the female

A conspicuous autapomorphy of stylopidian females is the secondary tagmosis with an anterior cephalothorax which is protruding from the host and a large, sack-shaped posterior body region which remains hidden in the body lumen of the abdomen (Löwe et al. 2016). This profound structural transformation is closely linked with the endoparasitic lifestyle. This also includes the reduction of antennae, mouthparts, compound eyes, and legs, which are preserved as rudiments or completely lost (Kinzelbach 1971b; Pohl and Beutel 2005). The wings of females are already absent in the ground plan of Strepsiptera (Pohl and Beutel 2005, 2008).

The homology of the cephalothorax was discussed in previous studies. Kinzelbach (1971b) was the first who suggested that it is formed by fusion of the head, thorax, and the anterior part of abdominal segment I, which bears a pair of functional spiracles. This interpretation was later supported by comprehensive treatments based on modern techniques (Pohl and Beutel 2005, 2008; Löwe et al. 2016; Richter et al. 2017). Alternatively, it was suggested that the cephalothorax comprises the head and thorax (e.g., Lauterbach 1954), or even only the head and prothorax (Hrabar et al. 2014; Kathirithamby et al. 2015). Recognizable segmental borders and different cuticular microstructures clearly support the concept proposed by Kinzelbach (1971b) and Richter et al. (2017). The segmental border between the head and prothorax is distinctly visible on the ventral side, demarcated by the birth opening and often by lateral sutures. However, the latter are absent in many members of Stylopidae, as for instance in some *Stylops* (Löwe et al. 2016) or *Halictoxenos* (Straka et al. 2006). An exception among Xenidae is the genus *Paragioxenos*, with the head and prothorax completely separated by the birth opening on the ventral side, as it is also the case in *Rozenia* of Stylopidae (Straka et al. 2014). On the dorsal side, the head and prothorax of females of Xenidae are completely fused, but a border region is still indicated by changes in the cuticular surface or by pigmented stripes. This is in contrast to *Stylops*, where the border is delimited by a distinct furrow (Löwe et al. 2016). The pro-mesothoracic and meso-metathoracic borders are usually more distinct on the ventral side as it was previously shown in *Xenos* and *Stylops* (Löwe et al. 2016; Richter et al. 2017), but with differences among genera or species. The prosternum is very variable in Xenidae. It can be variously modified, with a prosternal swelling present in some cases (*Paragioxenos*, *Paraxenos*) or with a protruding margin overlapping with the maxillolabial area and the posterior part of the mandibles (*Macroxenos*, *Tuberoxenos*, *Xenos*). The shape of the meso- and metathorax is mostly transverse and unmodified, but in some cases constricted laterally, resulting in an unusual proximity of the head and abdominal spiracles (*Brasixenos*, some *Macroxenos*).

The distinct constriction in the middle region of abdominal segment I, the zone of contact with the host cuticle, is distinct in all genera of Xenidae. Functionally this can be explained as an adaptation preventing the exposed anterior body from slipping back into the host body cavity (Löwe et al. 2016). Richter et al. (2017) suggested that cuticular spines on abdominal segment I have probably the same function. These structures are apparently missing in *Stylops*. Cuticular spines occur in most genera of Xenidae and are functionally replaced by a very roughly sculptured lateral cuticle in cases where they are missing. In some species of *Brasixenos*, *Eupathocera* and *Sphecixenos*, the area below the abdominal spiracles extrudes as a spiracular corner, in some cases very distinct, as in *Rozenia* (Straka et al. 2014). Spiracles are functional in all Xenidae with variable orientation and position.

The more or less flattened ellipsoid shape of the cephalothorax of all species of Xenidae stabilizes its position between the host abdominal segments. Kinzelbach (1971b) interpreted this as an adaptation to a mechanical strain caused by the host cuticle. It is noteworthy in this context that the male puparium is not flattened. Apparently, the adaptation of the female is more advanced, likely due to a stronger selective pressure caused by permanent endoparasitism (Pohl and Beutel 2008).

The function of the fissure-shaped mouth opening is the uptake of the host hemolymph by the secondary larvae (Giusti et al. 2007). It is well-developed and sclerotized along the margin in all examined species of Xenidae, but obviously non-functional after the extrusion of the cephalothorax from the host.

The birth opening between the head and prosternum is the site where copulation and the release of the first instar larvae take place (Pohl and Beutel 2008). This structure is an autapomorphy of Stylopiformia (Pohl and Beutel 2005). It was shown that the membranous cuticle of this region is perforated by the penis during copulation in *Stylops* (Peinert et al. 2016). In the case of *Xenos*, Kathirithamby et al. (2015) hypothesized that the brood canal membrane is ruptured during the super-extrusion of the cephalothorax, thereby facilitating the release of pheromones during mate signaling. However, a perforation by the male penis during copulation is also possible (Beani et al. 2005).

Cephalic structures are always distinctly reduced. Richter et al. (2017) interpreted the assemblages of circular fields on the dorsal side of head capsule as vestiges of antennae in *Xenosvesparum*. We confirmed the presence of vestigial antennae across Xenidae in various stages of reduction. They are preserved as a remnant of an antennal torulus in *Leionotoxenos* and *Eupathocera*, with rounded plates and vestigial antennal setae, whereas only a simple groove or cavity is present in *Brasixenos* and *Paraxenos*. Previously, a vestigial antenna was ascribed to the entire Stylopidia (Kinzelbach 1971b, Pohl and Beutel 2005). Even though this is very likely part of the groundplan, the antenna is completely reduced in *Stylops* (Löwe et al. 2016).

The vestigial maxillae are very variable in Xenidae, providing valuable characters for the identification of genera and species. They are variably sculptured, prominent in *Tachytixenos*, *Tuberoxenos* or *Eupathocera*, or completely fused with the labial area in *Brasixenos*. However, any degree of reduction occurring in Xenidae does not match the nearly complete absence in genera of Stylopidae, such as *Stylops* (Löwe et al. 2016), *Rozenia* (Straka et al. 2014) or *Halictoxenos* (Straka et al. 2006). Maxillary bases adjacent with the birth opening can be medially fused as in *Paragioxenos*. Sclerotized and fused maxillae are very conspicuous on a pale head capsule as in *Nipponoxenos* and *Brasixenos*. The presence of a submaxillary groove is probably correlated with a prosternal extension projecting into the head capsule. It is missing in *Paragioxenos* where this structure is absent, but conspicuously developed in some genera with a well-developed extension of the prosternum. A similar condition was not found in *Stylops* (Löwe et al. 2016).

In contrast to the maxillae, the mandibles are well-developed in all Xenidae. They are the only movable cephalic appendages with a flexible articulatory membrane, and extended and flexed by the two antagonistic craniomandibular muscles. Shortly after the emergence from the host, the entire surface of the cephalothorax is sclerotized, and the mandibles are immobilized (Richter et al. 2017). According to Lauterbach (1954), the mandibles help penetrating the host membrane during the extrusion. They are equipped with a tooth, which is also present in *Stylops* (Löwe et al. 2016). It can be used as a character for distinguishing related genera (*Tachytixenos*, *Paraxenos*) and its shape can also be species-specific in Xenidae (Nakase and Kato 2013). The labrum is not distinctly developed as a separate cephalic appendage, but only preserved as dorsal and ventral labral fields. The latter was described as a “semicircular structure” in *Stylops* (Löwe et al. 2016) or a “semicircular field possibly of labral origin” in *Xenosvesparum* (Richter et al. 2017). This structure is variably shaped in Xenidae, not always semicircular, and arguably formed by an everted epipharynx as hypothesized by Lauterbach (1954). The dorsal field bears several rows of spine-like sensilla (setae) in all genera of Xenidae. They were described by Richter et al. (2017) and are probably also present in *Stylops* (Löwe et al. 2016). Sensilla are also present on a narrow clypeal area and on a supra-antennal field near the vestigial antennae. Clypeal sensilla were mentioned in Richter et al. (2017) for the first time as “sensilla on the anterior head capsule”. Possible homologous structures were described as the “field of sensilla” in *Malayaxenos* (Corioxenidae) (Pohl and Beutel 2005). A supra-antennal field of sensilla was described for the first time by Kinzelbach (1971b) as “Pigmentzelle” on the female cephalotheca and female of Mengenillidae. It is conceivable that the sensory function of these organs facilitates the orientation of secondary larvae in the body lumen of the host and the proper extrusion from the host abdomen.

### ﻿Cephalotheca of the male puparium

The cephalotheca is the anterior part of the puparium, where the male emerges after extrusion from the host and completes its development. The puparium is formed by the sclerotized exuvia of the male secondary larvae. The cephalotheca is homologous to the head capsule of the cephalothorax of the female (Kinzelbach 1971b). Compared to the female cephalothorax and the adult male, the external morphology of the male cephalotheca was very poorly studied previously. Kinzelbach (1971b) presented the first comparison of cephalothecae across the entire Strepsiptera, with descriptions of many features. It turned out that the cephalotheca can provide important and practical characters for species delimitation (Nakase and Kato 2013). In Xenidae, it is apparently more convenient to work with cephalothecae than with males enclosed in the puparium, as the latter are often immature, unsclerotized, or poorly preserved, especially in older museum material. The cephalothecal characters are also well visible externally on the puparium extruding from the host abdomen, without prior dissection.

The most striking feature of the cephalotheca, in contrast to the female cephalothorax, is the presence of compound eyes. Individual ommatidia are usually visible on the pale background of the ocular area as in *Halictoxenos* or *Myrmecolax* (Soon et al. 2012; Nakase et al. 2014). A completely dark ocular area occurs only in some species of *Xenos*. In lateral view, the cephalotheca appears rounded or pointed, with the clypeus and its sensilla placed apically. In contrast to the apical region of the female cephalothorax, the clypeus of the male cephalotheca is distinctly developed, with an epistomal suture separating it from the frontal region. The cephalothecal supra-antennal sensillary field is more conspicuous and usually bulging in contrast to the flat one on the female cephalothorax. We assume that these structural elements are homologous in both sexes, that they have a sensorial function, and that they facilitate the orientation of the male and female secondary larvae in the host body lumen.

The vestigial antennae are less reduced than in the female cephalothorax. They vary mainly in size, whereas the shape is variable in females. An antennal torulus is always distinctly developed, but in some cases interrupted. A scapus and pedicellus can be distinguished in the genus *Myrmecolax* (Myrmecolacidae) according to Kathirithamby et al. (2010) and Nakase et al. (2014). However, the homology of these basal antennal segments of immature stages is highly uncertain in Holometabola (e.g., Beutel et al. 2011). The mandibles are well developed, with homologous features in secondary larvae of both sexes. Nakase and Kato (2013) found the same shape of mandibular tooth on the male and female secondary larvae of *Xenos*, which is constant intraspecifically and could be easily used for species identification. We found a specific shape of the mandibular tooth characteristic for the genus *Tachytixenos*. The maxillae of male cephalotheca have not undergone such diverse changes and modifications as in female cephalothorax in Xenidae. In most genera, they are well developed except for *Brasixenos* with maxillae completely fused to the head capsule. Kinzelbach (1971b) even presented that some genera of Halictophagidae could have preserved the articulation of maxillae on the male cephalotheca.

### ﻿Taxonomy and host specialization of Xenidae

The monophyly of Xenidae is well supported by morphological and molecular data (Pohl and Beutel 2005; McMahon et al. 2011). We have newly delimited 13 genera of this family with a total of 119 described species. Although we did not deal with a precise species delimitation of all material available, we approximately estimated at least 70 undescribed species, which represents more than half of the known diversity (Table 1). This estimation is very conservative. It is based on a comprehensive phylogenetic analysis (Benda et al. 2021) and material examined by the authors in various collections. Part of this material is prepared for species descriptions in subsequent publications. However, small genera with many autapomorphies, which would render other genera paraphyletic, have not been found.

**Table 1.** Overview of Xenidae genera with general information on distribution, hosts, and the number of described species; a conservative estimate of the number of undescribed species is also provided.

Genus	Distribution	Hosts	Number of species	Number of undescribed species
*Paragioxenos* Ogloblin, 1923	Australia	*Paragia* (Vespidae: Masarinae)	1	0
*Nipponoxenos* (Kifune & Maeta, 1975), stat. res.	East Asia	*Vespula* (Vespidae: Vespinae)	1	0
*Tachytixenos* Pierce, 1911, stat. res.	Old World	*Tachytes* (Crabronidae: Crabroninae)	1	4
*Paraxenos* Saunders, 1872	Old World, Australasian	*Bembecinus*, *Bembix*, and *Stizus* (Bembicidae: Bembicinae)	13	7
*Brasixenos* Kogan & Oliveira, 1966, stat. res.	New World	Epiponini (Vespidae: Polistinae)	7	7
*Leionotoxenos* Pierce, 1909, stat. res.	New World	Odynerini (Vespidae: Eumeninae)	14	3
*Eupathocera* Pierce, 1908, stat. res.	New World	Sphecinae, Ammophilinae (Sphecidae); *Tachytes* (Crabronidae: Crabroninae); *Zethus* (Vespidae: Zethinae); *Pachodynerus* (Vespidae: Eumeninae)	16	8
*Macroxenos* Schultze, 1925, stat. res.	Australasian, Indomalayan	Odynerini (Vespidae: Eumeninae)	2	3
*Sphecixenos* gen. nov.	Old World, Australasian	*Sphex*, *Isodontia* (Sphecidae: Sphecinae); *Sceliphron* (Sphecidae: Sceliphrinae); *Chlorion* (Sphecidae: Chloriontinae)	12	1
*Pseudoxenos* Saunders, 1872	Palearctic	Odynerini (Vespidae: Eumeninae)	7	2
*Tuberoxenos* gen. nov.	Afrotropical, Palearctic	*Ammophila*, *Podalonia* (Sphecidae: Ammophilinae); *Prionyx* (Sphecidae: Sphecinae)	5	8
*Deltoxenos* gen. nov.	Old World, Australasian	Eumenini, Odynerini (Vespidae: Eumeninae)	7	17
*Xenos* Rossi, 1793	Old and New World	Vespini (Vespidae: Vespinae); Polistini, Mischocyttarini, Ropalidiini (Vespidae: Polistinae)	33	11

The monotypic genus *Paragioxenos* was described by Ogloblin (1923) from Australia and has never been reported since. Although an early divergence was assumed, its phylogenetic position is still unknown. The male was characterized by a specific shape of the penis. The characterization of the female was based on the condition of the border between the head and prothorax, described by Ogloblin (1923: 46) as a “transversal slit, which separates front part of cephalothorax not curved, but simply rounded”. Additionally, Kinzelbach (1971b) pointed out to the unique shape of the maxillae. Our own study of the type material suggests a clear delimitation of this genus by the shape of the birth opening, features of the mandibles and dorsal labral field. Fresh material for extraction of DNA sequences is urgently required. Analyses of molecular data would likely reveal the phylogenetic position of this enigmatic genus with a unique specialization on pollen wasps.

The monotypic *Nipponoxenos* was originally described as a subgenus of *Xenos* from the genus *Vespula* Thomson in East Asia (Kifune and Maeta 1975). The female was characterized by almost straight and anteriorly tapering lateral margins of the cephalothorax, slightly constricted just anterior to the spiracles. The defining feature of the male was a typical penis with prominent dorsal spine, pickaxe-shaped in lateral view. Benda et al. (2021) found *Nipponoxenos* as the earliest diverging group, sister to all other Xenidae or sister to *Tachytixenos* and *Paraxenos*.

The monotypic genus *Tachytixenos* was described by Pierce (1911) from India by a unique association with wasp hosts of the genus *Tachytes*. Later Kinzelbach (1978) cited supplementary records of stylopized *Tachytes* from the Palearctic and Indomalayan regions. We re-establish *Tachytixenos* as a genus with a wider distribution than expected, and estimate existence of at least four undescribed species. Apart from *Tachytixenos*, Kinzelbach (1971b, 1978) synonymized several additional genera with *Paraxenos*. In contrast, Benda et al. (2019, 2021) delimited *Paraxenos* as a lineage with a distribution in the Old World and the Australasian region, and parasitizing exclusively species of Bembicinae. We provide a redescription of *Paraxenos* based on new characters, and report at least seven undescribed species. The genus *Brasixenos* was expected as closely related to *Xenos*, but Benda et al. (2019) revealed the group as a separate lineage parasitizing social Epiponini and unrelated to *Xenos*. *Brasixenos* is well delimited by the female cephalothorax and male cephalotheca. A revision of adult males is needed as well as an evaluation of male diagnoses provided by Kogan and Oliveira (1966). We expected the diversity within the genus *Brasixenos* to be at least twice higher than the number of described species.

Previously, several genera were described by Pierce (1908, 1909, 1911, 1919), mainly from the New World. He suggested that a new genus of Strepsiptera should be established if it utilizes a different host genus. We restored the genera *Leionotoxenos* and *Eupathocera* for two sister clades from the New World, revealed by Benda et al. (2019, 2021). Although *Leionotoxenos* is specialized on solitary wasps of the tribe Odynerini, *Eupathocera* is more generalist utilizing mainly species of Sphecidae but rarely the subfamilies Crabroninae, Zethinae or Eumeninae.

The genus *Macroxenos* was described by from the Philipines as a parasite of potter wasps of *Anterhynchium* (Schultze 1925). Although Bohart (1937) synonymized it with *Pseudoxenos*, Benda et al. (2019) found a remarkable lineage with an Australasian origin that dispersed to the Indomalayan region. We classify it as *Macroxenos* and report at least three undescribed species. Nevertheless, we assume that diversity of this genus is much higher because of a high morphological variability of species, especially in Australasian region. The lineage named here as *Sphecixenos* gen. nov. was revealed by Benda et al. (2019, 2021) who found it as a separate clade with an Afrotropical origin, dispersed into the Indomalayan and Australasian regions. Its main hosts are wasps of the genus *Sphex*, less often *Isodontia*, *Sceliphron*, and *Chlorion*.

*Pseudoxenos* was described by Saunders (1872) with the description of several species parasitizing Odynerini in European Mediterranean. The taxonomic validity of some described species within *Pseudoxenos* from the West Palearctic region is questionable and a more detailed study is necessary for the clarification of interspecific relationships (Cook 2019; Benda et al. 2021). In the phylogenetic tree from Benda et al. (2021) a sister-group relationship between a lineage parasitizing *Pachodynerus* and another Palearctic lineage parasitizing Eumenini was suggested, but the branch support values were very low, and this relationship is not supported by morphology. The latter lineage is provisionally included here in *Deltoxenos* gen. nov. and the lineage from *Pachodynerus* is provisionally included in *Eupathocera* based on morphology. More comprehensive sampling and a robust genomic analysis are necessary for the clarification of systematics and phylogeny of these taxa. *Tuberoxenos* gen. nov. is described here as the sister genus to *Pseudoxenos*, restricted to the Afrotropical and Palearctic regions and associated mainly with *Ammophila* and *Podalonia*, very rarely *Prionyx*.

*Deltoxenos* gen. nov. utilizes a diverse range of hosts from Odynerini and Eumenini (Vespidae: Eumeninae) (Benda et al. 2021). Only few species were described from the Afrotropical and Palearctic regions, but we estimate more than twice as many species than currently described. The distribution of the genus is wider, spanning over the Old World and the Australasian region. Benda et al. (2019) suggested a unique evolution of this lineage including a dispersion of the group from the Afrotropics through the Palearctic and Indomalayan regions to Australasia. This dispersion was probably initialized by the switch from Odynerini to Eumenini that provided an opening of a new host niche and an opportunity to utilize a wide range of host taxa.

*Xenos* was the first named genus in Strepsiptera, although it took some time before the order was formally introduced (Cook 2019; Rossius 1794). We have redescribed *Xenos* by a combination of characters as parasites of four tribes of social Vespidae. In comparison to other xenid genera, *Xenos* is the only genus distributed both in the Old and the New World, but its origin and expansion is not well clarified (Benda et al. 2019). It represents the most species-rich genus of Xenidae with 32 described species and at least 11 undescribed species.

The previous classification of genera of Xenidae by Pierce (1908, 1909, 1911, 1919) implied a specialization on the level of host genus, while the classification by Kinzelbach (1971b) suggested a specialization on the level of host family or subfamily. Our generic concept combines both approaches and is more complex. Some representatives of the current genera parasitize only one host genus (e.g., *Paragioxenos*, *Nipponoxenos* and *Tachytixenos*), whereas others can even utilize hosts from three families (e.g., *Eupathocera*). The species diversity of a lineage depends on the ability to utilize new hosts which would also facilitate the dispersion and increase the range of distribution (Benda et al. 2019).

## Supplementary materials

Supplementary material 1 Table S1 Data type occurences Explanation note Voucher names, hosts, and collection localities of the samples. https://binary.pensoft.net/file/669377This dataset is made available under the Open Database License (http://opendatacommons.org/licenses/odbl/1.0/). The Open Database License (ODbL) is a license agreement intended to allow users to freely share, modify, and use this Dataset while maintaining this same freedom for others, provided that the original source and author(s) are credited.Daniel Benda, Hans Pohl, Yuta Nakase, Rolf Beutel, Jakub Straka
